# Identification and Comparative Expression Profiles of Candidate Olfactory Receptors in the Transcriptomes of the Important Egg Parasitoid Wasp *Anastatus japonicus* Ashmead (Hymenoptera: Eupelmidae)

**DOI:** 10.3390/plants12040915

**Published:** 2023-02-17

**Authors:** Hai-Xia Zhan, Lan Li, Feng-Qi Li, Lian-Sheng Zang

**Affiliations:** National Key Laboratory of Green Pesticide, Key Laboratory of Green Pesticide and Agricultural Bioengineering, Ministry of Education, Guizhou University, Guiyang 550025, China

**Keywords:** antennal transcriptome, abdominal transcriptome, odorant receptors, ionotropic receptors, fruit crops

## Abstract

*Anastatus japonicus* Ashmead is an egg parasitoid wasp important for the biological control of fruit crop pests. The olfaction of parasitoids is crucial to searching for host pests in fruit crops. In this study, we sequenced and analyzed the antennal and abdominal transcriptomes of *A. japonicus* to better understand the olfactory mechanisms in this species. A total of 201 putative olfactory receptor genes were identified, including 184 odorant receptors (ORs) and 17 ionotropic receptors (IRs). Then, we assayed the tissue-specific and sex-biased expression profiles of those genes based on the transcriptional levels. In total, 165 ORs and 15 IRs had upregulated expression in the antennae. The expression levels of 133 ORs, including odorant receptor co-receptor (AjapORco), and 10 IRs, including AjapIR8a, were significantly different between the female and male antennae. Our results provide valuable information for further studies on the molecular mechanisms of the olfactory system in *A. japonicus*.

## 1. Introduction

*Anastatus japonicus* Ashmead (Hymenoptera: Eupelmidae), an important egg parasitoid for biological control, has a wide range of hosts, with over 15 host species in two families of Hemiptera (Alydidae and Pentatomidae) and 5 families of Lepidoptera (Lasiocampidae, Lymantriidae, Notodontidae, Papilionidae, and Saturniidae) [1]. *Halyomorpha halys* (Stål) (Hemiptera: Pentatomidae) feeds on many fruit crops and causes serious economic losses worldwide [2,3,4]. Releases of *A. japonicus* against *H. halys* have been used in an organic kiwifruit orchard with satisfactory effects [5]. Similarly, in recent years, *Caligula japonica* (Moore) (Lepidoptera: Saturniidae) is a significant pest of walnut, chestnut, plum, apple, pear, and persimmon fruit trees, and its egg parasitoids also include *A. japonicus* [6]. *Anastatus japonicus* reared on *Antheraea pernyi* (Guérin-Méneville) (Lepidoptera: Saturniidae) eggs had a high biological control potential against *Caligula japonica* [7,8]. Therefore, *A. japonicus* has become an important biological control factor for fruit crop pests; more importantly, *A. japonicus* can be raised on *A. pernyi* eggs on a large scale indoors [8,9]. The ability of parasitoid wasps to search for hosts in complex chemical surroundings greatly affects their success in suppressing pest populations, thus affecting the economic value of fruit crops [10]. Therefore, the ability of *A. japonicus* to search for crop pests is the key to the efficiency of pest control in the field.

Parasitoid wasps are a major component of Hymenoptera and have considerable ecological effects on the populations of many other insects [11,12,13]. They use various physical and environmental cues to search for hosts in a multistage process, including host habitat location, host location, and host acceptance [10,14]. Among these key parasitoid behaviors, olfaction is critical [15,16,17]. Olfaction in parasitoids, as in other insects, is mediated via major peripheral olfactory proteins comprising odorant receptors (ORs), ionotropic receptors (IRs), sensory membrane proteins (SNMPs), odorant binding proteins (OBPs), and chemosensory proteins (CSPs) [15,18,19,20].

Two olfactory receptors, ORs and IRs, located on the dendrite membrane of the olfactory receptor neurons (ORNs), are activated by odorant molecules in the environment, which generate electrical signals [21,22]. These electrical signals are processed and transmitted to higher-order centers [22,23,24,25]. ORs are a type of transmembrane receptor that has seven predicted transmembrane domains [26,27]. Studies have found that ORs can be divided into conserved nonconventional OR co-receptors (Orco) and variable conventional ORx, and ORx can identify the odorant molecules and have ligand specificity [28]. Orco and ORx are generally expressed in the same OSNs, forming an ORx–Orco heterodimer-composed complex through the conserved C-terminus and forming ligand-gated ion channels, detecting odorant compounds and transducing olfactory signals to insect brains to regulate behaviors [26,28,29,30]. IRs, a variant subfamily of ionotropic glutamate receptors (iGluRs), are a highly conserved family of ligand-gated ion channels, which contain three typical transmembrane domains (TMDs). [20,21,31]. Similar to ORs, each IR needs to be co-expressed with at least one of the four identified co-receptors (IR8a, IR25a, IR76b, and IR93a) for its normal function [20,21,31]. Additionally, IRs are expressed in a combinatorial fashion in sensory neurons that respond to many distinct odors but do not express either insect ORs or GRs [21]. Therefore, IRs have been identified as a new class of olfactory receptors [32]. In addition, recent functional studies indicate that IRs have diverse functions in chemical reception and participate in the sensation of odorants, temperature, humidity, and salt. [33,34].

Studies concerning the ORs and IRs of parasitoid wasps are essential for understanding the mechanisms of the olfactory system that finds the host pests and for developing an olfactory-based integrated pest management (IPM) strategy. Previous research on *A. japonicus* focused primarily on its morphology, ecology, and raising [1,5,6,7,9,35,36,37]; little information is available on its chemosensory mechanisms. Two previous studies have reported transcriptome datasets from female *A. japonicus* antennae [38,39], but neither of these studies involved the female abdominal and male antennal transcriptomes. In particular, Peng et al. (2020) reported that *Anastatus fulloi* Sheng and Wang (Hymenoptera: Eupelmidae) appears to have been consistently misidentified as *A. japonicus* in mainland China; the extensive biological literature published under *A. japonicus* in China has thus been called into question [1]. Lately, Ye et al. identified that 104 ORs in the genome of *A. japonicus* had a highly duplicated 9-exon subfamily but did not analyze the expression profiles of ORs and other olfactory genes [40].

In our study, we sequenced the antennal and abdominal transcriptomes of *A. japonicus*. Olfactory receptor genes are expressed not only in the antennae but also in the ovipositor [41]. Therefore, the abdomen containing the ovipositor was also used because the ovipositor of *A. japonicus* is too small; ovipositor collection is extremely time-consuming and may cause RNA degradation. We identified 201 putative olfactory receptor genes, including 184 ORs and 17 IRs. Phylogenetic analysis was then performed on these important olfactory receptor genes. Then, we assayed the tissue-specific and sex-biased expression profiles of those genes based on the transcriptional level. This study provides valuable information for further studies on the molecular mechanisms of *A. japonicus* chemoperception.

## 2. Materials and Methods

### 2.1. Insect Rearing and Tissue Collection

A colony of *A. japonicus* was started using naturally laid *C. japonica* egg masses collected from walnut orchards in Kangxian (105–106° E, 33–34° N), northwestern China. They were identified based on the morphological characteristics and DNA barcoding techniques [11]. Laboratory colonies were reared in transparent mesh cages (25 × 25 × 25 cm) under conditions of 25 ± 1 °C, 70 ± 5% RH, and a 14:10 h L:D photoperiod. As previously studied, *A. pernyi* eggs were used as hosts [2].

Tissues from 2–4-day old adults were dissected under a microscope and placed in TRIzol reagent (Invitrogen, Carlsbad, CA, USA) on ice. Tissues were then stored at −80 °C until use. Antennae were obtained from 1000 adults (500 females and 500 males), and 50 abdomens were obtained from females. Three biological replicates for each tissue were conducted.

### 2.2. cDNA Library Construction and Sequencing

Total RNA from each of the nine samples was extracted using the TRIzol (Invitrogen, Carlsbad, CA, USA) method. Then, we used a 2100 Bioanalyser (Agilent Technologies, Inc., Santa Clara CA, USA) and an ND-2000 (NanoDrop Thermo Scientific, Wilmington, DE, USA) to determine the integrity and purity of the total RNA quality and quantity, respectively. Four micrograms of RNA from each sample were used for cDNA library construction. The nine libraries were sequenced in a single lane on an Illumina NovaSeq 6000 sequencer (Illumina, San Diego, CA, USA) for 2 × 150 bp paired-end reads.

### 2.3. Transcriptome Assembly and Functional Annotation

The raw reads were obtained through high-throughput sequencing and then quality controlled using SeqPrep (https://github.com/jstjohn/SeqPrep, accessed on 2 February 2023) and Sickle (https://github.com/najoshi/sickle, accessed on 2 February 2023) with default parameters. Then, clean data from all the samples were used to for de novo assembly with Trinity v2.4.0 [42]. The raw data were entered into the NCBI Sequence Read Archive with BioProject ID number PRJNA931446. In order to obtain comprehensive gene function information, we performed gene function annotations using BLAST with an e-value < 1 ×10^−5^ in some major databases, including the non-redundant protein sequence database (NR), Swiss-Prot, Encyclopedia of Genes and Genomes (KEGG), Gene Ontology (GO), eggNOG, and Pfam [43,44].

### 2.4. Identification of Olfactory Receptor Genes

To identify candidate olfactory receptor genes in *A. japonicus*, the sequences whose best-hit annotations were ORs or IRs in at least one database were retained as candidate unigenes encoding putative ORs or IRs. Then, the total identified putative ORs or IRs were checked against the NCBI Nr database via BLASTx searches (e-value < 1 × 10^−5^) manually. The open reading frames (ORFs) and the transmembrane domains (TMDs) of the candidate olfactory receptor genes were analyzed using ExPASy service (http://web.expasy.org/translate/, accessed on 2 February 2023) and Tmhmm 2.0 (http://www.cbs.dtu.dk/services/TMHMM//, accessed on 2 February 2023) with the default parameters, respectively.

### 2.5. Phylogenetic Analysis

The amino acid sequence between *A. japonicus* and other three insects was aligned using ClustalW (http://www.genome.jp/tools-bin/clustalw, accessed on 2 February 2023). The phylogenetic trees of olfactory receptors were built using the maximum-likelihood (ML) method and inferred using RAxML v8.2.11 with the default parameters [45]. The ORs ML tree was inferred using the total of 313 ORs from three Hymenoptera species: 184, 31, 70, and 28 ORs from *A. japonicus*, *Trichogramma pretiosum* Riley (Hymenoptera: Trichogrammatidae) [46], *Nasonia vitripennis* (Walker) (Hymenoptera: Pteromalidae) [47], and *Apis mellifera* Linnaeus (Hymenoptera: Apidae) [48,49], respectively. For IRs, the phylogenetic analysis was inferred using a dataset containing all 17 IRs from *A. japonicus* together with other insects including 11 from *N. vitripennis* [31], 10 from *A. mellifera* [32], and 80 from *Drosophila melanogaster* Meigen (Diptera: Drosophilidae) [21,31,32]. The trees were displayed and edited using iTOL v6 (http://itol.embl.de, accessed on 2 February 2023).

### 2.6. Expression Abundance Analysis of Olfactory Receptor Genes

For the differentially expressed gene (DEG) analysis, the Bowtie alignment method and RSEM were used to align the reads on the transcriptome and to calculate the raw read numbers and TPM (transcripts per kilobase million) expression value [50]. The DEG analysis of the two different samples, i.e., the female abdomen and antennae, was performed using the DESeq2 v1.24 package [51]. The |log_2_(FoldChange)| > 1 and adjusted *p* value < 0.01 were identified as significant for the DEG. The expression of the OR and IR genes was revealed by a heatmap using the TBtools v1.082 software package and a volcano plot using the GraphPad Prism 9 software package.

## 3. Results

### 3.1. Overview of the Anastatus japonicus Transcriptome

The transcriptomes of female and male antennae and female abdomens of *A. japonicus* were sequenced using the Illumina NovaSeq 6000 platform. In total, 424,066,006 raw reads were obtained from nine cDNA libraries. After filtering the raw data, 416,603,736 clean reads were generated, with Q20 accounting for more than 95.9% (Table 1). The de novo assembly produced 144,436 transcripts and 132,646 unigenes, with the N50 value of 1544 and 905 bp, respectively. In addition, a final transcript dataset with 17,474 coding genes was produced (Table 2). Among all the coding genes, 16,661 were successfully annotated, accounting for 95.35% of the total. The largest proportion of annotation in a single database was obtained for NR (93.00%), followed by PFAM (89.44%) and SwissProt (66.87%) (Table S1).

### 3.2. Identification of Putative Odorant Receptors

We identified 184 putative OR genes in *A. japonicus*. The sequence analysis revealed that 92 of 184 sequences were full-length putative OR genes with ORFs with five to eight predicted TMDs and an average length of 1163 bp. The OR co-receptor, named AjapOrco, was also found. Other putative ORs (AjapOR1-AjapOR183) were given names followed by a numeral in descending order of the length of their coding regions. Sequence information for the putative ORs in *A. japonicus* is listed in Table S2 and File S1.

**Figure 1:**
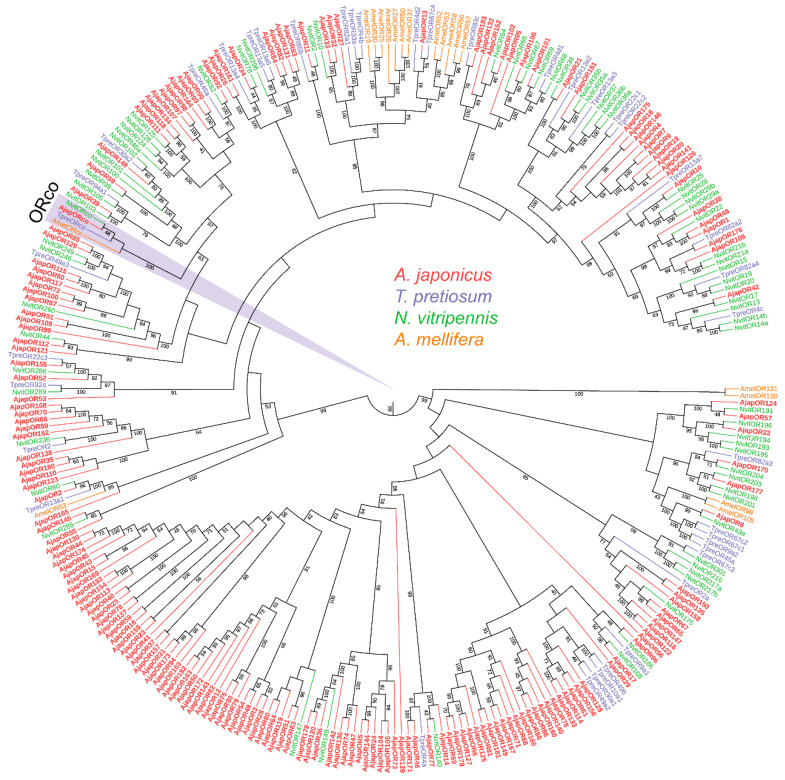
Phylogenetic tree of odorant receptors (ORs). Ajap: *Anastatus japonicus* (red); Tpre: *Trichogramma pretiosum* (purple); Nvit: *Nasonia vitripennis* (light green); Amel: *Apis mellifera* (orange). Numbers at nodes represent support values higher than 40, where 100 represents maximal support.

To further assess the relationships between the *A. japonicus* ORs and the known Hymenoptera ORs, we carried out an ML tree analysis using the putative AjapORs and ORs of *T. pretiosum*, *N. vitripennis*, and *A. mellifera* (Figure 1). The AjapORco was grouped into extremely high conservation ORco receptors. Various AjapORs were more closely related to NvitORs and TpreORs than they were to AmelORs (Figure 1).

### 3.3. Identification of Putative Ionotropic Receptors

A total of 17 putative IRs were obtained by searching the transcriptome of *A. japonicus* and annotated by BLASTx. Of these IRs, 11 sequences contained full-length ORFs, from 399 to 1004 amino acids. The remaining six sequences were incomplete due to the lack of a 5′ and/or 3′ terminus. A total of 15 IRs contained more than three TMDs as predicted by TMHMM 2.0, which was consistent with the characteristics of insect IRs. All 15 AjapIRs were named based on their orthologous relationships with the IRs from *D. melanogaster*, except for AjapIR75f.1 and AjapIR 75f.2, named based on their homology with *A. mellifera*. The sequence information of the putative IRs in *A. japonicus* is listed in Table S2 and File S1.

**Figure 2:**
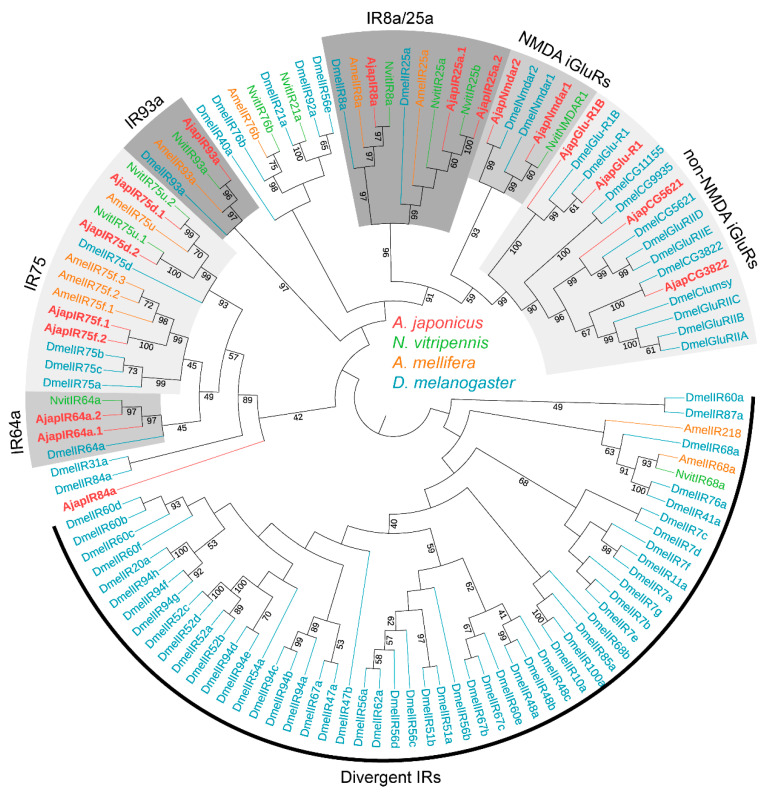
Phylogenetic tree of ionotropic receptors (IRs). Ajap: *Anastatus japonicus* (red); Nvit: *Nasonia vitripennis* (light green); Amel: *Apis mellifera* (orange); Dmel: *Drosophila melanogaster* (blue). Numbers at nodes represent support values higher than 40, where 100 represents maximal support.

To further assess the relationships between the *A. japonicus* IRs and known Hymenoptera IRs, we carried out an ML tree analysis using the putative AjapIRs and IRs of *N. vitripennis, A. mellifera*, and *D. melanogaster* (Figure 2). In the IRs ML tree, obviously, the co-receptors AjapIR8a and AjapIR25a clustered to form the IR8a and IR25a evolutionary branches, respectively. The IR8a/25a subset clustered with the N-methyl-D-aspartate (NMDA) and nonNMDA iGluRs subset. The remaining IRs clustered on different branches (i.e., IR64a, 75, and 93a) of other species. Significant separation of NMDA (N-methyl-D-aspartate) and non-NMDA iGluRs from IRs was found. Additionally, we found impressive IR duplications in the co-receptor IR25a (2 sequences), IR64a (2 sequences), and IR75 (4 sequences) subfamilies (Figure 2).

### 3.4. Transcription Profiling of the Olfactory Receptor Genes

**Figure 3:**
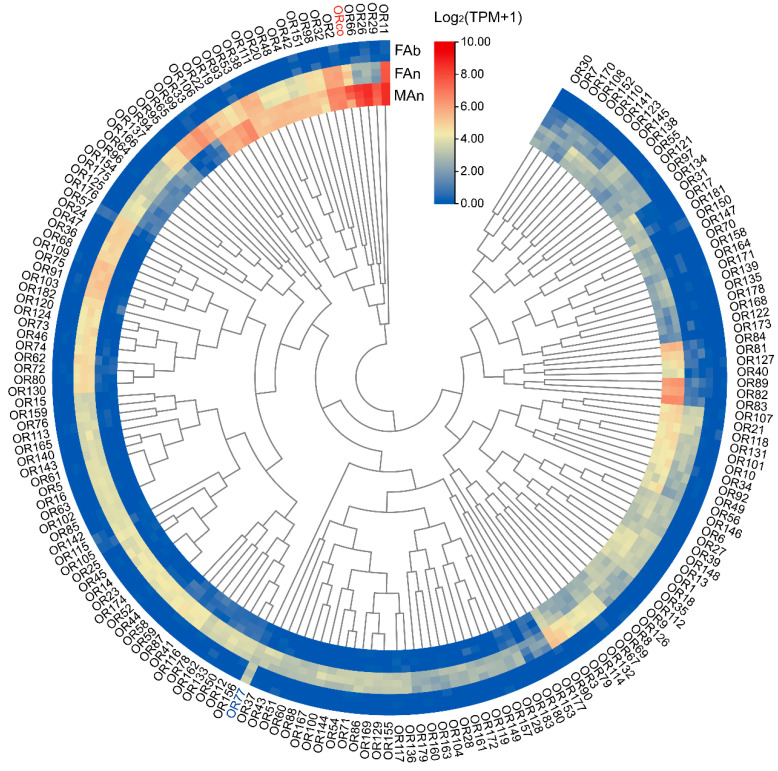
The expression profiles of AjapORs in the female abdomen, female antennae, or male antennae of *Anastatus japonicus*. The expression leves of genes were calculated based on log_2_(TPM + 1).

**Figure 4:**
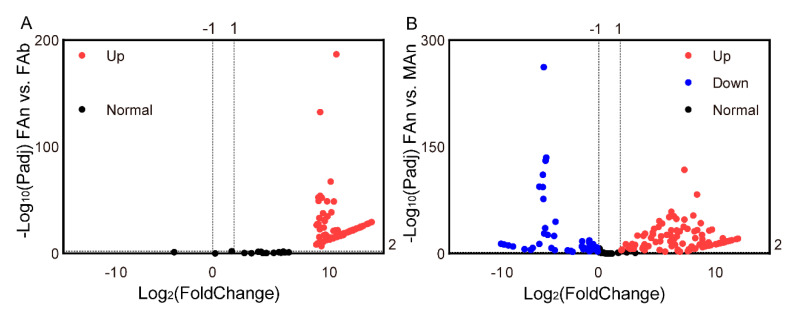
The differentially expressed genes (**A**) between the abdomen and antennae of the female and (**B**) between the female and male antennae. The adjusted *p* value < 0.01 and |log_2_(FoldChange)| > 1 were set as the significantly differential expression threshold.

The expression profiles of 184 ORs were examined in different tissues of *A. japonicus* based on the TPM values. All the OR genes were exclusively expressed in the antennae of both sexes, and almost no expression (TPM value less than 1) in the female abdomen was observed, except for AjapOR77, which was 8.92. The TPM values for these genes ranged from 0 to 206.82 in the female antennae and from 0 to 393.93 in the male antennae (Figure 3). Of these ORs, 165 ORs, except AjapOR77, had upregulated expression in the antennae (Figure 4A). Among those expressed in both female and male antennae, 104 ORs were predominantly expressed in the female, whereas 29 ORs including AjapORco (FAn: 28.37, MAn: 154.62) were predominantly expressed in the male. The remaining 51 ORs were roughly equally expressed in both female and male antennae (Figure 4B).

**Figure 5:**
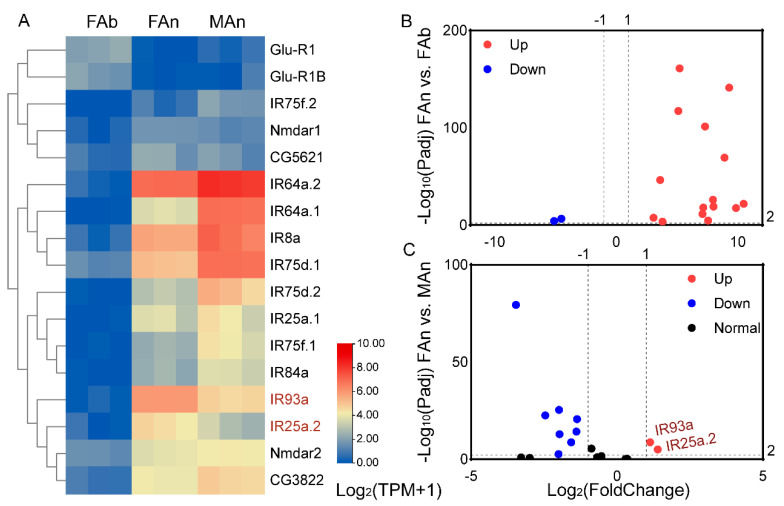
The expression profiles of the AjapIRs. (**A**) The expression levels of the AjapIRs in the female abdomen (FAb), female antennae (FAn), or male antennae (MAn) of *Anastatus japonicus*. The expression levels of the genes were calculated based on log_2_(TPM + 1). (**B**) The differentially expressed genes between the abdomen and antennae of the female (FAn vs. FAb). (**C**) The differentially expressed genes between the female and male antennae (FAn vs. MAn). The adjusted *p* value < 0.01 and |log_2_(FoldChange)| > 1 were set as the significantly differential expression threshold.

Similarly, the expression profiles of the 17 IRs were examined (Figure 5). The TPM values for these genes ranged from 0.04 to 1.84 in the female abdomen (except for AjapGlu-R1B and AjapGlu-R1, which were 2.63 and 3.42, respectively), from 0.06 to 124.45 in the female antennae, and from 0.49 to 281.66 in the male antennae (Figure 5A). Among those expressed in the female, 15 IRs genes had upregulated expression in the antennae. The AjapGlu-R1B and AjapGlu-R1 were upregulated in the abdomen (Figure 5B). Among those expressed in both the female and male antennae, AjapIR25a.2 and AjapIR93a were upregulated in the female compared to the male, while eight IRs had upregulated expression in the male compared to the female (Figure 5C).

## 4. Discussion

Although *A. japonicus* is an important biological control factor for fruit crop pests, with success in the biocontrol of Pentatomidae pests such as *H. halys* [5], the chemical ecology of this group and the molecular basis of its olfaction are still largely unknown. In this study, we reported the sequencing, assembly, and annotation of the antennal and abdominal transcriptomes in *A. japonicus,* and we identified 201 olfactory receptor genes. We also assayed the tissue-specific and sex-biased expression profiles of those genes based on transcriptome profiling using RNA sequencing (RNA-seq) data. The data provide valuable information for further studies on the molecular mechanisms of *A. japonicus* chemoperception.

We identified 184 ORs in the antennal and abdominal transcriptomes, which was more than previously reported for *Sirex noctilio* Fabricius (Hymenoptera: Symphyta) (41 ORs) [52], *T. japonicum* (51 ORs) [18], *T. pretiosum* (105 ORs) [46], and *Cotesia vestalis* (Braconidae) (Hymenoptera: Braconidae) (158 ORs) [53], and fewer than reported for *Campoletis chlorideae* Uchida (Hymenoptera: Ichneumonidae) (211 ORs) [15], *Aenasius bambawalei* Hayat (Hymenoptera: Encyrtidae) (226 ORs) [54], and *N. vitripennis* (301 ORs) [47]. The differences in the numbers of identified OR genes could be attributed to the differences in sequencing methods and depth or sample preparation between this and the other studies [55]. Similar limitations also applied in the identification of IRs from *A. japonicus* in this study. In addition, studies have shown that the number of OR genes have an association with the range width of the host [56,57]. Presumably, a large number of ORs can enhance *A. mellifera* olfactory abilities, including perception of several pheromone blends, kin recognition signals, and diverse floral odors [49]. This suggests that the remarkably large repertoire of Ors in *A. japonicus* presumably underlies their remarkable olfactory abilities, including mate and host recognition. The results of the phylogenetic analysis showed that AjapORs were more closely related to NvitORs and TpreORs than they were to AmelORs, which was consistent with the evolutionary relationship between wasps and bees [58,59]. Although the sequences of the insect ORs are highly diverse, we found various ORs from *A. japonicus* and *N. vitripennis* that had over 50.00% sequence similarities; in particular, AjapOR11/NvitOR2 and AjapOR32/NvitOR10 shared 76.72% and 75.37% sequence similarity, respectively, suggesting that they have some common and possibly identical olfactory functions. *N. vitripennis* is the most widely studied of the parasitoid wasps, and the remarkably large repertoire of Ors in *N. vitripennis* suggests that its chemical ecology is extremely complicated [47]. Presumably, the chemical ecology in *A. japonicus* is more complicated than currently understood, much as was concluded for *N. vitripennis*. In addition, AjapORco/NvitORco shared 89.26% sequence similarity, which was consistent with the fact that the amino acid sequence of ORco is highly conserved among different species [57,60].

The tissue-expressed and sex-expressed profiles of ORs correspond to their biological functions and increase our understanding of the parasitoid olfaction system at the molecular level [15,41,61,62,63]. Of the ORs, 165 ORs, except AjapOR77, were upregulated in the female antennae compared to the female abdomen, which may be involved in the antennal recognition processes for host-searching, mating, and other behaviors in *A. japonicus*. Most noteworthy, AjapOR77 was highly expressed in both the antennae and the abdomen of female, which is similar to previous reports that OR genes were expressed in some insect non-olfactory tissues, such as the ovipositor [41,64,65]. The HassOR31 had high expression in the ovipositor of *Helicoverpa assulta* (Guenée) (Lepidoptera: Noctuidae) and was tuned to Z-3-hexenyl butyrate, which helps females to determine precise egg-laying sites in host plants [41]. This suggests that AjapOR77 may play an important role in the recognition of host odorant molecules or suitable sites for oviposition. In addition, 104 ORs were predominantly expressed in the female antennae compared to the male antennae, suggesting that they may be involved in host habitat location, host location, and host acceptance. Further, the specific functions remain to be further explored. Multiple approaches have been developed for functional characterization of ORs [41].In vitro (e.g., heterologous expression in *Xenopus oocytes* with two-electrode voltage clamp system, transgenic *Drosophila* with single sensillum recording (SSR) technique, or cell line expression systems with calcium imaging) and in vivo (e.g., RNA interference (RNAi) or clustered regularly interspaced short palindromic repeats (CRISPR)) functional characterization will help demonstrate their roles in the *A. japonicus* olfactory system.

Compared with the ORs, the IRs are another type of olfactory receptor in the ORNs [31,32,53]. IRs were first reported as a novel family of insect olfactory receptors in *D. melanogaster* [21,31,32]. We identified 17 IRs in the antennal and abdominal transcriptomes. Similar to Orco, IR8a, IR25a, and IR93a are predicted to act as co-receptors in the IR group because they were co-expressed along with other IRs [21,66,67]. Interestingly, IR duplications in the co-receptor IR25a (AjapIR25a.1 and AjapIR25a.2) were discovered. A similar expression pattern was also reported in *N. vitripennis* [47]. The functions of these genes, which have been mainly studied in *D. melanogaster*, include sensing odor, taste, temperature, humidity, and salt [23,33,34,68]. Most IRs tend to be highly expressed in the antennae, and AjapIR25a.2 and AjapIR93a were more expressed in the females than in the males. This indicated that AjapIR25a.2 and AjapIR93a may play important roles in host location or male pheromone sensation. Both temperature and humidity perception in *Drosophila* are dependent on IR93a and IR25a, and these two receptors both play important roles in temperature and humidity perception. For example, IR93a, IR21a, and IR25a were co-expressed in *Drosophila* larvae, which could feel cold environments. IR93a, IR25a, and IR40a are co-expressed in *Drosophila* and can sense humidity in the environment [68]. Therefore, it can be speculated that AjapIR93a and AjapIR25a also have a similar function, that is, they can sense both temperature and humidity, and thus regulate a series of *A. japonicus* behaviors. In *D. melanogaster*, male-biased patterns of IR expression as well as functional analyses revealed that both IR52c and IR52d may determine male copulation [31]. Therefore, it is presumed that the eight AjapIRs (IR8a, 25a.1, 64a.1, 64a.2, 75d.1, 75d.2, 75f.1, and 84a) that showed male-biased expression could play a role in sexual behavior. The specific functions remain to be further explored.

## 5. Conclusions

In summary, we sequenced and annotated the olfactory receptor genes in the antennal and abdominal transcriptomes in *A. japonicus*. A total of 201 olfactory receptor genes including 184 ORs and 17 IRs were identified in the antennal and abdominal transcriptomes. We also assayed the tissue-specific and sex-biased expression profiles of those genes based on the transcriptional level. In total, 165 ORs and 15 IRs were upregulated in the antennae. The expression levels of 133 ORs including AjapORco and 10 IRs including AjapIR8a were significantly different between the female and male antennae. This suggests a range of diverse functions of insect antennae, which, to a greater degree, may facilitate the survival of insects in environments full of chemicals from hosts, host habitat, and mates. The data from the present study may also provide valuable information for further studies on the molecular mechanisms of *A. japonicus* chemoperception, to ultimately improve pest control measures by using natural enemies, which can be important in an IPM strategy.

## Figures and Tables

**Table 1:** Output statistics from the female abdomen (FAb), female antennae (FAn), or male antennae (MAn) of *Anastatus japonicus*.

Samples	Total Reads	Clean Reads	GC%	Q20%
FAb_1	44,634,698	43,922,576	35.04	96.31
FAb_2	43,084,650	41,862,678	37.68	95.99
FAb_3	46,109,628	44,842,282	35.8	96.38
FAn_1	44,678,972	44,048,062	33.66	96.16
FAn_2	55,743,476	54,930,122	32.71	96.81
FAn_3	53,767,246	52,808,490	33.14	96.74
MAn_1	45,106,298	44,630,216	33.51	96.42
MAn_2	45,411,562	44,667,556	36.15	96.68
MAn_3	45,529,476	44,891,754	33.72	96.31
All	424,066,006	416,603,736		

**Table 2:** Assembly statistics from the female abdomen (FAb), female antennae (FAn), and male antennae (MAn) of *Anastatus japonicus*.

De Novo Assembly	Total Number	Total Length (bp)	Mean Length (bp)	N50
Transcripts	144,436	111,172,508	770	1544
Unigenes	132,646	82,711,657	624	905
Coding genes	17,474	34,875,641	1996	2796

## Data Availability

Not applicable.

## Supplementary Materials

The following supporting information can be downloaded at: https://www.mdpi.com/article/10.3390/plants12040915/s1, Table S1. Summary of the functional annotations of *Anastatus japonicus* coding genes. Table S2. Sequence information of the putative odorant receptors in *Anastatus japonicus*. Table S3. Sequence information of the putative ionotropic receptors in *Anastatus japonicus*. File S1. The amino acid sequences of the odorant receptors and ionotropic receptors identified in this study.
