# Supplementary material for: Identification and Comparative Expression Profiles of Candidate Olfactory Receptors in the Transcriptomes of the Important Egg Parasitoid Wasp Anastatus japonicus Ashmead (Hymenoptera: Eupelmidae)
Source: Plants (Basel). 2023 Feb 17;12(4):915. doi: 10.3390/plants12040915 (PMC9962093; doi:10.3390/plants12040915)
Supplement: Supplementary file 1 [file plants-12-00915-s001.zip › plants-2102705-Supplementary File S1.pdf]

>AjapORCO
MMKMKQQGLVADLMPNIRVMQCVGHFMFNYYSEGKKFPHKIYCVVTLALILMQYLGMGLN
LLFEREDVDDLTANTITMLFFLHPLVKLVYFPVRSKIFYRTLAIWNNPNSHPLFAESNAR
FHALAITKMRRLLFCVGAATILTVISWTGITFMEDPVRKVVDPETNETTIIPLPKLPIRT
FYPWDAMHGAGHLFSFIYQFYYLFIAMAISNSLDVLFCSWLLFACEQLQHLKAIMKPLME
LSATLDTVVPNSGELFKAGSADHLRESQGVQPGGDNVVDVDVRGIYSNRQDFTTTFRPTA
GTTFNGGVGPNGLTKKQEMLVRSAIKYWVERHKHVVRLVTSVGDTYGVALLLHMLATTIT
LTLLAYQATKVNGVNVYAATVIGYLLYTLGQVFLFCIFGNRLIEESSSVMEAAYSCHWYD
GSEEAKTFVQIVCQQCQKAMSISGAKFFTVSLDLFASVLGAVVTYFMVLVQLK
>AjapOR1
MTVNETTGFERCMGVVRLNMNVIGIWPLDKNQNLLVRSLSYVLAFALTFLIIVPQTLKAV
IVYKDLNLMIEVLTVADISEGIALLKLFAIMYYNKDLRKLVTQISIDWQNASAKEQVIMW
KNAKLCKIVSSFCILTTTSSVIMYTFVFIITSRSKITQENSTITKSLYLKSYFPFHTEKS
PVYEIICVSQFLGAFLSCFAFSSFDGFFVCSILHFSGQLFNLKQQLKNLIYEHRSKKISF
SKTLETVIKRHLYLVSNTDIIEQDFNKVFLLQIVAVSVVLCLQGYQFVTIISEGGMKSII
NIIFIIVYSSSHLLSLFMYCYVAEILHNESQSLFWAIYEMQWYELPAKDLRLLLIVMNRL
MMPIRITVGKFANFSLNYFGSVIKTSVSYLTMLLAVKQKT
>AjapOR2
MSSKNYCFFIYLVVFFSLFLLLRSFKNGYHAPCIRIMSMDDRLEKRTWNKDATFALGLNK
FIVWPLGLWSLEKDNLFSRFRSLFSIISQLWMVYVQSMAAYLKCGTASDTVDYVMLAACA
IMALSKMIALRVHMPKIRQITSTALNDWLSVDASNIRDILIPFAKSGRTAFYVQMVSSYI
SNTLIIIGTLPFLMPPPMATNGTSWNNTSNETSSMMPSRELPLKTGCMFAESRDEIYAFL
YVYESITIMLTAHGNVGCDVFLYSLAMHICGQIELLKSNAIEIGDKSEEFSLAQEKIAQF
VERHCELLHLTDCLNTTVSGMLVVQLLLNAGLNLMLGIRVILAVNSGMPFDAVRPIVAFN
VLMLQLYLLSYASDRLSQQAESIIDAIYDSGWYELPAKFRRSLYFVLMRSSKSIYMRAGQ
FYDLNIENFKNILKASFSYFSILRVMFDAE
>AjapOR3
QNNNLHFEALKSEFYIFYSGYKYICNDIKKRKRINMDLKKIENIDESEINSVYDNEYFKL
LKTLQAMVGLWPYDTGYKSKAKRLCWYIFIIFTHLPYANGVRIYYKKDTDLFCLYVSSVV
FMGLVLFKFACTAVNMEKFKKVYKQLAINWLTLTEPEEREIMVKYANLAKLKNTVYVGAL
FIGGIGFITFPMIPIVLDFIIPLNESREKLLIICGEFQVEQEDYYLTLYIYTMMLIIIFL
TAAASIDVGYTIVTHQTLGVYSVIKHRLMKATLSYDPKSNSDYNAIVETVRLHKGALEFI
DLVDSSYSLLFLFIIGGTIIMLCVCAILGLDTFYLEMYYDTVRVCKIVITVMLHLFYLCW
PGQMLIDHNNDLFISIYATEWYNISKKGKVLLRILMMRCMRPCMFTAGGMFAMNFENFGS
IVKSSLTYVTAAVSMRDEN
>AjapOR4
MKFCDMPSKNSENFDFRNKNDNKDFQWAFGLNRLTLDFIGVWPNEINGNKSYCGYKHLRI
PFMVLWMLLGLFLPQMYALSQVIYQLPLVVDNLTTSCAAFTSSIKLFFLWNSRQVLRPII
TTVMKDWTSIEDPWEQEVMIKQAKRARIFTISGYAIMFSCFIGFITTPFFGLSIRIINNI
TDPNENRFFPLQTYYPYEATRSPYFELTYISQLIAGSFVGISFSVPDNFFGALVFHASAR
CEILGAKMNKLVSFNSNEMIKWIDDEKKFFQMKFRRMVNAHVNLIRFVDAIENSFNELIL
AQVLCLSLIICCLGFGVIKSFVTKNERPPIIQVATLSGTLLNLMIHMLVYCIASEILAMH
SLRFFHAVYFTDWYLFPGRITKNIIPIMMRSKFPLRLTAGKFFYLSLNAYLMILKNSMGY
ISMLLAVTS
>AjapOR5
GLLTERLSLNIISNIVKLYSVERNQSQRDMNQNEVEKVYNTTYFKLNKMLLIITGLWPYT
SQYEKFICRMTVYVTMSLVLLPNANAVRIWCGKDFSLCGENIVGCMFSIVIILKYTATIL
SESRITKVHKQVAKNWMSIINTDERSILIHYAKYGKIMTIIYTVYMSVAGFAFSQATILP
VILDLIIPLNESREKILIVRAEFGFDPFEYYYQLYAAYCVATVISVTVFMGIDTTYAAIV
QQNIGILSIVQHRLYGATKYRHQNKEKDFAYDAVVESILLHKEALEFVALIESSYYISFF
FIIGCSMLAISLVSVLITEYFDQTFQMIRISTGLFGVLIHCFYLTYPGQQILSHTSDIFF
HIYSCKWYFASHKAKTLLKIMMLRCMKPSIFTAGKLYVMNYENYGKIIKTTLSYMTILAS

FKD
>AjapOR6
QKFTGVWVFYNDWKKYLSWFYLYFFTFSFMISMGIKLYLEIGVNLEIVIENIVGEMYLLM
VFFKMTAGIVVRKKVKHICILIAHDWKTIIDENESKVMHKYTEYGRKVITLYSGYIIFSV
ALFLSLPIVTPLLDYIIPLENATRPKILPYYAEYGIDQQKYYYPLIGQAIFGGVGTITVL
ICIDLTFMMIVQHVVALFAIVNFRLSKVTKISEDIENCIIDFLKGNYKSYDSTIAAINLH
QRTISFRLTEMAKISKNIENCIIDFLKGNYKSYNSIIAAITLHQRTIRYVDLVEDCFSAP
LLVIFFVNMIMFSGGIAVILMKLEDRPEEAIRFIGVLIAGVIHFYYMMVPGQKILNSSAE
VFDACYGCKWYNLSKNTKTLINIMLIRSLRPCQLTGGKMFPICMDTYCNMMKTGFSIFTV
LGR
>AjapOR7
MQLTNVGISEKQSTDNKQDFQWAFGLNRLSLNLLGVWPREAGDRYYSFKIICIPLMILMM
LIALFLPQMYALSYVYRQLPLVIDNLIISGATFTSSLKLLLLWNSRQVLRPVIYTVENDW
MSIENSWEHKIMTRQAKRGRIFTISGYIITFCCCMGFITPPFFGISVRIINNITDVDYGR
YFPLQTYYPYEATISPYFELTYASQLIAATFVAISFSVPDNFFGALVFHASAQCEILEAK
MSKLLPENFNRVITPTGVEKKFVQMKLKRLVNEHVQLIKFVGAVENSFNLCILSQVFCLL
LLICCLAFGVIKSIKTDSKGLPVIQLVTLTGTLLDIMIHMFVYCFASEILAQHSLGFFYA
IYYSDWFLLPSNISRDIIIVMIRSRSELKLTAGKFFCLSFELYLKILKTSFSFISVLLAA
TG
>AjapOR8
ISSMFVINSLKKKVDGNALAKELKASIPPDEFYDNSLFKLNKKFLSTIGRWPYQDRKTRI
TIALTICIAILTVVIPEVIYFVEVFKDINKMIDCLPALILTYVAYVMMFNSVVHFHAMTE
IFDEFRKNWSDLKDEKEFEIFLQYAEEGRIFVVVYAGCVVSSLTTFSLVPLLPKILDIIL
PLNETRPITNLLEVEYFIDQDKYYWPIYFHGLQAAIGVVIIIITIDGYFILISQHVCSMF
AVLGYRIDSIGKNNTKNVYKYFKYDMGNFVSDKVNECIKYHSHCIRFAELIEDSFTISWL
MQIFVNIIIISITGVQTITRKNNPAQMLKFGLNLTSIIFRLFYISWPGQKIADHSSQVHE
FVCNVTWYDFPEKSKKLLIMMLLRSSRPCYITAGKMIPLNFETYCKLMQTSMSYLTVILS
TQ
>AjapOR9
MQSTIVGISEKQRKVNKQDFQWAFGLNRLSLNLLGVWPNEEDDPYYILKILCAPFMMLTM
LIFLFLPQIYALFCVFQELPLVIDNLISSTSNFTSMIKLFQLWNNRQVLRPVIYTLMKDW
NSIENSWEHKIMTKQAQRGRILTISGYITVTFCCMGFIIPPFFGISVRIINNITDDHDRY
FPLQTYYPYEATVSPYFELTYAVQLIAASFVTISFSVPENFFGALIFHASAQSEILAKKM
SQLLSENLNKVIPRSGVERRFIRMKLKKLVNQHVQLIEFVGALEKSFNISVLSQVICLLL
LICCLAFGAIKTIRIDNKGLPVIQLATFVSSLLYIMIQMFVYCFASEILAKHSLGFFYAI
YYSNWFLLPRDISRDLIIVMIRSRSQLKLTAGKFFYLSFELYLKILKTSFGFISVLLAAT
G
>AjapOR10
GLVKFAMDGKNGFYHAFSMCRTNLKIVGLWPESNHIERDKIFSIFCFGISITIIITFINL
VQTMELILVRNDLNKMIDNISTANLPIAVVVFKMLVFRNHKKVLETLLTNAMDDWCAKKM
KKELENMQKNAGTAYRISLICIFLGFCSVNGQATIRIGQELNIIPGQTEKRLPMVSSYFP
YNYRPSPIYEITWLFQYAGAALATLVYSGVYCLFVGLVLHLRGQVANLRLSFEDSGSNKE
QINPYQILEDKEILGMEFRKKIKRIVERHESLNRFAEGIENIFSLMFLVEILSCTIQICL
QAFLLVMLLSDNKALSILQILFMIIYVMHVGTHVFICCYAADKLREESLSICESAYNCKW
YNLPAKDAKLLLIVMQRAEKPLQVTAGKFCVFSLRLYAQILKTSGGYLSMLLAVKDRVPG
S
>AjapOR11
MSVKEVNGNSKKSYFNENYLEDTAFVVKVAKTLLKPVAIWPRDGDGSPLSNTIIYIRVGL
IFCLMLYLLVPHFSWTFFRAEDLRKLMKIIAAQVFSSLAVLKFWTLIINKNPIRYCLEVM
ENDYKTVESEEARQIMLKNAKIGRFFTTAYLGLSYGGALPYHIIMPLLAPKAPKSYDNTS
LIIPLPYPSEYFFYIPEDTPLYQITYVTQLLISTIILSTNTGVYSLIACIVMHSCCLFDV
TGHKFETLLRGRRLDKFKMDDNFTKRLSDVINFHCHAIKYAETMENALTLVMLAEMGGCT

LIICFLEYGILQDLEDSNYLGMVTYAILMTSIFVNVFILSFVGDKVKEQSELIGFQTYSI
EWYELPNDVLLNDLKFILARCNQPTRLTAGKLFDLSLQGFCDVAKTSMAYLNFLRTLDIT
>AjapOR12
INNPSQTFSKVLKSSPKKKIYKIMNHNKYVDVKMIEFIFNNNYFKLIKNLQIVVGLWPYK
NELEKKFRRCIITTLLLITFIPLVNGVRTWCGKNMDLCCENICGVLYIIVMTSKFLVTVS
CESKLKNIYQQITIKWLTIKDPEEQAIMTDYAKQGRFKAIIYFIYMIIAGIGFSQMPMIP
IFLDLVIPLNESREKVLFIRAEYIIDPFEHYYKLYTFMIIMSFIFVAVICTIDTTYTVVA
HQNFGIISVIMYRLKVATLAVNKEPDFDYNKIIEAIKLHQDVLEFIELIETSYSLLFFMV
IGLTIVGLSVCSVLAFGLNSIFELIRLVIIFIAIVTHLFYLSWPGQKLLDHGQDLFISTY
TNEWYNISQKAKVLMAIINMRCLKPNKFTAGGLYVLNFENFGSIIKTIISYMTVAVSLRE
>AjapOR13
MSVKESHYATQMNRYFLIPIGIWPIGNNASIALKILRYMAVIACYWLICFLLIPCGLHTF
LEEPNPAIKLKLIGPMSFCLMAIFKYCSLVGRTDEIGACLEHVEQDWKLYSNLGTKTELE
VMRKNAKLGRFLALLCAAFMYGGGFFYHTIMPLSSGRIATTDNTEQSLNMDIENNTESID
LEHVTVSRALTYPIYRHLLNAEQSPVYEIVFLVQCMSGFVLYTITIGACSLAAVFVVHVC
GQLEIVMSLLREFVNEDDIKRAKNNKMAEIVQRHLRALNFATRVEENLNAICFVEFIGCT
MNICFLEYYFITEWESRNTISTMTYCILLVSFTFNIFIFCYIGELLTNQCKKVGEVSYAV
NWYTLRGKKAMDLIMIIIISSSYPARITAGKMVYLSFSSFCNVIKTSATYLNLLRTVML
>AjapOR14
LRIQRNFSNYCISRCINRNLSFDNIVMDIFDSPYLIVNKKMLISLGLWPYQKMFDKIIRR
TCTAILITIFILPHVNGLYKNFGIYFDKALENISLLMFFAITVFKYVTYISAEKKFKIVY
ENIAKNWQIINDTNERQILCDYSERGRMLTIGYLMYLGGSMTLTLIAGLSPLVLNVINPL
NESRTIFSTYNGEFNVNRQKYSKEIFLFDMLTGFFTLFIFGCVDSMYVTVLEHCLGLYAI
MKTRLQLSHKYLTEKSDYVGLKCDDVIYTYFVEAAKLHAQILHFTSTVESTYSIIFLFEM
GFNMVFCCFLSAIVLLTRNNVMFCVKSFFILSCALMHLFYISWPGQKLIDHSGNLFRDTY
TNEWYLHSMKAQNLLKFMSLRCFMPCQLTAGGIYVMNFQNFATIVKTSASYMTVFASFT
>AjapOR15
ETLTFYKTNVFLFSRTMDKFEMNDKLVDDFYNNNFFKLTKQLQKFIGIWPYEVSKSVKIR
KIVVYIIITSFFIPLGNGCRIWCGVNIDICCENIMAIILFSGLFIKYLYSQIIQNKIKLL
LGIIAKNWLTLKDPKEKEIIIEYAELGRIKVFIYTVYIMITVIAFVLAPLFPVLLDYIMP
LNASRDKLLMVKAEYIIDVNQNYYEIHVYMSILSIITFFVFCGFEGCYTIIVHQNLAILS
TVKYRLQVATEAFNIQKNLDCHFIIKAILLHKESIELNSTINAWYSFVFLALNVMTVLYL
STGAVVLLSFLDEFDLEKDLFEVTRVTIIMIGVLIHLFYFIWPGQMLVDKSEDLLVSAFM
IEWYNMSFKGKQLLKILMLRLTKPNTITAFGLFEMNFENFKNIITTTISYITVMVSFR
>AjapOR16
LYFSKRIKSCSFIFFIKIMDNFDKDDKLVESVYSNKYFKNSKQSLQLTGLWPFQNKSNVS
IFFTICFIFVLNLIANARGMIKWCGYNLDLCTENMMGTLFMFGLTVKLALNFSSQDKLKV
VIRKIAQNWITLSDFEEKKIIINYAELGRIKSLGYFVYVVGTGIVFAVTPLNSGFLDLIN
PLNESRPKRHITRADYIVDSDIYFYEIHGFMLFICVATVFVTSGIDNTYNAVTHQNLAML
AIVKHRLQKATQADSIRNNREYDILIEAIQLHKESIKFTGLIESTYSLFCFIMTFVTIIF
VSTGAIIGLYMLDDQEYMALSRLITLEAGCLIHFFYLSWPGQLLLNESENLFMATYTNQW
YNLPKRSKTILRILMLKLLEPSRITAGGLLEMNFTNCGLVVKNAVSYLTVAVSFRN
>AjapOR17
VRRFVMKTNKIIDPEEVFNNEYFMMNKRLLMVTCLWPFQNRTLKIVGRILIVFAIHSMMI
PQIIRGIEEFYAENRSFEIMVENLSGFFYFQGVLTKYFTCIYFETRFQYLYEQITQDQQE
TKDKGELATLYKSWGVGRLLVLAYSTYLFVTCIVFVSVPAITPVVLNIVKPLNESRAKEL
CFYGQYYIDQQKYFFYLFVHTFFCVTVSLWISAAVDSMFLNSVYHVLGLFNIIKYRMQQI
FNFVDNLNGKVQYRNDELHQYVIKTIEIHNRSIEFATLVQSSFSECYFVCNMYVLLVLSF
GTTEVVSCLDQPIIFIRITFFAVGIVIYLFYISWPGQMLLDNSSELFISIYACGWYKLAP
KTQFLIHFMLMRSAIPCQLSAGPLLQLNFITFGLILRTAMSYFMVLLSISQERDAI
>AjapOR18
MEQQIINIKNFNWALGLNRLGLQAIGLWSRKNEIQSYSTGYKDLLIPIMVITTLLTLILP

ETYALTMVFRNLPLVVNNLALYFPIVNATSNIFILWIYRQELKPVISTLISDWMSSNASW
KRNIMLKQAGRARIFTIICYSMMFGDYIGFTITSIFGFSFRIINNITDPDENRYLPIQAY
YPYDFTKSPQFELTYAFQLISSGICVIGTTVLNNFFGALIFHACGQCEILEYKMRTFVPG
NYSIDFGTMEKENRIFRFKIQEVVKNHIHIIRFISSVEKSFNLLILVQVLFMSLTICCLG
FGVISFKNSYETLPVIQIVTLVGTVINLMIHMLVYCSACELLATHSSGICDALYYSNWYL
LSAKYSKDFIPIMTRSKYPLQLTAGKFVKLSLNCYLRILKTSFAYFSLLLAVSG
>AjapOR19
MERNKCNNRDFQWAFGLNRLSLNLIGIWPNEVDEGHYNFKVFCVPILVLIILVALFFPQM
LALSYIFRQLPLVVDNLTISCTTLTSTIKLFLLWYNRQVLRPVIKSATNDWTSINNQWEH
SIMARQAQRGRIFTISGYGIMFFCFVGFFATPLFGISIRLVNNITDPVEGRFFPLQTLYP
YEALISPYFELTYAMQLIAASFVAMSFSVPDNFFGALVFHATAQCEILEQDMRNLLPMNF
ERITQQAGGEKQFIRMKLKKLVDAHVCLIRFVSGIENSFNVLILVQVICLSLIICCLGFG
VIKSIGTDTNGLSIVQVITLVGTLLNVMIHMLVYCLASEILAGHSLGFFYAIYYSNWFLL
SSNISRDIVIIMIRSKYQLQLTAGKFCCLSLQLYLQILKSSFGYISILLAVTG
>AjapOR20
MTEAKMKENKNDNQDFQWAFGLNKFILDLMGIWPGEETNEKNSIFKLLCVPILVLIMLSL
FLPQIYALTHVYRQLPLVVDNLTMTCVTITSATKLFLLWSNQQVLKPVIKSAINDWTNIT
SPLKRKIMTRQARRGRMFTMGGYGIMSFSLVTLYTTPMFGVSVRIINNITDPANGKFFPL
QTFYPFDVTFSPYFELFYTMQLIASSFVAMSFSVPDNFFGALVFHASAQCKILEKEMKEL
LPIINAKRIIKQAGGEKSFIRVRLKQLVDTHVHLIRFVEGIENCFNVLILAQVVGLSLII
CCLGLGVIKLGLSFVQFTILMGTLFNAMIHMLIYCFASEILGNHSSEFFYAVYSTNWFTL
PSNISRDIIIVMIRSKSQLQLTAGKFFPLSFELYLKILKTAFGYVSILLAVMD
>AjapOR21
MKDFDEAGLEDLDWAIGLNRFSLRLMGVWPDSENKKSQIFNDVLRIPFMISAMFWFIVIP
QIYALTLVSSDLLLVIDNFMTSWPAFTSCLKLLFLWRGKEVLRPVVNSLVDDWLYNKQSW
EHDAMRKKAFIAKLFTIGGYIFIAVSYMGFIVAPLFGLNVRLITNVTDYGKHHLLVQSYY
PYDYSKSPNYELTHMSQVLAGTFIAMSLAIPDNYFGILVFHLSAQFEILGHKIEQFADCN
EKVSNTMKGNVFKKYLKIMVNRHVHLIRMVSTVENSFALLILTQLLCLYGMACCLGFRIL
CTFDSDSDKIPIFQIINLAAALVSLLLFTLADYSASETLATYSNGIFNCIYNSKWYVLPK
QNSRNLILMMAMSKNPQKLTAGKIVYLSMSSYCGILKSIAGYLSVLIAANS
>AjapOR22
MMPGEVETFQQSDLDIFESPYYIHSNIFLRIFGQWPYQIGVMKYFYRLFIGLVISSLFVP
NICKMIEQRHNVGIVIMGMPSFLYYVEFISKYVCFTIKQKEVESILDKIKDDFHLQKNKN
LEILHKYSEEARKFNTVYMVYVSTVVVVYSTSDWVPQILDFLIPLNESRPKSIPRLVKYN
MNAIDDNFNFIVMHGLIIDSVVAIFYLGFDTLFISCAQHACALFKIVINEAEDCLKEKQK
VEDNTKHTSKIETDEFYDRIVKVINLHKHALEFTELVDSTYTIINLLIIGIALITISLTE
FEAVIHMKDTGLMLRFSFFTVGELLHVLYCNWPGQRVMDHSLLVYTTCYTSEWYREDISN
KSKKLLILMMVRSQASAHLTAGKMYYLGSENFAKILKASMSFFTMLLSVH
>AjapOR23
TGIQKNMENNDIDDDKVESVYNNHYFRLPKQLQEIAGIWPYSSGCAKKIRCIIIFISFGS
FLIPLSNGCRIWCGVDIDLCSENASGVILVTGMFLKGLVLIIHRDKLKIMLNKIATNWYM
LSDPEERKIILEYAALGKLKSVGYFVYIASTGIMFTQGPLFPVFLDLIKPLNESREKIQI
LNAEYIIDPIENYYKIYAYLCVYATCTVILISGIDGFYTVIVHQYLAILAIVKYRLQIAT
KSCNVQINRDYNIIVDAILLHQESLLFNEIINATYSTIFLVANIFTIMYMCVVAIVIVNV
LEDFEIGKDLFELVRMNMFLIGALIHLLYLTWPGQSILSQSEDLLVSAYANEWYNISQRS
KKLLKILMLRLAKPSVVTAGGISVMNFESYGAILKSAASYLTVLLSFRN
>AjapOR24
ITVELCEIRIKEEMNQNEVEEVYNNEYFKINEVLLIATGLWPYRPRNEKLLFRITFSIIM
SLVLFPNVNAVRIWCGIDFCLCGENIVACLYSIAVLSKYYSTIFSESRILKIHEQVAKNW
ISITNEDEKSILINYAKLGKIMSIVYIVYMSIAGLGFCQVAILPIVLDLILPLNESRPKI
LIVRGEYYFDPFKYYYQLYTGYFIAMVVSLAILMSIDTTYSAVVHQNLGILSIVKRRLHL
ATKYYGESENKDVAYDTVIEAISLHKEALKFVDLIESSYYITLLLIMGITILSLGLGGVI

IAQYIGRNAFEAARMSMMAIGVLIHTFFLSYPGQLILDHTTDIFIHIYTSEWYSISHKAK
KLLKIMMLRCMKISVLTAGKLYVMNYENYGNILKTSLSYVTVLASFQD
>AjapOR25
KIKKIMDDIKLDEKLINSIQDNKFFRLPKQLQQVIGLWPFENTFSDKARQTVVYVILVTF
YIPLGNGCRVWCGVNIDICCENLLGLIYLTGVLMKLTFTNVFYNKLQTVFKILTKNWLTL
TDPAERKIILKYAELGRLKSFGYFLYMISTAVAFLIAPMFPVVLDIVIPLNESRKKMLPV
RAEYIIDHDQYYYRIYAHTIITTALNVFIFAGIDGTFTVIAHQNFAMLAIVKHRLQIVTQ
TSIIEKKQDCFMLVKAVLLHKESLEFNDVINASYEIFFLGLNFFTVVFISIASVVALRVF
DDFTLERDIFELIRLVFLLIASLVHLFYFTWPGQILLDHSYDLFISAYAIKWYNISAEGK
KLLRIMMLRLTRFSTTTAFGLFEMNFENYGLIVKTALSYITVAASYRN
>AjapOR26
MDLTEPEKENKRIEEAMKIYNKVIHSNRITLLLIGIWPLDPNASTIRRTFNRLHVSIIVT
AHFFLMVPQWFDMYELRGDLKSTAETLNTNIFVFPSAFKFINFLFMAREFKKILETMQSN
WMEIMKLPDTKENQENKDILIDWTTKASNRCVQYGILLFLAGAAYRVHPLITGLGDPGPR
DRVYPFFGRYYFDRNSDMAYVFCYAGQFVSGIIVEVTHYACDTLFSVCIYHACAQLSILQ
NDLVKLGQDEKYGIEREKELIRKHQHEIKNAKNLEMVFGTASCVQIMAGCVNMCNNGFKM
IISLKERESDIGTYVFTLIFLLLQIFLYCFPGDQLVSQSQGVDTAIYQSNWYDLNKTAKI
NSGIMKIRAQKPLMVIAGKVFILSLETYMKIVKTSLSSLSVLRAMYDK
>AjapOR27
MKIGQLVETYSAMSLKSTEKSKGFEYAIQLTRILLMPLGLWPSKSLIYKRILRPMAIGFC
LFFMFFVIIPLVLFTFVAVKDLRIRLKSFGPLGFAFMSLFKYIVAIIKDEEIASCFLKMS
IDWQKLRNPTEHKIMVKNAKTGRILTIICVIFMFGGGGSYSMLRPIIKGAIVRGNDTIRL
LGFPSYFIFFDPQIRPIWDYVFTAQVICGLTRYFVTSGVYSIAIISIMHICSQVSITSLT
LKNLPDNFDYRLLGQIIEQHLGFLKFSSKLEYIFNKICLVEIIGSTFIICLLGYYLVTDE
QKDGMMDLIAYLLLLTSFVFNIFILCYIGQILTEQCQNLGTNAYMIHWYKLSGKEAKNLL
LLVLTTQRPVFLTAGKMINLSLESFTDVMKASASYLNMLRQVTASGS
>AjapOR28
ESQDIFKNMKNIDINIDEIERVYDDYHFNLIKKLQLLVGLWPYKSKSKVRRNLICIALFI
VPFPFLNGMRVWCGKNIDLCCENLSAYLFMVVTLLKYTITAASEHKFKKLYEQVAINWLT
LTDPEERKILINYAKLARIKALVYYGYVFIGAVSFSQLPVIPLILDLIIPLNETRQRILI
IKAEYTFFDPLEYYVHLWVWFTLATAVTFFSIVSVDITYTIITHQNLGIFAIVRHRLLKA
TMFYDPKSETDYKMIIEAINLHKDVLEFIKLIETSYSILFLIVIVCTVIFVSSGAIVGIS
SIYNNIVTDAIRIAVAIFGIMVHFFYLSWPGQMFINHSDDLFVSIYANEWYNISRKGKKL
LQILMLKCIKPSTITAGGIVTMNFESFGSLVKTVGTYVTAAISFREE
>AjapOR29
MAEKGLDKNEVISNKYWIQCNYQLKIAGLWPYQKQHINIFIRIILMFGIFSMVAPMITRG
ILEIGKSLVIVLENICGFFYFMAAFSKYFFNIVSISKLQYLYEEITRDWNKIKDQKERNI
LKKFANEGRWITNFYSVYVYLAGLIFPMVQVILPAIADFIIPLNETRPKRLCFYPEYIFI
DHEKHFYPVLLHSSFGIMCTVIIFSSVDGVYICAVQHSIAHFYITDYQLHQAVILMEENA
HRKLEEIENITYKCVITSIRSHLKSLQFVELMQTAYSVPLFLNTTCILIGFITGIPILLI
TNDDFPNFFRICIIFVAAVITLFFINWPGQKIYDSSSYIYTATYLCGWYKLPIKTQKLIK
FMMLRASVPSQLAAGPLMFMNFRTFGEILKTSASYVTAMLAMEIHY
>AjapOR30
CKRKCIRIKDKMNENIDDDTVNEVYNIKYFKLVKNLQSLVGLWPYNAGVQRIFRQCIMYI
FIIAANIPLVNAVRIWYGKNIDRVCENAIGVLYLMLIFLKLSITRLRESELKNVYRQVAI
NWLTLTDPEEQKIMIKYAEMGRIRAYGYFVYMCITGIVFSQAPMIPIILDIIVPLNQSRG
KVLFIKGEFIIDPFEHYYKLYVFFMGTAITTLAVLTSVDTTFTVVAHQNLGIFSVVMHRL
KMATLNFDPKTDFDYKKIVQAVHLHKEALKFIELMKSSYSFLFLFVIGSSILFFSFSAVV
ALSLNTMFDQIRLITINIGAIIHLFYLSWPGQMLINHGQELFVSIYSNEWYNISQKAKKL
LHILMLRSMKPCNFTAVGLYVMNFENFGSIIRTTLSYITVAVSLR
>AjapOR31
MDKLDISDKEVDQVYDEASFIFTKRLQQYLGVWPYDSGWIIIVKNVTVFIFFISFLPIPI

ISAVKIYCGEDANLCCENLCAIVYFFSLALKYSFSVVSKKKYKIVLKIVAKNYVRLKAIP
EENKIIVKYVELGKSKAFAYFLYMLITAIVFTQSPLIPKYLDMINPLNESRPIVLPVKSE
YFVDMQKYYWVIYGFFICISIISLILLSGIDGAYTQMMHQNLAMISIVKYRLHMATKPST
IDQNQDYKVLIDAIKLHKDCIAFSDLLEESYSTFFFMAIILTVAFTSLGLGIVLDYLGNL
QLHLIHEILRTSMVCFAMIIHMFYLAWNGQLLMDATQDLLNTAYSTEWYYLSKKSKKLLH
IAMMKFNKPNVITAGGITVFNFQNYATFVKTTCSYIMIAFTMRDK
>AjapOR32
MLKENKSNGSVAIEEMKNAENFEYTVQMTRWLLQPLGIWPLKSTTYPSILRLLSIIICAF
LLAFLLIPGCLHLMLIEKDFRIRLKMIGPMSFCVMSIFNYFGVLFKKSKISDCIIDMASD
WHDLENFDQQRLMLEKAKTARFFTTICALFMYGGGLPYSTILPLTKGVTIIGNISYRSLA
YQSYFIFFNPHIRPIYDLVFTAHCLCGFVMYSVTCGVCSFAILCIMHICGQCNIISDTLR
NLKEKMNEKSIEKIVNQHLRSLKFASNLESILNDMCLVEMVGCTFNICMLGYYFITEFEQ
SETIGPITFFLLLLSLTFNIFIFCYIGDLLTEQCELIGDVAYTVNWYQLSGKEARNIILI
VASTQRPVVLTAGKIVTLSIGSFCNVIKASFTYLNMLRTLTANES
>AjapOR33
MLKENKLIAPVAIEKMKNADNFEYTVQMTRWLLQPLGVWPLKSTTYPSILRLLSILMSAF
LLAFILIPGFLHLILIEKNFRTRVMMIGPITFTIQNTFKYFGVLFKKSKFKGFIIDMASD
WHGLENSDQEWIMLKKAKTARIFTTICALFMYGGGLPYSTILPLTRGVTIVGNISYRHLA
YQSYLIFFDPHMRPMYDLVFTLHCLSGFVMYSLTCGLCSFAIICIMHICGQCIIISDMLR
NIKEKMNEKSIEKIVNQHLRALKFASNLENILNDLCLVEIFGSTFNICMLGYYFITEFEN
SEAIGLITYFFLFASLTFNVFIFCYIGDLLTEQCRLLGDVAYAINWYQLSGKEARNIILI
VISTQRPVVLTAGKIITLSVGNFCNIMKASFTYLNMLRTLTANES
>AjapOR34
MTLHQKEKDVDFTFDDFTFLNRWGLTFLGLWKNKSKGWRGNFLHRVHITILFTLLMLLLI
PQWLDIYVFWGNIDANAETFVLNVFTITALLKLWCFLGANDIFQEVVSTMEKNWSDTMTA
KGPEKEEHKKILLNVAGKARAYTKRYGILMYSTATMYFVSPFVGMQQDNLRIRKYPFFGW
YYFDRFSNSYYALCYASQVLIGIVVGTSNYAMDSIFLVAIYHTCAQLQILQHDLKKLGEK
EDDYDSTDQVVKLICRHQQKIRDSRKLEAAFNGSSMQQLLVSCIIICVIGFKLIIALNEG
GFEFLIYIAFMFVALLQIFLYCRPGDELISQSTAIGHAVYQSMWTSLGADLVCKLSMIIA
RSQNPLKMTAGKFYVLSLPNFTMILKTSMSFLSLLRAMYSKTDD
>AjapOR35
MSIPLALDDFLWPNRYLLTSLGFWPLESKVTIIHRFFAYFRIFLTITFAYTLFIPQIITM
YIYWGDISVLTEVGCISIAVIQLLFKLLYMLFYRRKFVDIYRSAKELWPIIHSKEEEEYF
VKLPIIAKKFTIAFFLSGMSNIITFTTASIAVWINNYLHSNGTFYNRHFPLDLSYGIDVQ
KTPRFEIIFIYQTLSMIFGVTGVTGFDSTMMTLILHICSQFRLLNIRIQEIGNKMYIKSH
NDVVVRSVTEIGQHIEHHRRTIRIAKEMKDLLSPVIFVQLMTSGIEICLSGYALMSGAEG
RKRSEISQFTIYLITIFLQLIIWCWPGELLINESTNIGDAICYHLPWRMLPATQQRDLSF
VIFRSQKECQVTALGFQVMSLRKLTEVFNTAASYFALLRSFNNQ
>AjapOR36
IHKIILQFETYENLWKINMDTEKIESTFYNSDLKINMILNMYLGVWPFKPFSDRIFRYFV
VTVACVTLSLPHIVAIYYQCGRNFLLCSENISMIVYMSTVTTEFVTPIIYRKKFEILFRM
IVDNWMIITDLEEKFIINSHSRLGKLKTMFYVCYLILAGGVFTQIVMAPVILDIIFPLNQ
SRERILILNGEYPFDPFEHYFELFVAYVTLGVVPTVILMSADSTYTAIIEQSLAIMAIVQ
YRLEKASKKQNSYDVIVSAIQLHQRGLELFEFIESIYTISFIPLIIGTVVFLSVGSIMLV
KSLDNVLELLRMSLLGIGAMVHMFYISWPGQLVIDSSEALFLSGYASKWYHISEKSKKLL
KIIMLRCFKPPFITAGGFYIINFENFGAIVKTTASYVTVALSMY
>AjapOR37
EMNDTNIDETTVDNVYYNQYFKLSKQLQRAIGLWPYQTGCSATIRYAIVYFILIVFLIPV
VNGCRVWCGVNLDICCENLIGIIYVLELFIKCAVPLVCRDNIKLLYKTVAKNWLNLIDPE
ERKIILKYATLGKMKSVSYFVYMLCCAIVVSQAPLLPVFLDLIKPLNKSREKILFVKAEY
IIDPFEHYYKIYVCFCILCLLSLFLFGGIDGFYTLITHQNLALFSIVKYRLELATSPFSV
ANNHDYKLLVKAISLHKESIELTNLLEISYSAFFLLTNLVTVSFISCAAIVTVRILDGFE

VEEDLFEFVRITMLEFGVLIHLFYLTWPGQLFLDHSEDLFVSAYTNEWYNISKRGKTLLK
IMMLRFTKPSALTAAGLCPMNFENYGAIIKTAMSYITVAISFRN
>AjapOR38
MKTNPGLSGFNKCIGINRTIMNVVGVWPSNDEKDNGLTKYYVTISILILFYPILPQARKA
YLIHNDLNELIEILSTLLITLIISIMKLLGLWYNKLDLKTIINNIFEDWNSATMHEQKIM
WKFGNFSRLIATLSITANLSICFLLCVRSYVDFLIQNHDLPSNATILRPLYLKSDFLFDI
QNSPTYEIIYITQFITAVVCAFAHSSFDGFFLLVVMHYSGQLHNLHSCVENLIENQILKK
CSFMEVFVPVINRHRHVCKNIDIIEKSFNNVFLGQIFLLMIVMCLQGYQFVTTNLNNGVG
TLPNLVFMIIYIIASIVSLLMLCYTAEQLRIQSEKLFLSIFKMKWYELPPQESRLLLMMM
RRTEKPISITVGKFADLSLEYFSSVLKTSGGYVSVLLAMKDKL
>AjapOR39
MIFNNGVIKMIKSECLEYRALPLQFYIFTFSGVWCPNDWKSLLKLLYHFYTAILVVAGVV
FWLTMLINIIVTNTESELFYQNLFAISTLTYAMYKEFFVLRKRKEIIDILKLTFDDGWYQ
PQNDFEIEVTEKYEYETRWITQLYAVGIVAGLATKAITPMIKSNEAWVLPIEAWYPYNVN
NLINYLLTYVQQLIGGVPLICLHISVDTLFVGLVLQMCIQLELLKYRLENFMITSDENVQ
QEKTLLIDNMKVDDKIANFAHKHDCLLRTGNQINTDFRGIMTGQVMITIPNICINVYLLS
QHRGGITMNLVDSFLCFATCLMQIFLYCWYGNKIINCSMDISSGVYDMNWPFLDVSARKK
LLTIMIRTTRSIQFATGTFIMNINSFIEIIRTSYSAFRVLQRT
>AjapOR40
MDKISIDEKLINSIQDNKFFKLPKQLQQIIGLWPYENTYSDKVRQAATYFILVTFYIPLG
NGCRVWCGVNMDLCCENLLGLIYLTAVLMKLTFTKVFYNKLQAVFKILTKNWLTLTDPAE
RNIILKYAELGRLKSFGYFLYMILTGVTFLMAPMFPVFLDLINPLNESRKKMLAVRAEYI
IDHDENFYRIYLHTIITTLLNVFIFAGIDGTFTVIAHQNFAMLATVKHRLQMVTKASTIE
KKQDCFMLVKAIILHKESLEFNDVINSSYEIFFLGLNFFTVVFLSISAVVALRITDDFKI
EDDIFELIRLIFLLIASLIHLFFFTWPGQLLLDHSHDLFISAYAIKWYNISSKGKKLLRI
MLLRLTRFSATTAFGLFEMNFENYGLIIKTALSYITAVVSFRN
>AjapOR41
DKEMITIDDSLIENVYSNSYFKLPKQFQHYVGLWPYETGNGKKYRFKAMCLFLSIALIPY
INGCRIWCGSDIDICCDNIAGLIYTMLILIKCLVTHSYENKMKILIKKVTKNWVTITDSE
EREIILEYAALGKSKIFAYFVYLVCTALAYSQAPLMPVLLDFIKPLNKSRDKILIKKAEF
IIDPFENYYKIYIFYCILCTLSVVLINGIDGSYTAVVHQNLAIFSIVKHRLGVATEISNI
ENNRDYEIIIKAILLHKECMEFRDLINSSYSLFFLLTNGFTILYLNVVTITFLRILDGFK
IEEDLSELIRVAMMGFGTVLHMFYIAWPGQLLLDHSEDLFTASYTNEWYNMSLRGKKLLK
QMMLRFSKPSTLTAGFIVLNFESFAIVIKTTLSYVTAVISFRQ
>AjapOR42
MNNKEGYNVAVGISRIILRLLGIWPDKKRKKNWLSDVHCFILVFIMFYFVNVPQTLLVAR
VWSDWNAVLDLLTTSDISVGISLFKMLGLWYNRNVLSQLVISISKDWSSHKNSMELQIMW
KNAKLSRLISITIISLAEGTIIAFAMIMYFSPDRSKKYKNITMGNKPLYFNGVYFYNVQK
SPLYEITWLFQCFSTIIAASAFSSIDALFAVFVLHICSQLRNLTNWLRSLPNKAINEGNF
SKMLSDIVLKHQYLNGFADAIENTFNVMFLVQMIVSSIVLCLQGYQLVFITTDGNSIPLL
ELIFMIYYTSCFAFSLFVYCYVAELLQTESMEVGNAAYESDWYNLPCLQRKLLILTMIRA
KRPFRITAGKYAAFSLELYCSILKSSGGYLSMLLAMKDRLAN
>AjapOR43
MEKFDINDKFVDDFCNNHFFKLVKQAQKAIGIWPFEVNKLDRIKQAVIYILVSSFFIPLV
NGCRIWCGVDLDICCENIMAIIFFFGVFFKYFISQVTRNEIKHVFGLIAKNWLALTDPDE
REVIFEYAELGRIKVLVYCVYLLFALVAFILAPLFPVFMDFIMPLNTSRQKFMMIKAEYI
IDVNENYFKIHTYMSILGIITVFVYTSFEGCYTIILHQNLAILATVKYRLQIATKAFNVQ
KNLDFRMIIKAILLHKESIELNDTINSSYECTLLILVITTVMYLSASAVVALSFLEEFDL
ETNLFEVVRLIILMIGVLIHVFIFIWPGQLLLDESEDLLISTYMTEWYNISIESKQLLKI
LMLRLTKPSTIDAYGLFEMNYENYKNIFKTALSYITVVISFR
>AjapOR44
MDQTKIDDKLINSFYDIYYFRLSKQFQRAIGIWPYEITSYNKIRHAVIYIFVGSIFIPLG

NACKIWCGINIDICCENIEGWIFFLAVFLKFSYTNLTQSKLKKLLELIMKNWLILTDPEE
RKIIYKYSELGRLKVFGYTVYMIATATAFVFAPLIPVLLDYIVPLNASREKLMLINSEFI
IDKNKNYYKILTYMSILVVISMFIFGAFESCYTIIVHQNLAILSIIKYRLQVAAKATIIE
KNLDYVMIKKAILLHKSCIELNDMINGWYSLTFLLINVMSVLILSTGAVVVVSRFEDFDV
EKDLFDLVRLTFVIVGVLIHLFYFTWPGQLLLNISEDLFFSAFMIDWYNISPRGKKLLKI
FVLKLIKPNTSSAYGLFIMDYQNYAAIIKTTISYITVMVSFR
>AjapOR45
MNKINSNDKLVDDICDIHYFKLAKQFQKIIGIWPYEVTILSVIKQTVVFILLSSLFIPLG
NGCRIWCGVDIDLCCENMIGIFFFFIILFKFCYTHITQNEMKTLLESVAKYWQTLTDPEE
KKIILEYSELGRIKVFYYTALVVLSCTTFVFAPLYPTFLDYMIPLNVSRQKILMVRAEFM
IDADKYYNEIYVYSTAIMIVCFFITNGFDGCYTVILHQNLALLSTAKHRLQMAGKSLNIQ
KDRDCFLISQAIILHKESTELNNTINSYYSPFFFVMNIMTVLYLSTGVIVALNRLENFDL
QNDAFELMRVVTCVLGVFVHLFYFIWPGQLLLDHSQDLFLSTYMIEWYDISMQGKKILKI
FMLRLLKPATCNAYGLFEMNYQNYAAIMKTSLSYLTVMVSFR
>AjapOR46
KSDNFLSTSNMEIYDSRYFIINKKLLSFIGLWPYDGSKKKFFIRAYYTIMITVMLIPHVI
SIMKFFHTDKPRTLENIGTIMFVSVAFLKIFTSALAEKRLKKMYDDIVKNWKTITNKEEK
DKLIEYSERGRLLTVLYISYVVSALFTYVAVPLFPIVLDKIMPLNESRPYLFILNGEYII
DRNLHYKKIYFFDFIVCCTNTIIFMSVDTMYVVCIEHCIGLFSIIKFRLRKPNKLVHNEK
SFNKQKDASYEWVIQTIRLHNRILNFTDVIEISYSAPFLVLMSLNIIYFSLVSVLIVINM
DQTLNAARFAMLLIGAAIHLFYLNWPGQKLIDSSSGLYSDAYCNEWYLASTKSKKSLKFL
MLRCAKPCTLTAGGMFTMNFENFAALMKKSVSYIAVFSSFK
>AjapOR47
MKNSQIKTSEIDLKTLEDVYNNEYFKINKILQEQTGLWPYRSRFDKTFRRLIVIFSLSIV
TLPHFNAVRIWCGKNLGLCAENISGVVYVAGVLLKYIVTVLSEDKMSKVFEQLAVNWLSI
QDNDERAIMLEYATIGKFKSVGYVGYLGIAAITFTQIGLIPTILDFIIPLNQSRQKILMV
KAEYFVDPFEYYFEIYLLYCYNAVISVTVLMSIDTTYTAVVHQNLAVFNVVKYRLQQSTK
IFEKDENYAYNKILSAIRLHKDSIEFTDLLESTYDTCFLVLICICILFLTFGAITIMENS
NSIMDLIRMTMFEIGTAIHLFYLSWPGQLVISESSELYLYTYMNEWYLISEKAKTLLKIM
MLRCLKPCCLTAAGLYIMNFENYGVIIKTTVSYITVVASFR
>AjapOR48
MDRINVNTKDIEKLYDSKYFKISKKLQLFVGLWPYHVDFKRKVQRCIVYLTLIISTLPLV
NGARVWCGKDLALCCENSLWIWYMFVVFFKYAVTIICESKMKKLHEKMTINWLTLTDSEE
KAIMTEYARKGRLKAIAYFWAMIVAGISVSQMPLVPVILDLINPLNVSRDKVLVVEAEYV
IFDPYELYYILYVLTTGVAVVSALTFISVDVNLTIIANQTLAIFSIVSRRLKLISFDPNS
KSGSDYYKIVQAIVLHKDALQFVELLESTYSAFFLILVAYSIAIPITCSVMTISSISQKN
YSSTPKTIMVMIGGVMHMFYVSWPGQMLIDHSSDVFSSIYDNEWYNISHRGKVLLNILML
RCIKPCNLTAKGLYVMNFENFGNILKSGVTYMTVAVSFLN
>AjapOR49
MTARMEKGFLECVGVIRLIMNAIGIWPLERKQNLLLRSPAYVLIFFLIFFIIIPQTFKAI
IVHNDLNLMIEVLTTANIIEGISLLKLFAMMYNSEDIKKLLMQISKDWQSTNSKQQIVMW
KNARVCKIVSFACIAGSISSAIMHTVVFFITFKANVIKENVTTIRPLFLQSYFPFYTQKT
PVYEFICVCQFIAAFFSCFAFSSFDGLFVCTILHFSGQLFNLKQKIRNLIYEHRLRKTSF
SKTLETVISRHQQLVSNTDTIEQDFNKVFLLQMVATSIILCLQGYQFVTIVSEGGIESIA
SIIFIVVYSLANLLSLFMYCYIGETIHTESQSLFFAVYEMQWYELPAKDLGLLLIIMNRF
TVPIKITVGKFADFSLEYFTSVMKTSAGYLSVLLAVKQKQ
>AjapOR50
KADKEQTLIVNNKMNDFNQIDSVFNNNYFQLIKKLQLFVGLWPYQTEFDKLFRRCIIYTL
IFVTIIPCANAVRLKCGNDLPVCCENIIGVAYLMLVTLKFLITILSESKLKNVYQQIAID
WLSFSDPTEQAIIQKYAKKGKLKAIFYFLYMVSAGVSFMNFPLIPVFLNYIMPLNESREK
ILFIKGEFIIDPYKYYYELYLFFLMDTAIVVTTICSVDTTYTVIVHQILGLFSIIMYRLK
VATSSNQPNADYNKVIESIKLHNTVLHYLKLIESIYSVLFLLVIGSTIINFSISAIIFTR

LSTFDLLRLVPVAIGYMLHLLYLSWPGQMVLNHSQDLFVAVYANEWYNVSQKTKLLLKIM
MLRCIKESEFTAGGLYVMNFQNFGSIVRTTLSYVTVALSF
>AjapOR51
VSMKKKNMELNECELNNFYDNHYFKINKKLLIYSGLWPYEISRFYQLAPQIALYLILLVA
QYPLFNGCRKWCGTDIDYCGENVAGIIYSFTIVSKLSVTYLSKNELKLIYQRLAKNWLEI
IDPEEHEILVEYARQGRKKSIGYLVFMALAGISFCQLPTLPRVLDIIFPLNESRPDIPFV
NGVYPFDRVEHFYKLYVFYLIATITSVITMTSIDTTFTVIVHQILGILEVVKYRLYLATV
SFDTFKDISYEIIIKAIRLHKEALQFIDLIQESYKFFFLTVIGNTTIFVAIGVILITSDN
SLLDLFRFMTTLFACTLHFLFLTWPGQKIMDYSNDVFTSTYTNEWYNISEKSKTLLKIIM
LRSVKPCILSAVGLYTMNLENFGIMMKSALSYVTVINSFR
>AjapOR52
ESDLFSKNLKMLNNKNKLTEASKLIIWNRRILSLLGLWPDSRSNILFAICFLYYLYQVVM
CYVTFYLSLGRLLLAVKVAIEVLTYSHVNLRILLLCKNNNIFGILFEEFKKDFEEKNFKS
EDEMKKFLDYNRLAKSLIKVLLTFPTLVSTLYYFKPILLRYLAIRAMRAANKTMTIRYLL
PYSSYSLLKMTNFQSYIIVYSSQFPLAILSGIIQSAADCLALTLGCHLCGKLAALAHRIR
NMNVKNIEDYQTVIRIHQKILRNGKMIEESLSGLLFYHLFIASILICVVIFQILIYNRPG
TRIHLITIFTLLSVNILRLYSHCWVGEYIVHESNAVHEAFYDCEWYNMSLNNRKHVIFHL
MRSQYPLRFEAGGFGTFSITTFTEVMKTATAYLSFLRNFI
>AjapOR53
MNNLSKLDINRLKQKLCTGEILFLWIKRLFTIFGVDPTNFNNFVFSSIFIYSVIHFILSC
ANLPQNLNNMNHLIDAITETGSMITVLLKFTLYRIKRQNLADLLTEVKLDYSIDKYNNEM
EIHTFIDYVLKAKAFFKVAVSVSFASAILYFLRPFSVGGFGIPIKIDSNNITTFILPYRM
NFYIPITEFNIYIGALLYCLPVIPITAFTLVADASLMLALLAHVCGQLSVLSRCIAAHFT
DDTSKFRQIIGKAIAEHHRLIRIAKSIDSIFNLLMLQQVLSMTFFLCFVGYSTLMNWSQR
ESTLMLTFLMCALGQTFLLFAYCYAGQHLIDESTYLCDALYNSAWYDLKPLDLKYYLIFM
NRAKTPLVMTGGKFYIYSLNSFLIVMKSALAYLSVLRELI
>AjapOR54
MHFADIDEKDIQNVYDNDYFKLSKQLLMFVGVWPYKLGKSKIRRFAVYLNLTISSTFVIM
GIKMWCGKDMDICCENIIGVLYLAMVFTKFQATILIESMLKEIYEYIALIWLTTENPEEK
AIMEKYAKLGYFKAKTYFVLIILTGLGFSQLPMIPIVLDIINPLNESREKILFIRAEFMV
DQFEHYYKIYFYCIISIIITVPAFTCSDVSYTVVTHQSLAMLSIVQHRIQLATQDCNKRL
NSSYNNIIQAIILHVEALKFMNLIETSYSMLLLFISGIGIISMATVAILLMGFLNDAIYS
NVARIILGMICVLIHMFYISWPGQMLIDHSEQIFLSIYTNEWYNISQKEKVLLQILMLKC
IKPCTFTAGNVYIMSFENFGTIVKTILTYITSALSFRDN
>AjapOR55
MENFKNHFEMNFKLLSCIGITIDPEIKTKHKFVEKLPVFFTNIIELLAAIADLYWIIKLW
SYDNRLIVEISTNLISDLNCMCKGFQLVTNVDTLRSLLSEMEVMWRKHEPHEKHRDDIVK
DEGKLLLFCKIYSYSAFGFFLVFGLSPITAFFMQYLSRHQDNHTYDFSQRVFYLYYPFEI
NSMSTYFSVLFYEEWILVTAIIFWHCSDLLFIQLTLSVSRQFKILNSEIEYIFSHESDER
SSERNIINFIKRYKELLRFCEVIKKIYSPIIFAVMLVSSLDICVNMFTLQKMFAEHNYSE
ASKNSFFLLALFYQAVVYCVYAEILTQQINSTSDAVYNCQWMEKNHKVKVYVKVFIMRSQ
IPFYFTGLGFFSINLSRLTWTFKTAGSFYMVLQATNEHD
>AjapOR56
MEENMNIFYSRYYRTNYYFLKLLGLWPHDNYKRNKFKRWTIVLLIISLIIPQVIRLVEEW
NRDIDIVIEVIGSLMYFLGCQIKYVSFVRVESKMKFLYGKIAEHWKSLTATEEIQTLQTF
GEMGRTLTFGYLIPVNIILVGYITLPLLPLLLDIVAPLNETRPKAFPYFAEYFIDDQKYY
FELTLHGWIVCILSVQIYGTFDTTYTQCVQHVCGLFAIVELQMKKATEIGLKNDFLDAKE
KNQQAYVHMRKAIILHQEAIQFVDLIEECYSLTYFFVVILNTAVVSLTAVDTMLNLEKGN
MKQTVRIGMLYIGFSFHLLYNMSPGQRVSDSSANIRVAAFHCSWFNVLPKTKQLVKIIML
RSMNPCQFTAGKLIILNLESFAFVFKKSLSYVTVVSSMR
>AjapOR57
MDIFKSTYYIRCNRYLSFCGQWPYQNPKIKVLNLTLLILTTSTVFGPQFAKIIELRHSFH

GIILGTPSILYYTQFLFKNVFAWLLRKEIKNVLEKIKDDFAKHKDENLKILHDYSEKAYK
INTFYTVYMLSAVVAYSSLPFTLHTLDILFPINETRLAQKPRLIRYFVNKFDDSMYFIIL
HGCIVDAIAMVFIIGFDTLYFSFAHHVCALFVIVTNKVRDAVKTANQINSEGLNKNSDKC
HDIIYREFAQAVIMHEYTLEFVYILESSFSTLNLFSIGGAMVPLTITGFEVLLNKGDFSE
MVRYVFFALGELAHIFYYNWPGQKIRDHSLLVYESCYNCEWYEGNVPDKCKKLLNLMIVR
SQRPCQLTAGKVYVLGLENFAAVIKLSMSYFTVLSSMM
>AjapOR58
MDDVDTNDKLIQNIDKDKYFKLPRRLLQFIGLWPFQTNSRIFFNIVTALPFLICLSASIK
GTTRWCGYDIDLCSENIMGGLFATSVFIKFVITLASRNKLKVVIEKITENWITLTDFQER
KIIIKFAELGRIKAFVYFVYICITGLTFALSPAFIKIINLINPLNETIPRRLILRADWLV
DEDKYFYEIHAVMCSVVVVSTLVTTGIDCMYTMIAHQNLALLALVRRRLHEATKTVNIRT
NRDYKMLVEAIRLHKESVEFTDMIETTYSLFFFFMSIVTVLMMSVGAIMTIKVLHDNDYV
TLSRIITIEIGTLIHFFYLTWPGQLLLNESENLFSATYTNEWYNLSQRGKTILRMMMLRL
TKPSRITAGGILEMNFLNYGLLIKNAASYLTVALSFQN
>AjapOR59
MMKFQDSIEYQYFPVSFTVLTLCGIWYPENWTNKKKFIYKIYTFLILLLAIVLFMEVFIK
IILIYGTDKFSLENVFAAIVFAVGFYKKINVLYFRERIIGFIFKYANNQWYKPRNDEETF
IHSNIQNKTRRITKVYASFIIISIIFRATTPILESGTFIVLPLDGWYPYNVENFMTFWLT
YIHQVVSGITLTCMHLSVDTLFVGLLLQMCCQLNILKLRLKVLKKLNNQITYKTEKVKGE
LMKDTILQRITEHESIYRFGIDLQDTFKPILMGQMVVVVPSLIINVYFLSIDTDELNLKY
LTTLFFALVSFMQIFMFCWYGNEVILGSLDVGDALYESDWIDLDEATKKICLTILTRTSK
SILITAGATIPLNVDSLAKIMRTSYSAFNLLQQTAAK
>AjapOR60
RYNVSFSLQYKALMEVELKKYYRYKRDLELLLLGPGLWPHCDKYPRSVRIFLNIIAIFST
SFMAYGVFVFCIKNAKNIVIMTRGLSLFISFITAVLKVLILMIHQNDVESLVEGVTTNFE
RHLKISEYRVHLLNHCSTFSTFFYVLNYSVLISAVMAIITPLSMLKHGKYARVFPLVLPF
SYEPGGVIHWIIFGLEVLAAFYLWTVADDSLFGYFAFNIAAELRLLSDHFEKLSSENYKK
DLKDCIDRHIELIETQHTLERVFGFLAMWITITCAICMCALVFQITAMKNFTAFSVCYII
FYVLMKLVQAYTYAWFGNIIAVESQNCLDSMYKASWTNTGDVEFMNDVLIVLSQKPFTFR
AKGIMLLKMDLFSKIINTTVSYFFLLQTLEETLDESS
>AjapOR61
MNEHEILNIKYWHDIRFALKVSGLWPYQKHRTKMFIRILFLIITYSALVPTISRGIKEIG
KNFPIVLECIVISIMILTTSIRYTATFIAELKLKYLFEQITDHWKVFTDKEEQKILHCFA
YTGYIWTRIFIVLMIYGSTLMITAKTLFPMALDIVIPKNESRQKMLCCYLDYFVDEQEYF
KYIFIHTMIFGYTVACIFVTSDTTFISIVHHSVALFNIADFQLRRAFEITKNNNKKSKSV
ENYVHNIVIKSLTVHLKSIQFVDDLQDTYSIGYFIIAIACVCCLTCLIALAMMLLGDYLN
LIRVLCCLFILLFDIIYLCYPGQKIIDASSYIFYSTYFCGWYEFPLRTKRLIYIMMMRSM
QPCKLTVGSMMDMSMVTAGMVLKMAVSYVTALLSMR
>AjapOR62
MNVDNIDINNIISVFDNKYFSFIKKLQISVGLWPYKTGYKRTIRRCIISTFLGLTLIPLG
NGVRIWCGKKIEVCVENTIGIFYIGGVFFKFLITSLSEAKLKCIYEQIAVIWLTLTDPEE
QAILREYADNGKLKAFIYLIYMAIAGVLFMQIPLIPIILDFIIPLNESREKILMVKGEFI
FDPYKNYYKLWIYFFFVCIIVITTFTSIDTTYTVITHQNLGIFSIVTHRLKMATLNFDAK
SNTDYAKTIEAIRLHKVVLQFIDLIESTYSLLFLLIIVATIIGISISSIVMINFTSIFDF
IRLFPILIGFAIHLLYLSWPGQMVLDHSHNLFISIYANHWYNVSSKTKNLLRIMMLKCMN
PSKLTAGGLYVMNFENFGSIVKTTLSYFTMAMSFQD
>AjapOR63
MNVKKDAHEIDDFFDNYYFIITKKLQIYSGFWPYESRFNKIIPRIFLYGTIIAGVTPLLL
GFRKWCILDFYLDICGENIIGISYALTAMSRYGVTAMSENKFKNIYERIAENWLKITDPK
EQRILEEYARLARIKVVVYLMFMILTGFCFCQVPMIPIMLDYIKPLNESRPKILFLRGYY
VFDPIKHFFKLYIFFVLAGVINIITMIGIDGTYTVIIHQILGILMIVKYRIHLATLYVDS
FKDVSFDVIIKAIHLHKEVLKFIDLIETTHKLLFLIEIANTIIFMSIGIVVTLEDSTFLI

LLRNLLAISGAVLHFLYVSWPGQNIIDHSIDLFLSTYTSEWYNIPEKSKTLLKIIMLRTM
KPCIFTAGGLYIMNLENFGSIIKSAVSYIAVVSSFR
>AjapOR64
MKIIMNEKEIDIVYNNDYFKLNKKLQLYAGLWPYQSNFKKRIRRTVLYTVLLLFMIPVCN
GTRIWCGVNMDLCGENITGIIYTVTILSKYWVTIFSENKLRNIYQQVAINWLRITDPKEH
AILQEYARQGRIKTIGYVVYMTVGGICFCQLPMLPIILDILMPLNESRPTVLFIKGEFIM
DPYKNFYKLYVYFMLFTFYSVLAVLSIDTTYTIVVHQILGIFSIIKYRLHLATVTFDPTE
DVSYKIIIKAIKLHKEALQFIDLIETSYSLFFFTLLGLTLLFISTSVAILLTQTIDEILD
TIRFAMIVFGVVLHLLYLSWPGQKIIDHSYELLSSTYDNEWYNLSEKSKNLLKIVMLKCL
KPCVFTAGGLYVMNMENFGSIMKTGMSYVAVVSSFR
>AjapOR65
MDIFKGRYYKASKWYLAIIGLWPYQSNFQRRIGHFAWYAVVITLILPQLLLLRKEWGKDL
SVTFENVLSIIYASGSSAKFTCASFRMPQLQILLNQIVEDWKKIDDKSEREILKKYSEEG
RILVIFYIIYMNLAWLVFNLMPFLPYILDIVFPLKNGTRPYLFSFYADYIFFDQVDYHYW
AVWHGSLVYMHSVLLFCGIDTIYVLTVKHACGLFAIIRYRLNITTKFKETNESIVEFIDQ
IRFIEKGVKKACMLHEETLQCCNLLEDSFSLCFLLVQCLCVLAISLIAVYLFHLPHDFYK
MLRIFMIFAGLLIHLLCLNWVGQQIINSSERIFNSAYFSEWYLFPLNVRKSINIIMTRSL
YPCYLTAGSISILSMESFEMVLKTSASYFTVFLSVM
>AjapOR66
MKESDKAPTKGDSKSTLEDTAKIHIVLLQLNGILPFSRDIKYQLIQLFNAIFAHFCYAFT
FSIYTWGFIKPLYEGNINLTILPQSIGAIGTQARFILLYVRRAKIFALFEFSKELWMNLN
DSEIIIARKSINKSVKLCYWYLTVCAMLVFSFVMTFTLLNQNSHSQSNETRRRYLMCPFY
TDVQLSPWYEVVIALQLITRVAFDVYIAGVDTAATFFIMIACGYLRTLNDRIDSLSKIND
LSQQRNIEKKIIDCIIFHQKIIEYADQIREIMSLFFFIQLTTTTYNISLDILAIVKEGIK
QLKYIPVFTIQLTQLFLCQWAPDKLLYESISLSESAYFVPFSGYSNKKLSRIILLLMLRA
QRPISFSAGGLLTLSMESFKKLLTSAWSFFTLLYSV
>AjapOR67
MDIFDSRYYKNCKWFLIHTGLWPFQDSFQRKMGHYAWYAINLSLTIPQIILLHKEWGKKT
HVIAENIMCIMFAVGCSAKFSTAYLRMPQLRILLLQIAKDWEKITDKSEREILRKYNEQG
RNLVIFYTFYVYMAWLLYTLMPFAPYMLDKFVPLKNGARPILLTFYADYIFFNQVDYHYW
TVWHASLVYLHCGFLFSGLDAVYVLTVKHTCGLFAVAGYRFNTSSKFEGINKSMNKINGE
IITESLFKNAIVLHNETIRCCSLLEDSFSSSFLVVQSLCVVGIAFLAAYILYFVDNLYNA
FRGLIIVGGLILHLLYINWVGQQIINSSEKILNSAYFSDWYVCSLKTRKLLDIIMIRSLY
PSYLTAGKLSTMSMESFGKILKTTLSYFTVFCSAT
>AjapOR68
MDTNIKYNSWKTIIRLEKICGLWPYESLKKNFVLRLIYFLFIISFAITLGIRMIQELDSN
AYIAIENFLALLVFSCTLLKVVLPFVHQRQREKIYNSIAAELKTIIDKEEIETVRKSAEG
GFKFAVIYLILLGGSVFIYMTLPLMKMYADYYLNDNITELAKVVPFFCEYFVDQEKYFYH
IMIHATVSAFFAFFAIVAHDLGFILAVEHVCSLLKIITIRLDKATEMTNNFERGIMAFAM
AEKAANNYVVKAINLHKLAIGDIQTIDDAYNLTWFFVLMQNMFGFGGGLVILLAKKDDIV
EIIRYSLMFMAAAVHFYVVFLPGQKVIDNSLLVFDSCYACNWYNFSKRNKILLYIMMIRS
SKRCELTGARLFVLCMESYSEMLKTGLSFFTVFAI
>AjapOR69
MQIFESHYWKLVVILQKLSGIWVYKDDWKNYVSWFYLYFFTLSFIISMGIKIYLEIGVNL
EIVVENIAGEMYLFLVFFKLNACIFLRKKMKLICALVAEDWKTIIDSNELKVMHKYADYG
RKVIAMHSGFMISSVFIFLSLPITTPLLDYIIPIENVTRPKILPYHAEYGIDQQKYYYPL
IAQAIFGGVGTIMIFVCFDVTFMMVVQHVVTLFAVVNYRLLKVSKISESIENCTVDILKG
YDAAYYCTIAAINLHQTALRFVELIESSYYLPCFVVLFVNTSMVGAGLATILLKIEDRPE
ESVRYIAVLISGLIHFYYLMLPGQKIINISTAVFDTCYASKWYNLSGKSKTLINIMMIRS
LRYCQLTGGKMFTICMDTYCNMMKTGFSIFTVLKL
>AjapOR70
MKVSNRTSIKNNSMLTLENTGVINIFLLKFAGVLPFNKDFNYHIIEVFLVLFGKFCVIFR

SSTFFWDFGSSIYKGTMDIGLTSIYLGSIGIIFRYNLLQMKSTKICAVFQCSQEIWINLN
DFERITTRNFVKKSVRLCYWYLTVCFSLVLFHLIASQLANHHSQYNETERRHLMFPFYTD
VQSTPWYELLVTIQFISHCGLDLSFAGMDTAATFFLMMACGYLKSLSHRIENLSEIDDPT
DKRIIEKKLRDCIIFHKKIIEYIDKIRETMSQILFVQLVSTTYNISFDVWVISKKGLNAL
KFIPLLTIQFTQLLLCHIAPDSLDSVNEYFSKSINFVPYSGFSNKKFNRLITILIMRAQY
PIGFSAGGFFTLSMESFKNLITSAWSFFTLLYSFN
>AjapOR71
MDKYSSHRSWTAAIILEKLCGLFPYENYMKNFSIVLIQLLVIIVSITVLGLRMIGEIGIN
PTIVVENAILLIIQICNMIKLVLCFINLNQKKKIYDQIAEELNIITDKQEIEVIKNFSEI
GLKVTILYLLLVASASIINVSLPMLRMYYNYFLSENKTRLIKQIPFYVEYYVDQEKYYYP
IMIHMGVTFGCAFVLITAHDMPYIMIVQHVCSLFKIVVIHLEKASEAIHKIEDDSTSSIL
ANKTVYNHIIKAISLHKIAIRDVICIDKVYKTTWFLVLTLNMFGFGGGLVIILLKKDDLI
EAIRYGFCFLGASVHFYIVFWPGQNIMDTSLSVFEACCNCEWYKFSKKCKTLLLIMMVRS
AKHCELTGANLFVLCMETYSEMFKTGLSFFTVFAV
>AjapOR72
KRLEQRKIFTMEAELNRYKNYSRHIKWFLVASGLWPGNHPIVKRILSFLTFFTTLSVTIT
VSKYCIHNITNIMILTKGMGIAVSFSSVVMKVCIFLLHHDDLVYLDNVLSDRYNQDLKIP
EDRTDLLNNLSAFTYSMYLDAITVVLTIFMYSIKPLIALKKYGKYVLVYPAIYPFSYKPG
GLIHWILYFIEVYGGWSLWMVTIGVDCLFGLYALQMCGELRILGKKFNDLRMDLKYNIKL
RECIQRHYLLLNAKHRLENLFGLISIWLAISGALVLCSLIFQVAEATKNNVSLVHVCHLC
CYIIPKSLQIFMYAWYGNRIADESEMCLYSVYNSHWPDSKTTKFKHDLLIVLAQEPLVLV
AKGCMQIQLDMFTKIMKASVSYFHLLRTVNDANVE
>AjapOR73
NKMDSKNIEELYNSTYFKINKVLLIFTGLWPYKSRNEKFIRRMIVSGVMFLTLLPNANAV
RIWCGVDFYLCGENIVASFYSLVIIVKYFGNILSESKIIKVHKYIAKNWATINDPDERSI
LIDYAKQGKIMSLIYLVYMVLAGIAFSQITLVPVILDWIFPLNKSRDKILIIQGEYYFDP
FEYYYELYVAYGIAAIVSVSVLISIDTAYTVVVHQNLGMFSIVKFRSHNITHDCKNNNNE
SYAEIVKTIQLHIAALQFVDLIESSYSSSFIIIFFIFVSSLSTGTALFVYYLNDFFQIVR
MSMVFMGILIHMFYLTYPGQKILDYTTETFSDVYMNEWYILPEKAKTLIKIMMLRCLKSS
VLTAGKFYALNYQNYGNVIKTTMSYATVLASFKEV
>AjapOR74
RNKSTMDSKLDTFYNNEYFKINKILQETVGLWPYNTRSHKIIRRIIVLIVLGSVGFPHMN
AVRIWCGVHLGLCAENLSGVMYALAVFSKYIVTILYENKIRQVVYEQVAETWMSIKDPQEQ
AIMIKYAKIGKLKSVGYVGYLIIAGISFSQLGIVPVIMDLIIPLNESRPKIQIVKAEFFV
DPSDYYYEIYFAYCIIASVSVTVLMAIDTTYTAVIHQNLGIFNVIKHRLKLTTQHANTDK
TDVYDSVVSVIQLHQYSLKFHDQLESTYDILFLILIIYHILFLTFGAITIMENSKIILDL
IRMSLFEVGAIIHLFYLSWPGQLVISESSDLFVYTYLNEWYNASEKAKTLLKIMMLRCIK
PCYLTAAGLSVMSFENYGAIIKTTMSYIMVLASFR
>AjapOR75
MNSDIDEKTVNEVYDIKYFKLIKKLQLQVGLWPYNVGVQRIFRQCVIYIFVFITYIPLLN
GVRIWYGKNMDLFCENSIGVLYLLLIFLKLFITRLRETELKNVYRQVAINWLTLTDPEER
KIMKEYAEKGRIRAYGYFVYMCVTGIVFSQVPMIPRILDIIMPLNQSRGKVLFIRGEFII
DPFEHYYKLYIFFMGSAISTLAVLSSVDTTFTVVAHQNLGIFSVVMHRLKVATLNFNPNS
NFDYKKIIEAVHLHKEALQFIELIESSYNFLFLVVIGGSVIFFSFSAVVALSFESMFDQV
RLFTINIGAIIHLFYLSWPGQMLINHGQELFVSVYCNEWYNISKRGKTLLSILMLRSMKQ
CHFTAVGLYVMNLENFGSIIRTTLSYITVAVSLR
>AjapOR76
KILTEKMDDRDNLVNSFYNSHFFQIPIQLLKYIGLWPYESLENDNKVRRIINGCRIWCGV
NTDICCENLMGIGYLSSLFFKIFLISVNEVKFKTMYRKVAINWSNMIDPEERDIMLKYAA
LGKIKSLGYCIYMLFTGIMFSCAPLLPVFLDLVKPLNESREKIMMVKSEYIVDHTENYFK
IYAFLTTGVVISVFTFGGVDGCYTVIMHQYVAMFAVIKHRVQDATNDLSVLKRRDYKILI
KAIILHKECIEFNQFINASYSTSFLIMNGMTIYLLSIAAIVALRVLDGFTLETDFFELMR

LLILEAGILIHLFYYTWPGQMLLDHGEDLFRSIYLSRWYNISEKGKQLLKVLMLRLTKPS
KLVAGGLYDMNFENYGAILKTTVSYMTVAVSYRN
>AjapOR77
MDIYNSRYLIINKRLLMSFGLWPYQHPVKKIALRTFLILFIFTTSLPQINGLYVNFGKDS
EKIIEHIGIIFYIYGIQLKFLTTILAEKKLKIVFENIAKNWQILKDKHEKNILTEYSERG
RILTIGYIIYMLSALFLYVTMPLIPSFLNIVKPLNESRQPGFILNGEFPMNPYDYYMKIY
FLEFLFCMCTVFIFCSIDSTYATCIEHCLGLFAIVKFRLKISTNYNECEKSVFKVKCEDM
SYDLIIRLIKLHKDTIRFTNILESSYSTSFLFLIGINMLYCSILSVLLILKRDDLLERCR
YTIILIGVMIHLFYISWPGQKIIDYSGSLFTDTYMNEWYNSSAKAKNLLRFMALRCIIPC
QLTAGGLYVMNFYNFASIIKTSVSYITVFASFT
>AjapOR78
ISLKMSVYESNYWWAPVTAQKLMGVWPYTNKSYDKLMRAFVYITLYVLIIPVGVRMIEEV
NKNPEIAIENLVGQMYLNAAVIKFTSSIIHMKKHKYIYDLIAKDWENTTDKEEFAIMERH
ARVGRKICLFYFVTCVTAGVVFVNMPAFLPLIDYLSSSTNETRPFVLPYHANYFIDQKKY
YAPLMIKVYLTGPITVAVFVTYDMAFAMCVQHVCSLFEIVKFRLKIVSMLGNDDTVSEDK
IYQQIVKTIILHQIVLENVKTLENTYNITWFFILILNTTAIGGGLLILLLKLNMPLDLLR
YGLFFGVIMLHKYFIFLPGQKIINDSLQVFQDCYFCEWYNLPLRCKKLLEMMMIRSIRSL
TLTGGKMFILSMATYASMLKAGMSYFTIFASKG
>AjapOR79
ANINMKNLKKTNSDIDEKTVNKVYDIEYFRLIKQLQLFVGLWPYEAGVDRLFRQCIVYIF
IFVTNVPLINGVRVWHAKNVGIFSENMIGILYLLLIFLKFFITSYCETKLKNVYRQVAIN
WLTLTDPEERKIMIKYAERGRIRAFGYFVYMCIAGISFSQVPMIPLILDVIFPLNESRAK
VLFIKGEFIIDPFEHYYKLYTYFVGCAICTLAVLTSVDATFTVVAHQTLGIFSVVMHRLK
LATLHFDSKPDFDYEKIVQAVHLHKDALQFIELIDSSYSFLFLVVTGGSILFFTFASLVA
LSLNTIFDLSRLAVITFGTVMHLFYLSWPGQMLIDHSHKLFVSTYLNEWYNISQKAKQLL
SVLMVRSMKTCVFTAGGLYVMNLENFGSVLKHF
>AjapOR80
MEENEINSVYDHYYFKLNKQLLIHAGLWPYESYSNKLVRRIVMYIVMSVVLIPLFNGVQT
WCGVDMDVCIENIGGVLYTTTIILKYWITSFSEKKLMNIYKLVAKNWMETTDPAEHEILV
SYAKLGRIRSIGYIIVYMIFGGISYSQSPMLPVVLDFIQPLNESRQKVLFVRGEFIIDPYK
HFYKLYIYFMAITVLTVLAVTSIDTAYTVIVYQILGIFTIIKHRLYLASVSINHLKDISY
DMIIKAILLHKEALQFVETIKAAYSLFFLVLMGLTVIFISLASAALFTSKNVLMLDYARF
GMMIFGVILHFFYLTWPGQKMIDHSLELFLSTYENEWYNISEKSKNLLKIIMLRCMKPCV
FTAGGLYVMNFENFGIIMKTAMSYIAVIASFR
>AjapOR81
MEIYKSHYWKLTIILQKSVGYWVFQDKRKSYAIWFYSYIILFSYIAILAIRLYNEIGVNA
TIVIENTIFIIYATGIIVKITATIILREKLKKLYVQLANDWKTIIDEHELKVMHKHAEIG
RKITLTYTGFIVFSIGTFSVIPLSVPLLDYIVPIENQTRLKEFPIYAEYGIDQQKYFYPL
IISFFIQGLTTIALLISFDLGYMMIVEHIIGLFTLIKYRLDKVAIISKSIENYNIDFFEG
NRIAFRYGVNAIKLHQLAIRYVDEVESCYSLCCLYFIIVNMILFGTGFAIVFMKDSLQEK
LRFTTSMIACSIHFYYLFLPGQKVINNSLEIFYSCYSCKWYNLSKKSKSLLTIMMIRSSR
PCQFTAGKMFLMCMDTYGQMVKTGYSIFTLFR
>AjapOR82
MKTKIAVSTEYDELIWPIMTTFQLIACWPNRDELKGVRNIKIVLRLFNRYLALISVIILM
IGVVLDVVIFWGEDMNRTVESCLVGSAAFLAILRIIMFNMRQNDMLLVIETMREDWMASS
IEERSLLRSKCLFSFKLAKFFISTVVIGAGTFIIMPIIQMISSEEASRELAFRGYFYSNQ
TKTPVFQYLYVLNAIVGIVASSTIAAAASFSLISSIHGAAKFAIVQQDFQSISSSSWVMS
KKLTKCVQRHQESIRFAETLEKIITYLALMQSVMSSGHLCFAAFLITTMVNDTKSLLKYC
IFIPPAIVTLFIYSYSGQCLKNESENVAEYVYSSNWFGIFSPNSIQLILMRSQKPCTITA
RKFYDMSFQSFLKVLSTSFSYFTVLLAMENK
>AjapOR83
MTSTREDSTEYDKLMWPIITSFRPTACWPNRSELEGVHNIKNVLRNLHRYFAIASMSILT

LGIVLQVVHFWGEDMNDTIECCILAATTSLIVFRILVFNSNQKDMLYIVETMREDWSRSS
VDKKLILHHKCLFAFKMAKIFFISAVVTTLAFIAMPILEMMSSGEERKKLPFDGYYYFDH
TGTPIFQFFYVANTLISAMGCSTVSAGACFVLIATVHGAAKFAIVQKDFQSISSSDWIMS
ENLAKFVQRHQECIRYAEKVEKIMNNLVLMQFVLSGGIVSCIGFQLTTMLKDKDRLIKYL
SFMPSKLMELFMYSYAGQCLKSESENVAECVYSSNWIGIFLPNSIQLIVIRAQKPCTITA
GKFYDMSFESFLKLLSTAFSYFTVLLALVDT
>AjapOR84
MKSKIEDSTEYDELIWPIMTSFQLIACWPNRDELEGIRNIKIVLRLLNRYLALISLIILM
IGIVLDIVIFWGEDMHRTVESCLVLSGCFLAIFRLLILSIRQKDMLLVIEKMREDWLRSN
IEDRSLLRNKCWFSFKLAKFFISTVVIGAATFTIMPVIEMILSEETSREFAFRGYFYFNY
TKTPTFECLYFLNAFVGAIGCSTIAAATSFSLISTIHGAAKFAIVQQNFQSINSLDWVSS
KKLSKCVQRHEESISFAETLEKIITYLALMQFVISTGLLCFAGFQMTSMVNDTKNLLKYW
TFFLPATVTLFLYSYSGQCLKTESENVAECVYSSNWFGIFLPNSIQLILIRAQKPCTITA
AKFYDMSFESFLKVLNTSFSYFTVLLAMEDK
>AjapOR85
VNSAMSIAMENEVKKYAKYKRDVKFLLVTSGVWPNFRPHPRCFRIFLSICSAFASASTFF
GIIAFCKHHGFTIQALTKGLGLLIGFFSTFIKVWILASYEKDLISLNEGLTAAFENDLKV
PELSKHLFFHFRKFSGFFYVYSYLVGLSVILLIIMPLTALKHGKYVRMFPQILPFSYEPG
GAVHWSIYGFEIYCGYYLWSVSSGSDSVFGLYALHMVGELRVLSQRFQSLKSSPSYREDM
KECVKKHLLLMKSRHTLQRVFGFLAIWLAITCAVALCALVFQATEIKNMSILRICYLMGH
CFLKVVQAYSYGWYGNIINVESELCLMSIYNAMWPGSGDLRFMSDVLIVMSQKPLIFKAK
GCMYLGLDMFSKIIHTSVSYFFLLKTLEEKS
>AjapOR86
MNIHVYENEYWKVPVLSLKVMGLWPFDNRIYNKIMPIIIYILLFTILIPIGIRLFQELNV
HTEIVIENFVGLMYLFGGVLKITMAIRCKEQHKKIFNLIAKDWSMETDEESLKTMKKHAA
LAKNISLYYGICCSSAAGLFLGIPSFYPFLDYIIPLENETRPLTLPFYAEYFIDQEKYYF
PLMAEATVAGTTVMLLFLTYDVIFTMCVQHTCSLFNILNLQLKRASVLGKNADASKGQEK
ELIIRTIYLHRRVIENVKNLEDAYNITWLVLLILNTAAIGAGLFIVLLKLNYPLELIRYG
MFFISIFAHKYYIFLPGQKIIDCSLQLSDNCYFCKWYNMSTECKVLINIMMICNIRPLKL
TGGKMFPMCMETYASMLKMATSYFAVVSGH
>AjapOR87
KITIMERELLRYKNYSREVKWFLIVPGLWPTTHPILKKFLALFSLFSMIFIGVAASNFAF
HKVNNIFIFIKGMGQSVSFYSSTFKALIFLLYEEDLIYLNEYLSMRYKRDLEDEKNRSYL
LYRVATFNYLMNTYMVSTGLTVVLYAIVPIIVLFKYDMYIRMYTIMIPFIYQPGGSMHWI
LYFIEILAGLSLWIVSIGVDSVFGLFSLNMCGELRLLAREFSNLRVSSEYRKNFKECIEK
HRLLLKVKRRLENIFGPIALWLAISGAIVLCAVIFQASESIKTKGGLATFGHMCLYMIPK
FLQIFTYAWYGNILSQESEKCVASIYYSQWPDLCEAHFKNDIIMVLTQKPIVITACGCMS
IHMDMFTNVLNTSVSYFFLLQTLSEKSEPP
>AjapOR88
MNDDYYYVYKSKYWKMQVLSLKFLGVWPFDNNLSSKIMQIIFYISFASLIIPMGIRLYEE
LNFHPEIVIENFVGEMYFAGAVTKFTMTIFYKKKHIQIFNLIARDWAQELEKEELKIMER
HAAIGRFISILYGICLSNAAGGFLIIPLVQPLFDKSKETVLPIYGEYFIDQDKYFFPLII
KITISGTMVAIVFFSYDITFAMCVQHVCSLFDIIEFNLEKISAFKEDTEAYESQVKPLII
KIIRLHQTIIEHIKNLEDAHNIVWLIVLVLNMLIIGGALFIVLIRLDDPIEMIRYAAFVN
AVFIHQFFVFLPGQKIINYSLQLSNNYYFCKWYNLSSKCKVLIHIVILRCIKPLKLTGGK
IIILSAETYLDMLKAGMSYFVMISNTQEQ
>AjapOR89
MKKDKKSFEDTSFDEATNVHVSLLKIIGLVAFKNHNMHYIPTILLSLNTHLCHSLISTMY
IYDVMSSLVDPEHDYDVQAIAEGVGLCGVHIRYFILFMGRAKIAEMIAESRQLWVYLTDD
EKDVMKNFIKKGIKLTEFYGGNCVVFLTVSLVSVLMQILKETQPRYLPYPFYYDVQQSPW
FQLSGMLQILSMVGSSISGTGIDTMGPFFTLMTCGHLRNLSTRLQNLDQLEINDGSNIRT
QIIECIVYHQKIIKFYEKIQKMMNVLFMTQLISSTYNMSLCGLKLVGKDPDKFKYLVVLM

ILLTQLFTCQWAPDILVSESENLATKAYIVPMANQGDYKKIAKLIQIMMFKSQHSLQLKA
GGFFTLSMQSFSTLITNAISFFTVLRSIN
>AjapOR90
MIEHTLPEAFCLASGWGIWEPVYWQSSLAKKFYSFYTFVNVIIIFVLLTIQLIALFLLTE
NLNDFAEASYVFLAGFNGCVKGITLIFRRKRVAAMVNELLHENCTPRNVEEMKICSHYNK
MARYTVLSYVTLVGMSSGATAILPCFLAPVKYALQLKAWYPYEITNDLIYWCTYVHQALG
ILIGAAWNATNDTIVAGFMMQTCAQLDLIICRLQQLPENTKAAFLRKVSENEIKRLEEKV
VKESVLHHLQVFQFAKDLKNTFSPSIFIQFGLSLIIICVTIYELTAEDSNIPVSFMLIFL
SSMLVELFLYCWFGNEVTLKSLEFSNAICKSDWTELRVETLKGLLIMMLRTTSPIYMNCG
PFITLSLQSYVAILKLSYSAFNVLQSSSS
>AjapOR91
EQRSKMNVMDVNLMKYNKFANLQKYMLICSGLWPDNSNPFVKITLASLSTVSSCITSLSI
LNFLIVHINNVMLAANSAALAGAFFSSALKSVALLWYSNDLVYLNISLWSKFDNQIQESR
YRALGLTHLQIIAKLYYTLMGTILLSCTFNYLIGYVRMRHGLYMLMFPSILPFDFLPGGK
IYWIMEITEILANFFVFTISTGVDSIFGLYALQLCGEFKILAHKYENLGESEDYKRELKE
CIERHNVLMTCKNKLERVFGFVIIWVVISTAIIVCMLLFQILRLTLSSFRFGFVIAYIIV
KLSQAFAYAYCGQLIVDESAACLDSIYNATWPGCGDKRFMNDILFILAQRPLQFSAKGLI
DLKMDIFSKIINTSVSYFFLLQTLDEESK
>AjapOR92
MESLNISFAILTGVGIWKPIITSSKLNENLYNLWRIIFTPLPYMLASAQLARIMFVDMDF
DELTEILFIFISILNVCCKSMSFLVRRKELANLANMLNAGITKAHDVDEADIQDQYHQFI
RYTTISSTILVEITAITFLLPPLFQPKNYRNLPFKIWLPYNYTTGNIFWITYVTESIGII
TVSLISVSSNTLIFGFLIEACHQFELLSHRFSVLPKFLQSTMKNEKMMYDIDKLERLLMI
RNIRHHIFIFNFINAFRKTFSSAIIGQFIVSSVVISMSVYQLSSMKTIDVVLFTRILYLM
CMLIEFFLYCWFGNELTLKSMKLSNSVFEMDWIALSENSNKDIIITIQRSSKPLIISSGF
FVILSVDSFMKIIKLSYSAFSVLRQASS
>AjapOR93
MINHTLPEAFFLASGLGIWEPLYWRSSLAKKFYSVYTLINVTIAFFLMIMELMALIVLTE
NLNDFAEASYIFLAGLSGCIKGATLLLRRKQVVALINELLRQNCTPQNIKEMQICSHYNK
IARYTVLLYAILVSISSGISAILPCFLAPVKYVLQVKAWHPYEITNDLIYWCTYVQHGLG
ILIQAGWNAINDTMIAGFMIQSCAQLDLISCRLQMLPENTKAAFSRNISKAQIKRLEAQV
IKESVLHHLQIFKFVNDLKYTFAPSIVVQFCLSLIIICVSIYGLTSERSNIPVSFMLMFL
SSMLAELFIYCWFGNEITIKSLEFSEAICKSDWPELQTETLKGLLIMMFRTSPIVINFGP
FINLSVDSYVGILKLSYSAFNVLQSSSS
>AjapOR94
FSLATKLQKLMGLWPYETGLIKIIKNVSIYVFLLVIMQTPMINGARIWCGVDINICCENL
TAALYLHCIAVKYSFSVMCQKKYKDVLVIVAKNYIMLKSIPEEKKIVLEYSKLGKSKTFY
YFFYMLATAIVFSQTSVVPMYLNIIYPLNESRDLLLPVKAEFIIDHKKYFWVIYVAFIGL
CVVSLFILTGIDGTFTQIMHQNLALISITKHRLHLATKDFSIKANKDYSILVSAIKLHKD
SLALTELLENSYSLFYLLSISMSICFITVATTIMFDYIETIAINLPQQLEILRASMILWG
LLIHLFYLSWNGQLLLEHSEDLLVSTTTTEWYSLPERSKKLLLIAMIRFLRPSMITAGGL
IILNFENYSALIRTTCSYIAVGLSVRGN
>AjapOR95
MTSEINVIGRPIEMSLRLIGLWPGVSNLIGRIFVWSVLISNIPFQLWDAFVNLDNKILLM
DSLSTTVATCLFFIKLNIIFINHRTITKLVNSIFEDWEITKNRTDERNILEEKTRKTYTF
CKWHFCMYMLSVTLFTCDAVFTYLVMPVSKRKFILCMHFPFDSQQSPIFELFLVINFIET
VIAAGGNAISESLIITLAIHASTRVDFMKMDLKRFSDTYMSSENKINKKISKGVECLVVQ
HRRIIDFIENIEELYSYISLCQCLCSTLIICAMGYVIITSLESEVLNTVTLVKFSVFSIL
KLMEVFCFCFAGEYLTSQTSSISNTIYECTWHDLHPSDSKMLMFLILKTQRPLTITAGKF
IDLKMESFTSILKTSASYLSVLRTVS
>AjapOR96
RANKLLLKVSGLWPYQNKYINISIRIILLMSMLTMCIPQIIRTSFEWGKNLPIVVENICG

FLYFQTSASKYITTFINLHRFEFLFKQITNDWEEFSSSGEQKIFNRYSSEGFYLIIFYAT
YVYFASTLFTILPGLVPTILDVIMPSNETHPKQLCFYGEYFIDQQEYYYQLLIHTLFGVM
STAIILSTTDTLYVACVQHSVVLFNIVDYRFQKVFDLVKSNNYNQSKDIDTEIHNCVLRC
INIHKKSIEYVVLLQNVYSTTMLITLAAAIVGLSFANILMAMTINDFPNFARILLVAIGV
FINIFYVSMPGQKIQDLSEHVLFSCYFCGWYKFPQKTQRLLRFMMLRCSKPCKFTAGPLV
EMNLQTCGAILRTVLSYAAFISSVT
>AjapOR97
MFSYSFNVITFAGTWRPLCIKSKYIKCLFNFYTAIGLVVYYTFMISEILDIFLWAENIQQ
ITENMIQLINVINASQKNLAYIVRRKKIIKFLNLLTGDLATPNTPAEKSIKKEFDNEYRS
NAIKLGVLYSISVFTFVYLPFFITDSEDRFLPYRAWRPYSLDNVNNYYIAYLHQSWAVTI
AACGNYATETLVSGLMIQICAQFEILEERFLQMPKILNKMREQGESENKVLISENYIIIR
LVQHHWLIFEMAEMLNSIFVFVIFFQFFSSITVLCVCTFNLAMMPVNGEFMTVFLFTLCM
LIQIFLYTWYGNEITLRSTDFGEKILRCDWNSLSLRGIKSLMIIYERTTKPIVLSSGYVI
ALANTSFTSIVKTSYSVFNVLSVN
>AjapOR98
MYSYSFSFLILAGTWRPLSIKSKYVKHLYNFYTAIGSTVYYTFMISEILDIFLWAENVQQ
ITENMIQLVNVINASQKHLAYILKRKKIIKFMNMFTGDLGTPNTPTEKSIKEGFDDEYRG
NAIKVGIIYSISVFTFVYLPFFITDREDRFLPYRAWRPYSLDNVNNYYIAYLHQAWAVSM
AACGNYATETLVSGFMIQICAQFEILEQRFLQMPKILNKMREQGESENKVLISENEMIIR
LVHHHWLIYEMADTLNTIFVFVIFFQFFSSISVLCVCTFNLAMMPLNIDFITIFLFTLCM
LIQVFLYTWYGNEITLRSTDFGERILRSDWNSLSRRGIRSLMIIYERSTKPIILSSGYVI
ALSNASFTSIVKTSYSVFNVLSVN
>AjapOR99
MNSSMELNKYRKYANNVRLLLIPVGLWPNEGKSLISKFLSTFAALSLVLLNINALNYCYI
HITDINRVTRSFSWCISIWTMLMKVVLFVIYRDQLIDLNESLSKTFDEELKCLHSAAILL
QRLSVFTGVYYTMVVALAGAFTLFAVIPLIFMITERKSLLVYPGKYPFNFEPFGTIYYLI
YTYEAFLAFLFFCIGCGTDTAFGFWIFQICGQIRLLTHKFSKLRPSKDYIKELRECLEKH
EMILRCRNHIQKVFGFIVIWLYVTISLVLCEYIYKISKMSIKLSFWQSVMFFTFATKFLQ
AFTYAYCGSIIDIESEKMIRAIYECHWPGTGDKRIMNDVLSLLTQKPMNLRSYNYFTISM
AMFLKIVNAAVSYFFLLQTFDAS
>AjapOR100
MEAELIRYKNYSRRVKWFLIISGLWPNVHHIIKRILSFISFFTIFVVAASSTYYCFKNAT
NIMMFTRGMGISVSFFSALMKVCIFLLHHEDLTYLDHFLSNRYNEDLKNTNNHIYLLSNL
TDFTSFTYLHLASAVVSISMYMVKPLLILRQYGKYVLVYPTMFPFFYEPGGVVYWILYYI
QVLAAWSLGTVTIGVDCVFGLYALQVCGESRILSKKFSDLRMDLENRMEFRECIERHYML
INVKHRMENLFGLITLWLAISGALVLCSLVFHVSESLKNHINYGVTCHLFMYIMVKFLQI
FMYAWYGNRIADESRACLDSIYNSHWPDSCNTNFKNDLLIVLAQEPLILVAKGCIRIQLD
MFTNIMKASVSYFHLLRAINEAE
>AjapOR101
MKIPIIDWTLELNLKLLGFWPDQFSIFGLMIMFASYTVLLPFQCWDAVGLIEKPVLLMDS
LSAIIAEFLAFLKFTIMWINRRYLLNVLREMASDFDEKDVRDKWVKLNYYSHKFSRYDFG
WYLGACLMYHVQFFQLYFLTPVEERITLLKANYPFDYKSTPNYEIIYVVQTFIGTSIAGI
NAMTEALIVALVLHICGHIELLIDKIKLFSDHCGNITDNNINVNNKSQFLIREILKRHLK
VLNLVKKIDHVYTYVSLAQMLFSTIIICLTGFVIIYALETNDTVLLAKFIMYIFVLLWQA
FSFCFAGQYLLNKSAEIPIKMYDTFWYNIEPKEIKAVLFIIKKAQIPISIKSGKLDPLSV
EGFARVVKTSFSFLSVLRATV
>AjapOR102
MENNIFIEHHKYGLRIIGAWPIKHLPGFYCAIGLLLFFLIFEIWNVTVVYADLEELMDNL
VSTIGVFSGLFKIITFRLKRRNLRRLIKKIFDDWNIDNDKLLPPAMHKNCLRSHLISKSI
ILLYNTMNFTYLLRVIISYIFDTVEDRKFLAQVTFPFNARKSPVYEIIIIGQFITASLCF
NGHALIEGLLITSVLHATSKVDIVQKEILKLSRIGQANYKNKRFILMILKNLSQKHLKFI
EFSENIQDVFSYVSFFHIFFLTFIQVVGGYMFIDALEGNAKLINLIHYAILTMSFLVSSG

YYCVAGEYLTNQSEIIFKEMYNCFWYEFPTSEKKLINFILIKARKPVNLRVGKFSNLSLT
YLTSIMKTSFSYLSLVRAIR
>AjapOR103
DKYDRLPFSVRVFDSGWKKVTMNIGVYLASSILLFPMVNGVRVWCNVDFSFCCEHVCTSI
FILAVSTKYVLIFIFKNKLKMMFRIVAKNFIKLQHDPEEKIIILNYANLGKTRAFGYFVY
MTFCATTFSQAPLVPIFLDIINPLNRSRKRLLPLRGEFIVDPHDYYWVIYAGFFWICFHC
LFLMAGVDGIFTVVMHQNLGLISIIKHRLHLATKDASVKADNDYDILVSMIHLHRNCLAL
TNLIEIAFSLFFLLTNILTVVIISIAMVVFFDFVDNYDPKQAVNIFRIFFVLFGVLIHLF
VYSWNCQLLLQQSEELFRSTYMNKWYNLSERSKTLLKIVMIRFIQPSVITAGGLTVMNFE
NYGAIIKTTLSYITVAISFK
>AjapOR104
VYNKPYFKINEILLIATGLWPYRSRNEKLILRVIVFVVMFFVLCPNANAVRIWCGVDFCL
CGENIVGCLYSIVVILKYYVSILSESKLLKVHEQVAMNWITITDKDERSILINYAKHGKF
MSIGYIVYMSSAGFAFSQATILPVILDLISPLNESRQKILIVRAEYYFNPFEYYYQLYVG
YCIATVISVGILISIDTTFSAVVHQNLGILSIVKHRLHKATNCHDENKDIAYDVIVKAIR
LHKAAIQFVDLIESSYNITILILIGCFVIALSIGMVIIMEYIGKTFEVIRFSMMAIGVLV
LMFYLSYPGQQILDHSSNIFLHIYLNEWYILSQKAKTLMQIMMLRSLKSSVLTAGKLYVL
NYQNYGNIIKTTLSYVTVLA
>AjapOR105
MDKTKVEEVYNNTYFRINEKLLIFTGLWPYRSKNEKIFCRATVFIIMILVLIPNANAVRI
WCGTDFCLCGENIVGCLYSIVVILKYYVSISSESKIVNVHEQVAMNWITITDKDERSILL
RFSKDGKLLTMIYVAYMTLAGFFFSQATILPVILDLIVPLNHSRKKMLVVRAEFGFDPFQ
YYYQLYTAYCIATVISVAVLMGIDTTYSAVVHQNLGILSIVKYRLHTATRQYDGITDDAY
KSIVKTVRLHQAALEFVDLIESSYSISSLILIGCFVIALSLGAVIIMEYMGKTFEVIRFT
MMEIGILVLMFYLTYPSQQILDHSSAIFVHSYMNQWYFISQKAKTLLHIMMMRCLKSSVL
TAGKLYVLNYENYGNVII
>AjapOR106
MSSLINNKLEICLKVVGLWPYKFNYIGPLLLLSTLITTLPFQCWNAVSLMENPILLMDSL
SDILAEVLIFIKLFAMWYNISDMKDLICTINDDWDRKILPNEWKTLAHYCNMFCLFDVCL
YMSAVVFYYPDMILAYIKKPSESRILLFQSHYPFDYHESPNYELVLLVQIIQGYAMLFVD
SLSKTLLIAFIFHVVARVHLLKGYIDEYNKSCIPTRDKEVDQSFEKLKRVINEHVHILML
VKKIDAAYNYISFFQILFSNIIICVTGFVIITAMESSNLMLMIKFVLYILAMVFQAFTFC
IVGQYLRNKGESILHMIYNCLWYNTKQKDIKAIIFIISNAQDPLTLNGGNLFELSINCFT
QILKTAASYLSVLRAVYS
>AjapOR107
MKILPITFTILICCGFWRPICYQSLKKFAYNCFSFIMCFLICTFTLSHFIDIVISASDFE
KLAESCFMLLSMLNVCCKMLNILYFRKNILELLNILMSGLCKTQDTTELRIQKKIDKKIR
FATLCYFTLTETTCMLITARSFFGTAKHILPFKVWIPYEIKSNFGYWLTFLHQTIAHVGA
ANLQIANETLICGLMLQTCSQLKILKHRLKKIPQSIKKTNKIGSSDSTKTFIMKCINHHR
SITQFSEKLNKTFKITIFVQFVISSLVLCSSVYLLSKLNLLSVQFMSLFLYLFCMLYQIF
LFCWYGNEIILESINIGRAIYFIDWTTLDTKDKKNLFTLMIFVHKPITFTSSFLISLSID
SYCKRGHNLTMCSSVSI
>AjapOR108
MYIGFHVIIFFCLYFLLFFFINIPQSKMLLIVWGDLNMILDILTTADIMIGLACVKLFGI
WYNGQDLRYLLFEMTQDWKKSKDFQQREMWKNVKMCKLILICYTILTYGTLVIYALQMLI
ILYSHMTQGNHVNTTLSKPMFVNSKFFFETQKSPIFEIIWICQFLACTIGIGAFTSFDGF
FIFSILHLCAQLTNLKIDLKSLMFQNTFKRKTFRYLIKTIIERHVHLYKFMNCIEDNFNK
VFLVQMICYSITLCLQGYQLVINLSERSDNNFATLAYFVIFTIANIMSLFMYCFVAEKLR
VESTSIFFAVYEMNWYELRPEETRMLINIMNITKMPMKITAGRFATLSFEYFTKILKTSA
GYLSMLLAVKSSLQSEI
>AjapOR109
MDANLIKYGRFANIVKWSLIFCGLHPNYRFFGTIITVFIQFATLTLTSLFILIFFFNNLN

NIKLAVSSAASFGALCTCILKSMAFLVYNDDLVYINSSLSTKFNNDIQQSKYRALALNNL
QIISKIYYVIMTWIIITGVFNFLQGYKRYKRIPSTHMLSSALPFSYEPDGIIFWIVEVIE
IFGLFYSLFVAFAADVAFGFYTLQLCGELKILSYRFENLGESKDYKQDLRDCIERHLLLI
RCHKKLEKIFGFVIIYLVLMNSVIVCSMLFQFTNVSWHSIRFRFLCFYIPGKLLQTFSYT
FCGELINEESSLCLNSIYKTTWPGCGCRTLMNDVYFISLQKPLEFSIKGFMVLKTDTFST
IVNASVSYFFLLKTIDE
>AjapOR110
MIESTSIEDFVCTLFVPQILMIIIFWGDISILTDVGCTAIEVGQLVFKLLYLLLRYQKFG
NIYQTAKKLWPIIDNNEEKESFEKLAWLAQRFTVTFFVIGYSNIIALTTASIIIWIKNFS
HANDTLYTRALPFDVWYGIDTQSSPNFELLFCCQFVAMIFGAAGVIGFDTTMMTFILHTC
SQFRLICTRFCAFGNKIRNKSYRNFDYLHNWIGPKKDFDQCIKHHQYTISIAQEINVLLS
PVIFVQVMTGGLQICLSGYALMSGAEVNNRSKISKLIIYLATVFVQLVIWCWPGELLIQE
SIAVNDAIFYELPWYKFTTNQKRELIFLILRSQKECRMTALGYQVMSLRKLTEVFNTAVS
YFALLRSLSEEPMKQA
>AjapOR111
MDILPLQFRTLTYCGYWNKSDSNFNIFKGLWGFFISAVIFYFTLTQVIELYLLRNNVEDL
VDVMFLTVTYIVLCIKIINFNFRRKDLSKLLDDFRDEICQPESKEEKNILEKYTAKIKKI
FQVILSLSQATGVFFCILPFVTLTFRNYELPFKTFQFYDDTTFAGFSVTYFIQFVALIFG
IFINVSMDTMAYGFIMLASSQFEMISYRLIRTVRVNDKELLRKCVAQHMHVYGIVRKIQK
FFIGVIAPLFLLSLLTLCASIFQMSQNEIISLEFLGFAMYLSCMLCQIFLYCWYGNELKI
KSEDVANDVYKSDWTSLDLPEKKILRTLMLFAQKDSTISWQGQCTLSLNTFVWIMKTSYT
AFNLLQRVNVNGA
>AjapOR112
MKGSLKTQSALNFTKYSIILLRCWPPAKNSSKLRKFIFDLSWWFMFLSALCLLLPLIASI
KAYKSNSEIMTKSVCLSCAVSQCAIKMLFCRIRRDRLQKLLEEVEDFIKNANGLDRIVLE
KYVNQISTLHIIITIWVYVTSISFIIGPFLMNQPFPTDAVYPFRIDNIYMKIIIYAQQSL
VGLQTSAAVLLDCLVAVLLWFVCARFEILALSIGDYKNFEEFRDHIKRYQHLLSYTDDVR
RTINLFIFSTISTSVGGVLFSAIQLVADQHLAVKAQYAIVGVTASIGLFICSWAADSLVQ
MEYIVGMKAFESDWYKMEKKLKSCILHVIAQSQKPLSIKANRMLPTLSLVFFSQFLLTTF
KFFTTLRILVDSG
>AjapOR113
RPYEVVDSSRVRRILVYFVISTFMYPFINGCRIWCGINLEICCENIISIFFTGGILLKCI
VTTSSQNKIKVLLKIIAKNWLTFTDPGERKIILQYAKIGNFRAFGYFVYCVVTFTAFTMS
PLLPIFMDLVRPLNESRPKIMMLKAEFILDGNEHYYKIHTYVTLIGIIYIFVISGLDGCF
TVIAHQNLAIIATIKYRLQVAAKASDGLKNREHLLMVKSITLHKESIEFNDLVNGSYSIV
FLLINGITVIYLSLGTLIILKVIDDFKKEDLFELVRLSMLQFGMLIHLFYFSWPGQMLLD
HSEDLYRSVFMIQWYNISPQGKELLKILMLRLTKPNVYTAYGLFDMNFENYGTIIRTTLS
YITVALSFRGS
>AjapOR114
QKYKTKVFIRILFLVIIFSASIPVVSRGIKEIGKNLPIVLECIVICIIVITSFIRYTATF
AAEFKLKYLFEQITEHWKEFTDKEEQQILHYFAHTGFIWSRIFIVSIIFGLIIMVTGRTV
LPIVLDIIIPMNESRQKILSLYLDYFVDEQEYFNYIFLHIMIFGYVIASIYMVGDPTFIT
IVHHSVALFNIAGIQLRRAFEIVNDNKNNESKLIQNDVHNIVLKSIAIHLKSLQFVDDLQ
GAYSTCYFFMAIACVCSLSILCASAMMLIGDHFEFMRAVCFILITAYNLIYICYSTQKII
NASSDMFYSTYFCGWYEFPFKTKCLIHIMMMRSMRPCILKVGSMLDMSMVSAGMFIKMAL
SYFTAMTSMT
>AjapOR115
MLTGPGLWPNYHKYSYNFKISLNLFAIFTTCVTSYGLLMFCIKNAANIIVMTKGLSLFIS
LLTAFFKVFILMMHQKDLEYLSDNVSTDFERNLKISEDRQHLLAHSSIFSKFFYILNYFV
FASILLLVVMPLAALRHGKYLRMFPQVLPFSYEPGGTIHWIIYGFEIISGLHLWTVTSGS
DSLFGLFTLHIVGELRLLSYNFQKLKSSDNYKNDLKSCISKHTVLIKSKIALERTFGFLA
IWLAVSCAICMCALVFQLTATKSFTVFKICYSIFYVLLKLVQAYSYAWFGNIINVESRNC

LDSMYRAEWLGSGDIKFMSDVLIVLSQKPLIFKAKSLMILEMDMFTKIVNTTVSYFFLLQ
TLEESLEESS
>AjapOR116
MELFDGYYYKSCKKFMELTGLWPYQSSFRRHLSKVIYISVNMTLISPLAVLLYQEWCKNI
FITFENFCSLLFLCGFLGKYMMSAVCRAQFKVFLNIIAEDWQRIQDKSERDILSTYTEQG
RALNFMYIVLVFVSWIIFNLSPGLTLVLDVVFPLNESRPYFLPVYANYIFFKQLDYHYWV
CVHVAIVYFTCCCVLTGTDTTFVMCIKHNCGLFAIVCNRLENILKYDKESKTINIINDKS
IVQKLRQTIILHTTTLECFDALENTFTMVFLLQNTFCVTGLSIALVYVLFLFDDIMRLIR
IVTLICAVLIHLLYIHWIAQQIYNSSLDVFQAAYFSNWYVISIKARVLVQVIMIRSSFPC
RITAAI
>AjapOR117
GLWPNYEKHPRALRILLSFCSGFTNGTTCCGVLSFCINYVTNINLLTRGLGVVVSFSSAF
FKVCILSFHQKDLQELNEGLSSRFSRDLEIPEFRPYLLAHFRTCSRFFYAFDYSVAVNLL
IYALIPLSLIRHGKYPRMYPQVLPFSYEPGSIIHWSIYGFEVASAFFMWSVTCGVDSFFG
YYALQMVGELRLLSFHFEKLKSSDRYKEDLRICVDRHIELMRSKHLLEKIFGLLSIWLAI
TCAVCLCALIFQASETKHLSLLKVCYLICYSFLKLLQAYSYAWFGNIIAVESQLCLNSMY
NAYWPGSGDKQFMNDVLIVLSQKPLAFKAGGVMLLELDMFLKIINTSVSYFFLLRTLEET
FDESSL
>AjapOR118
MDILPFNFGSCKLSGMWNEDNNYILLKMIYKILVLLIIFQFTLSQIIEVAKMQGDVDELT
EVLFVTFTFVALCLKIMNFVIRRDEMFHLLNDFRLPICQPKSKEEKKIIQKCSYTSGKIF
LAIMSLSQSTGLVLLVLPFLNISEDGSFRLPFKTYQPYDISNPINFWITYILQVLATIYG
VVLNVSMDTMAYGFIIMVAGQFEINCHRLTNFTMDLKDCIEHHALIQDMVFKIQCFFIRV
IVPLFFFSLITLCTSIFQMSQKQVASFEFLSLTMYLTCMLFQVFLYCWFGNELFLQSKAV
TDAVYSSNWSNFTNKEKKNIWFIILSAQNGSNISFHGQCSLSLKTFLWIVKTSYAALNLL
QRASI
>AjapOR119
WPYMSYSNRISRRLLLYAILSVAMIPLGNGMRIWCGVNLDTCAENILGTLYTLAIFFKFW
FTTISEEKLKNIYKKVAENWLEITDPKEHAILINYAEQGRIKTVGYVVYMSLAAFCFCQL
PMIPKFLDFVMPLNESRPTILFVKGEFIIDPHKHFYKLYIYFAVASFVTMIAVTSIDTTY
TVIVHQILGIFSIIKYRLHLATISFDPLKDISYSIIVKAIHLHYRALQFIDLIEKSYSFL
FLMIMGFTISFISFGVVLLMMNTGEMLDAFRFFTLLIAVILHFLYITWPGQNIIDHSQRL
FQSTYANEWYNVSEKSKRLIRMIMLRCTKPCFFTAAGLYIMNLQNFGAIMKTTMSYIAVV
GSFR
>AjapOR120
RIFEMKGTNTNVEKNMDIERIESAFEDSHLKINKRLNTLVGTWPFRPTSDRILRYIIVIA
ALLCGILPHFEVLYNKIVENWMLITDPNEKSILDEYSKLGKLKTIFYVGYCLNAYICFTQ
IVMIPVILDIIIPLNQSREKVLITNAGYPFDPYEYYYELYLMYVVASLIATFIISSTDAT
HTVIIQQSLAVIALVKYRLEKISGERNDSYDLIISAIRMHQKGLEFLEFIESTYSIAFIS
FIGVTVTFLCCGSIIIVKSLDNPFELFRVSLFASGASIHIFFISWPGQLVIDGIENLHFA
TYTNEWYHQSEKSKTLLKIIMMRCSKINFLTAGGFYIINFENFGAIVKSTLSYVTVALST
I
>AjapOR121
IYCLNFFYSFTKICKMKHSINPKNFFDFLKFIGELIAIWPLKIGASKTEILIYDLKWCLA
FLNATGLLVPLIFGVYYFRYDSLTVTKTLSELTALSEVFINLIMCRHKRKHFQVVLKEIK
NFINNANEDEQIILQKYLNRYKDFQLFVGSSFISTALLFTCMPLVTSQILPADAWYPFPV
DTLLVRTLLYTTQVFAIFQTGFGIVVDLIVALMLWFSAVQVELLERRVSQAISIKELKFC
ARRHQDIILLVDNVKQGVQSIILKTNATMIVAVVCGAFQLIHHEPLEVLLRFILMVVAGC
LRLYVSAKPADDLKENNEHLAKSAIQTAFFTKISIFSKTCLNVSVSLSKTNGSFSSRGY
>AjapOR122
VNMTLITPLAVLLYQEWCKNILITFENFCSLLFLCGFVGKYMMSAACCTQFKVFLKIIAE
DWQRIQDKSERDILSTYTEQGRALNFMYIILVFASWIIFNLSPSLPLVLDVIFPLNESRP

YFLPVYANYIFFKQVDYHYWVCAHVGMVYFTCCCVLNGTDTTFVMCIKHNCGLFAIVCNR
LENILKYDKENKTINIINDESILQKLRHTIALHTTTLNCFDVLENAFTMVFLLQNIFCIT
GLSIALVYILYLFDDIMNLIRMSTLVCAVMIHLLYIHWIAQQIYNSSLDVFQAAYFSNWY
VISLKARVMIQVIMIRSSHPCRMTAGKIAEITMYGYGTLLKTVMSYFTVLLSVM
>AjapOR123
MGLSIFVVCSLFIPEILMITIFWGNISILTGVGCVAIPVGQLLFKLIYMGIYHNNFCHIY
ENLEKLWVVNDNKTEKRSLEKLAFLAKRFTIIFFLLGISNIFSFTIASIIVWINNYRRAN
GTFYVRDLPFDIWYGVNTQQSPNFEILFCSQTIAMVYGATGVVGFDTSMMTVILHVCGQF
RLTCIRLKNIGYKMYRDSHLNPEYESILIGELKQCIKYHQDIIRIAKEMNALLSPIIFVQ
LMTSGMDICLSGYATMMGISNNNQTEIIKGIFFVFTIFGQLLIWCWPGELLIQESTAVGN
AIYYEIPWYILKAKNQQNFSFIVLRAQKECTVTALGYQVMSLRKLTEVR
>AjapOR124
KMFEIRHYFHYFILSIPSQLYYTQFIGKNVVAYLARKKIKEILEKIKNDFQVYADKDLKI
LHDYSKKAHTFNNFYTVYMFIVVSAYSLLPLSLHLMDIFLPLNETRLAQTPRLIKYYIDT
FDNNILFIIIHGSIADITAIIFVIGFDTLYMSIAYHICGLFVILSNKVQDTVNKDINLQI
NSANQYSNQHQHTGYHNFVKTIIMHKYVLEFATTIESAFATLNLLSIGCAMIPLTITGFE
VLMNKGNFGEMMRYFMYAFAEIVHLFYYNWPGQKVREHSLLVYQACYNCEWYKEEFSIES
RKIMNLMMVRSQRPCYFTAGKIYVLGLENFAAVIKMSMSYFTVLSSVT
>AjapOR125
TVGIRLYMELGVNTDIVIENVIGEIYVFGISLKLFLIIISKKKIKTVYEKVARDWKIISD
AEELRVMHEYTEAGRKMTVMYTIFILIGVSIFLCLPIAFPLLDYIMSVENMTRLRKLPYY
AEYGIDQDKYYYPLMAQAVVGGTGTIIVLIAFDLGYMMIIQHIVALFAVVNCRLIKVSQI
SNGIEKRLIKFLDGDRKAYYYIVKAINLHQTGIQYVNLAENCYSVSLFLILFLNMVMFGG
GLTVVFYKNEPEELVRYGLVMISGLIHFYYMFLPGQKIIDSSLEVLQICYSCKWYNLSEK
TKNLLKILLIRSMRRCELTGGKMFVLCMETYCNMMKTGFSIFTFIKS
>AjapOR126
SAQCVRNWGNLDIMTDTLVLANLPIANATFMLGKLWFKKQVLRDLMEVVRDDWMKPKTKT
ELKIMNDIAKTSRTMSIICILSGQSICLYYFIFTVFQHLTIDMSDGKPLYFESYVPYESQ
STPRYEITWGIQFFCASFATCAFTGYENFIFVLIMHLCGQLLYLRHRIQNLTADGSLNKI
ENFRRFKKEIASIVEQHDYLNRIASIVEDTFNAVFLVQLINFSIIFCLLGFRAVTLMARS
KATLYEVVFTISFFAYLSFNLFVCCYAAEQLREESTSIEYASYDCLWYNMKPQMSKSLIF
ISMRSKKPLEITGGKFFVFSLSTFTSLMKTSASYMSVLIAMKNKQIQ
>AjapOR127
FIFSFMMSMGIKLYLELGVNLDIVVENIVGEMYQLMVFFKVTVGIVARKKLKRICILIAH
DWKTITDENELKVMHKYTEYGRKMITFYSGLMIISVMFFVSLPILLIPLLNYINPLENAT
RVKELPYKAEYGIDQHKYFYLLMGQAIFGGVGSISVFICIDLTFMMVVQHVVTLFAIVNS
RLSEVAKISKEIKNCMNDFLKGNTKSYNSIIAAITLHQRTIRYVDLVEDCFSVPLFVISF
VNMLMFSGGVAVVLIKLGGNPDEAIRFIGILIAGLIHFYYLMISGQKILNSSAEVFDACY
ACEWYNLSKNTKTLVNIMLIRSLRPCQLTGGKMFTICVNTYCNVSLN
>AjapOR128
SRGIKEIGKNFPIVLECLIALILIIATSIRYTATFLAELKLKHLFEQIMKDWKEFTDKEE
QKILHYFAHTGFIWARILIVLMIYGFILLTTAKTALPIVLDMIIPMNESRQKMLCCYVDY
FVDEQEYFNYIYIHILIFGYVIASIYMTCDTTFISVVHHSVALFNIADFQLRRALETIEN
KVNNKSISEENDVHKIILKSLAVHLKSIQYVNDLQDAYSMCYFIILVACIISLSLLMALA
MMLVGDHINFIRAVFGILILGFDMAYLFYSPQKVIDASSYMFYSTYFSGWYQFPFKTKRI
IHIMMMRSLKPCRLSAGPLMDLSLATCGNITKMAMSYVAAVLSMH
>AjapOR129
LSGLSSASVFCGIVAFCIYHGFTIQVVTKALGLLIGFFAVFVKISILANYQKDLLKLNEG
VSAVFENDLKIPESRKHLLANFSTFSKFFYVYAYAVGISDIFLISLPLTALKHGKYVRMF
PQILPFNYEPEGIIHWSIYGYEVYCGYCLWSVTCGTDSVFGLYALHIVGELRLLSKRFQD
LKTSSNYKNDLKKCVEKHLLLLESRNILERIFGFLAIWLAITCAIALCALVFQASQIKNK
TIFSTCYLMGHCFLKLVQAYSYAWYGNIINVESERCLMSIYNAHWPGSGDIRFMNDILIV

MYQKPLVFHAKSFMCLGLDVFTKIVHTTVSYFFLLKTLEEKS
>AjapOR130
VGSIFFFAVGLKFSYIKLLQNKLKILFESIMENWLILTDPEEKKIVSKYSELGRLKVFCY
TVYMLLSISAFIFAPLCPVFLDYIYPLNASREKLMVINAEFIVDKDKNYYKIVSYMAVLI
TIAMFIFGAFESCYTIIVHQNLALLSTIKYRLQVASKASIIEKNLDCAMMNKAIVLHKRC
IELNDLINDWYSLCFLLINVMTVLILSTGAVVTVSRLDDFDMEKDLFEIIRLSFIIVGVL
IHLFYFTWPGQLLLDISEDLFLSANDGSFHNNTKIYMSIIRFMIEWYNISPKGKQLLRIL
MLRLTKPNTSTAYGLFVMNYENYASVIKTTISYITVMVSFR
>AjapOR131
FRVLSGVHLVDGDNELSDTEGEREKRQIKRTMYIDCFTKLGIKSSSSTMTIKIEDSTEYE
KLMWPLITTFQPIACWPNRSEIEGVRNIKNVLRIFNRYLAIVIIFLITFGTISESVHYWG
EDMNDTIECSLIASACAMALFRVVLFSTKQKDMLFVVEMMKKDWTKSSIEDKLLLRHKCL
LAFKLAKFFIISVAVCSSSFVVLPLLEMMSGEVYKKELPFRGYYYFNHTGTPIFECVFAA
NAILGMIGCSTIAAATSFSLIATIHGAAKFYIVQQDFQSISSSEWVLSEKLTKCVQRHQE
CIRFAEAVEKVISYIALAQFMISTGLLCFAGFQLTTVLYI
>AjapOR132
IFQIWNAVDTIHDAENFLKNLDTTIAVIATVIKVLAFQIKNRSIRYVVQDVLNVWSSKGE
YNEEKFMMGNVKLANIIVNIVFTIYNGLTVTYMLIPILSYSGNRRFIFSATFPIKNPYRS
PIFEILCLSQGVSAIFCSNGHGIVEAVLVIMIMHAKNKVFNIRQEITKCIAANENSHSDQ
VLLNNIESLVRKHVNYLQFIKHLQNIFSIVALIMVGITTLIFVGLGYVLLYLLERNAQYV
KLFQSALYICCGLFALATYCIAGEYLTTQSQLILKDLNDCPWYEFSPANARLILFMIIKA
QKPVTITAGKFNDLSLILFTSIIKSSFSFLSLVRATNQI
>AjapOR133
MDEHEILNVKYYQDIKLLLKLTGLWPYQKYKTKKFIRIVLLIITYSATVPMISRGIKEIG
KNLPIVLECCVMLILIVTCSIRYTATLAAEIKLKYLFEQMTEDWKEFTDKEEQQILHYFA
YTGFIWSRIFIVSLIFGFILIATAKTVLPIVLDMIIPMNESRPKILSLYVDYFVDEQEYF
NYICLHIMTFGYVIACIFMVSDITFITIVHHSAALFNIAGYQLRRAFEIVNDNKNNKSKL
VQNDVHNIVLKSISVHLKSIQFVDDLQDAYSTCYFIMAIACVCSLSILCVLAMMLFGNHL
EFIRAVCGILITVYNLVYICYSTQKIINASSYMFYST
>AjapOR134
MDILPLHFRVCQFCGICQLAEVLTMQGTIDDVTEVLFMTFTYVTLCLKLLNFLMRNDELV
DLLDCLKRSIFNANSIEESTILHDYSDKANKIYLSFMTLTLPCGLIFMTVPFLNLKNEKL
RLPFKSYQPYSTANFINFSITYALQFVATFYGILLNVTMDAFVYAFILMTSGQLELNCYR
LKMSIMPLKDCIEHYVAIQDMVIKIQNIFIRVIVPFFCFSMITLCTTIFQMSEKRVLTFE
FLSLASYIICMFYQIFLYCWFGNELELKNNTVADAIYSSEWLEYTPKERKNMWFLMMFSQ
RAPNISYHGQCSLKIKTFIWIIKTSYTSFNLLQNASI
>AjapOR135
IPQTILLTQIWFEDLMITLENVVSILFYLCCYSMYFSLYMSADRIIFLFEQIYRDWKQIT
DQNERKLLKIYTNQGATLIRLYTVLLLSTWFIYTSVPLIGDFLFRKNESTPKFVPYFANY
IIFDQQDYHYWACAHLAIVYINHMHLCCGIDGVFLLCIKHICALFAITCHRLENVDPENK
DKLIVKLTVTSHIDAVRAVLLLEKSFNICFFVTNVASYAAFALGGLYIMNIRDTFRFVRS
IIFTISVFIHLIFLNWGGQLIIDRSVQVFQSAYSSKWYKSSQNIKVLVKIIMARCMIPTY
LTAGKLIILSFENFAFLIKTGATYLTVLRSIT
>AjapOR136
MDLELEKIYNNEFFKINKVLQETVGLWPYSSRNHKIIRRMIVLTVLGSVTLPHMNAVRIW
CGVHLGLCAENISGVVYAAGVFAKYIVTILYENKIMQVYEQVAETWMSIQDPQEKAIMIK
YAKIGKLKSVGYVVYLAMAGIGFSQLGIVPVIMDFIIPLNESRPKIQVVKAEFFFDPLDY
YYEIYFAYCIIASVSVTVLMAIDTMYTAIIHQNLGIFNVIKHRLQIATERRTDADKSYAY
NTMISVIQLHQYSLKFHDQLESTYDILFLILIIICILFLTFGAITIMENTGIILDVIRMG
LFEIGAAVHLFYLSWPGQLVISESSELFVFK
>AjapOR137
ICCENLLGFVYLFGIFLKILLTSLNEDKYKVILETIARNWLSLIDPEEKKIIQKYANLGK

IKSVGYFFYVIFTGIVFASAPMFPVFLDFVKPLNESREKIMVIKAEYIIDHSENYYKIYI
FMLTGVVHSVIVFSGIDGFYTIVIHQYLATLAVVKHRAQSAANELNVLKKRDYKILIEAI
IMHKESIEFNQLINVTYSTMFLLLNSMTVIFLSIASIVTLRILDDFVIEADLFELVRVSM
LEFGVLVHLFYFTWPGQMVFDLSEDLFISTYLSNWYNISKKGKELLKVLMLRLTKPSKLV
AGGLYDMNYENYGAIVKTTLSYITVAVSFRG
>AjapOR138
MKTKANELTTESLSFDQVTKIYLMFLRMIALIPFHGRDLNALPSRILLIYFTSSKITNFL
MFAGKLVDNLCKGILDAGCILETVIVVGTTIRYTFMLRWREEYSELVNECRTIWSNCSSS
EHLILCNYEKKLRTLFNIFFSSLVLVNVFYTGVSFVNLLLPSKENETKLRNLPYRWYINE
IHESPLFELVFLWQTIVVINESLLVTVLDTAAPLLIIMNCGYFKALQNRLFYLSQKKENN
GDDYEDVLACAKFHQQLLNFCVRIDKCTRTLFLIQLIATGYNLSLIGIKLVGTDPDRYKF
SSLLILCFVQLLICQWAPDSLLEEVCP
>AjapOR139
MDEEKLKVYDNRYFKISKDLLNFSGLWPYKAKIPIFIRRSMTLSVLFSTILLCVNATRVY
CGNQLRMCCENIAGIFYISSVSVKYVTAIICEAKRKRVYLLIAKNWLALTDPDEKSIITN
FAMQGRLRGVRYFAYMVSVGLIFSQSHLIIPIMDIILPLNESRPKVMIVKTKYPCDYEKY
YYVIYVGITIIGIASVGTFAAVDASYTIIVHQCLGIFSIVRHRIYKATENPTNDNFVCST
LISAIRLHQEALQFVDLIESSYNLSFLLIISCDVSCLSVGAILILESDDASDVMRMSMFG
VGIFMHLLYLSWPGQNLIDSSEN
>AjapOR140
MYLNAGVVKLTMTILNKRKHKQIFSLIARDWQMETEKDELEIMERHAAIGRTISVSYGIC
CCSTAGVFLSIPKFYPLLDFIVPLENETRPIVLPYHAEYFIDQHKYYYPLMTKALITGTL
SMTVFISYDMVFAMCVQHVCSLFEIISLRLEKASLIQKKTRSESQVRRQIIKAIQLHQIV
IENINSLEDTYNLNWFFILLLNTTAVGGGLLIVIIKLDRPIELFRYGMFVGTIFIHKYYV
FLPGQKILNYSFQVLDNCYSCKWYNLSPKCKVLLKIMMVRSIRPLSLSGGKMFILCMGTY
ATMLKASMSYFTVFANTM
>AjapOR141
MGTEVTTMNQNKYNERDFQWAFGLNRLILDLMGIWPNEVKSEHYIFKVLRVPILVLTILV
ALFLPQMYALTYVFSQLQLAVDNLTISCTTLTCTIKLFLLWSNRQVLKPLIKSAMNDWIS
MKSPWERKIMARQALFGRIFTISGYCIIVFCLIGILATPLFGISVRIINNLTDEIDGRFF
PLQTYYPYKATISPYFEITYVSQLIAASFVAISFSVPDNFFGVLVFHASAQCEILEAKMS
RLLPENDKRIMQQEGGEKRFIRMKLKKLVDTHVCLIRYVGGVEDCFNVLILVQIVGLSLT
ICCLGFSVIKVNNQKLF
>AjapOR142
GLAYLIGVFAKYTVPVISKEKFVIVYKKIAENWLLITDPKEKSLVEKYAKSGRFKTLGYV
GYCFFSGIAFSQMILVSVILDYIIPLNETRKKILTTKAEYPFDPYEYYYELYVVYTLATI
VSVSVLISTDTTYTAIIHQSLGIFAIVKHRLRIATEEKDPNASYDVIVSAIDMHKKALEF
VELIESTYQLAFLSFIAVTVVFLSFGSIILVEHSDDIIDLIRLSLFEVGAMIHIFFISWP
GQLVIDHTEDLFRATYTNEWYYLSRKAKVLLKILMLRCVKPCFLTAGGFYIMNFQNYGSI
VKTTLSYVTVALSFK
>AjapOR143
GVIYLSVVSLKYIITSLSEAKLKIAYEQIAIGWSTVSDPIEKEIIRQYAEQGRLKALFYF
LYSVCAGILFVNLPLIPVLLDYIIPLNESREKILFIKAEYIIDPYKYYYELYLFFVFDTS
IIVATFSSIDTTYTVIVHQNLGIIAVIMQKLKTATLNKKLDAGYIKIIETIKLHKDVLNF
IDLVESAYSFLFLLVMGATIIGISISALIFPHLKAFDSFRLFAILSGCILHLLYLSWPGQ
MILDHSQDLFVSIYTTEWYNVSQKAKVLLKIMMLRCMKLSKLTAGGLYVMNFENFGSIIK
TTLSYVTVAMSFQE
>AjapOR144
EKFISRITIYVTMFLILLPNANAVRIWCGKDFSLCGENIVGCIYSITIVLKYTTTILSES
RILRVHEQVAKNWTCITDKEEKSILINYAMQGKILTIIYIVYMSVAGFAFSQATILPVVL
DLIIPLNESRPKILIIRAEFGFDPFEYYYQIYAAYCVATVISVSVFMSIDTTYAAVVQQN
LGIMSIVQHRLHVATKYRNENKDKDVAYDTVIEAIVLHKEALKFVDLIESSYHLSFLFII

GFSILVISLGGVLITEYIGQTLEMTRMTIGLIGLFIHCFYISYPGQQLMDHSTDIFLHMS
VFRRIEISKFS
>AjapOR145
GLASAIEQIQMIFDDLTFFWNQYRPNKKCYVEILSKAEKSLFFCKCYVGVMISGIAAFSL
PPLHNFASQYFAREITNHTYDYSKRIFFLLYPFEVNSILVYFSILFEEQIVLIISAMLWI
NCDTLFAQLTTHTYLQFQILQYDIENLIDHENLEVNLTKNIIIFIKRHQGLLRICKLIEY
IFSPVILTTMLLSAMNICVNMFEFREMLSAGNNAETALYAIMVIILFFQIIFYCIFAESL
TEQTGLIAKSIYNCKWTEKNHRLRFYLQMIIMNSQTPFYCTAYGFFPIGHERFASIISTS
FSYYMMLQTMS
>AjapOR146
MVINDWMRSKSSWELNIMLKQAGRARMFTIAGICMMIASIIGFCITPFTGLKIRIVNNIT
DPDQYRYLPMQTYYPFDTVRSPQFELTYIFQLFAVALCGMMFSLADNLFALLVFHVCGQC
EILERKMSELVPDNFNEMVTLVKKDKQIFHQKVEELVKTHVYLLRFTASVEKSFNLLILV
QVIFMSSTICCMGFAAIISDGENERPPIIQIVTLMGTAMNLLIHTFICCVACEILATHSL
GFRSAIYNSNWYLLPAEYSKNLILIILRSKYPLKLTAGKFFYLSLNFFLRILKTSFAYFS
LLYSCQ
>AjapOR147
IIFNQMTDDWKQMKNTSERNILLSYDEEGRLINAIYVSFLFAAWMVYNGSPAIPLILDFI
IPLNESRPYFLTARCNYIFFEQLEHYFLECCHIALANFACVLILSGVDTTFIFCIKHNCG
LFAVLCYRLDNLFGKNENESIEIINKKSKISDLRDIIVLHSNTIKCFDIIEDTFNMFFLV
QNVSCVVSSAFALIYILHLLNDLVNFIRTTTIFITLCIHLLYFNLICQQISDSSQKVFEF
AYFSKWYILPVKARIIIQIIMIRSMYPCVITAGKMGEMSLSGYGIIVKTTMSYFTVFLSI
VDIQ
>AjapOR148
YKGIKIMRNRENLRYFISYYFNSQWTECFDSSENYIHEIIDLRIRNVTIAYTASMIGILF
LKAVTPLINAGSMFSLPVEAWYPYSIENLYWFWLTYLHQLILGCSAVSVHIGVDTLFVGL
LLKGYGQIDILKHRLRNLTKFYETNVQRKMIFRCIQHHERINRYGDDLNVQFQEILIILV
ITSLPNICINIHALSVYQDDVDIHYIATFFCTTSAFLQFFIACWFSNEVSLNSVQISSAV
YEMDWTPFDASTQKLLLFIMTRSLRPIQFYAGYFIPLNLNSFIMIIKASYTAFNMLQRTS
N
>AjapOR149
AVLLVITNFIRMDQYETHAFWSMIIKFEKLCGFYPYQTFKRNFSIFLLQAFAQITSIIVV
GARLIEEIGVNAPIVVENTVGMMVILCAIKKTTQCFIRQNKRKKIYDQIALEISIITDKE
EIETMEMYLTSGLKITKAYLSFSVFSVTPIIQMYLKHLFIENRTDSVKQIPIHVEYYVDQ
NEYYYCIMLHILVVFSLIFILITAHDFPFIMAVHHICSLFRIVANRLKKASNIMNKVENN
AKSFEIADDTSYNYILTAISIHKFAIRDVQNIDEVYNSTWFVVLMFNMIGFGGGLDFT
>AjapOR150
MFKKIKRQRSIDLNFFNGRSFEISKLFLKTIGLWPYQNYYERLFLHCLYVLLILSLAIPE
AILVTHIGLKELSITLENLISILLFFTTYVIYLSSYTSSERIKFIFEQIYRDWHQTTDEN
ESKLLEKYTNEGMYLIRLYTVYIAFTWTVYCIAPFLPVLVDFLFTKNGSTPKFMPHYADY
IFFNQDDYHYWACVHSAIVYIQPICLVCGIDGVLLLCMKHISALFAITCLRLENADPDNQ
NNSIVELTVTSHIDAMRAVLLLEESFTICLFVTNVSCYVAFAIGAIVHG
>AjapOR151
MKKEASRARIFTISGYIVLGGCYAGFAFAPLFGFNIRMISNITDYGGRYLLVQSYFPYDY
SKSPNFELTHISQLISGFFIGMCVSIPDNYFGALVFHATAQFEILGYHIENFIQNDESFK
GYKNRNFNRELGKYVDRHVHLLTMVSAIGQSFSFIILAQIFCMSVMVVCLGFQILGMFDD
SANKTSFFQMLVLMSTLFTLMMHTLVDCFACENLTMRSAGIFSNIYNCKWYLVPEQSTED
LIPMMVASKIPRQLTAGKMFYLSLATYCSILKSSLGYISMLIAVNRR
>AjapOR152
MLNMNIIVRHVKIGLIFMGAWPGLIPSKIIFVLLGMFVFSLIFQIWNAVTVINQLDLLMN
NLGTTMPVTSVVLKLAVLRMKFVSVKYVVNEMIEDSKTQDENIKHTVMKKNRKIAIYLSN
AIIACYNGLIISYLIIAVLSYNAESINDRQFIMQANFPINAKKSPIFEIFCILQFMVAFF

GTNGHALIEGLLMISVLHANTKAFGVCQEISKFSNICQAQMNRKNILKAKQELIKKHLNF
ITFAECIQDIYAYISFFHLFLMTLINCIVGYMLINVKYFLTIY
>AjapOR153
KSFSLRKIMDNVNLTEEVVDNIFDNQYLKLPKQLLRIIGFWPYESVTSSKMRQVIVYFII
ITFIFPFINGCRVWCGINLEICCENIVSIFFTGGILIKCIVYNASQNKLKVIFKIIAKNW
LSVKDPEEKKIILQYAKLGNFRAFGYFVYCTATCLAFSSIPLLPIIVDFVRPLNESRQKI
MIVRAEFILDEIDHYYKIHTYITSVGIVYIFVVCGLDTCFTIITHQNFAMIATTKYRLRI
AAEISDGSKNRDYWLIIKAIILHKESNQLSFIKNQL
>AjapOR154
KSFSLRKIMDNVNLTEEVVDNIFDNHYLKLPKQLLRTIGFWPYESVTSSKMRQVIVYFLI
ITFIFPFINGCRVWCGVNLEICCENIVSIFFTGGILIKCIVYTSSQNKLKVILKIIAKNW
LTVTDPEEKKIILQYAKLGNFRAFGYFVYCMATFVAFSSIPLLPILVDFVRPLNESRQKI
MIVRAEFILDENDHHYKIHAYITSVGIILVFAICGLDTCFTMITHQNFAMIATTKYRLRI
AAERSDGSKNQDYWLIIKAIILHKESNELSFLKIQL
>AjapOR155
FYNSRSKFFIKIVVVSLGLTAILYFTKPLIRQFNLSKSSDLTKTFTYDLPYRMHLLYKIT
NIKAYITTYISRFPFFYILVFTQTSMDCLTLTLVAHLCGQLGTLSIRISKITFEHGTEEL
NLAIKRHQKLVTIGLILRKIYHTCLLGHFLGASIGICILVYQVLSSISSGQKTNLVTFFI
FGFLNIFRLYTHCWAGEYLSHESFKISKAFYQCEWYKLPIKDQKSFIFCIKRSQKPLLLM
AGNLANFSLVMFTNVMKSAMAYLSFLRNFI
>AjapOR156
RIYIVIMIYGSTFMITSNTLYPMALDIVIPKNESRQKMLCCYVDYFVDEQEYFKYIFIHI
MIFGYTVACIFVISDTTGISIVHHSVALFNIASFQLRRAFEITENNNNKSQSVQNDVHNI
VLKSVAVHLKSIQFVDSLIDAYSICYSIIGIACACCLSCLIALVMMLLEDYLNLIRILCC
LLILLFDMVYLSYPGQKIIDASSYIFYSTYFCGWYEFPLRTKRLIHIMMMRSSKPCRLRL
SSLLDLSMYSTGVVIRMAMSYVTALLSMR
>AjapOR157
FVYMLCCAAVFCQAPLFPVFLDLINPLNESRDKILVVRAEYIIDPFENYYKIYAGFCFYS
VLSLFIISGIDGIYTVVTHQNLAILAIVKHRLRVATEVSNVALNRDYEIIIKAILLHKEC
IEFTRLVEISYSTFFLVINALTVIFSSIAAVVTLRVIDDFKAEDDLFEFLRISMLEIGIL
IHLFYLTWSGQLILSHSEDLLISAYTNEWYNISKRGKELLKILMLRFTRPCAITAGGLYI
MNFENFGAIIKTTVSYIAVATSFRG
>AjapOR158
IPLFLNIILPLENGSHPEIIPFVADYFLFYSYEYHYETCLHAIVIYFMVASLINAVVTLY
IAIVKHVCGLFAIVCYRLETIGKLDQCSKGDGSLLNFKRAEIVNRELGAAVLLHNEVMRC
CNLLEDAYSTSFLYSQAAIVAALVFGMFFILLVFEDLMPKIQMTGFFCGSVVHIFFLHWA
GQLIIDSSMVIFNSAYFSDWYLIPPNCRKLVNIIMSRSITHCQLISGKLIPLSMNSFGSI
MKTSLSYYTVLMSVGSY
>AjapOR159
FVFSQGPLVPALLDLINPLNETRYKPLFVRADYFMDPYENYYKLHTYFTMISSLSMTIFS
GIDGIYTAVVHQYLAILSIVKHRLQRATDASNVEKNRDYDMLIEAILLHRESIEFNDIIN
SSYSVFFLMTNGLTILFLSIAAVVSVRVLDGFNVEEDLFEFIRLTMLEIGALLHLFYLNW
PGQLLLDHSEDLFVSAYTNEWYNIPQRAKKVLRILMLRLTKPSILSAGGLYVMNFENYGA
IVKSAVSYTAMMISFR
>AjapOR160
LSIPTFYPLLDFVIPLENETRPVVLPYYAEYFIDQRKYYFPLMLKALIAGTLSMVVFITY
DMAFAMCVQHVCSLFDIIKLRLERASTLEKSGKASDRQIKELIIKAIRLHQIVIENVNNL
ENTYNVNWFFILLLNTTAVGGGLFIVILKLDHPEDLVRYGMFFTAIFIHKYFIFLPGQKI
INYSLQVFDNCYSCEWYNLSPKCKILIKIIMIRSIRPLRLTGGKMFLLCMETYASMLKAG
ISYFAVFASTQ
>AjapOR161
ALSYRIHPLIAGFGDPGPRDRVYPFFGRYYIDRHSDTAYLCCYLAQFWTGLVVEVTTNAS

DTIFFVCIYHACAQFNILQNNIAKLGRDEENGIERQKELIRKHQHEIRNAENLSKVFSIS
SFQQVMVSCVNICINGFKLIISLSEGDSDIGLYAVTFLLLLLQVFFYCFPGDQLISQSEA
VDEALYLSYWVNFDQPAKRNLEIMKIRAQRPLMILAGKMFVLSLENFMKIIKTSMSLLSV
LRAMYGE
>AjapOR162
SQLLLPSTHDQNSTRSLPFDFFVEVQTTPWFEIVLTLQGLTMFGLAITSNGIDTSGPFFI
MMACGHLRALSYRTQRCSTKILKKNYKTKDFAKTIISYIKYHQAILKYCYQIQTVMGGIF
VTQLISTTYNMALLGLKVVGEDPDKSKYATLIVVLLIQLLFLQWAPDMLINESKAVANDS
YLVPSFGYENRYVNQLLYIFIMRSQRPIELRAAGYVKLSMESFGKMITDILSFFAVLRNL
N
>AjapOR163
LDYIIPLNESREKILFIKGEFIIDPYKYYYQLYLFFTIDSAVIVCTFSSVDTTYTVIVHQ
ILGIISIVMHRLNIVTSSKQPIADYNKVVETIKLHVTVLHFLELIESIYSLLFLIIMGAT
IIGISISAIVFSSLNTFDLLRLVPVVIGFLLHLLYLSWPGQLILNHSEDLFVSVYTNEWY
NVSPRTKLLLEIMMLRCMKESQFTAGGLYVMNFENFGSIVRTILSYMTMVLSLQN
>AjapOR164
IQLETSTFRIYWLTYLLHTFGAISFTAISITNDAIITGFMLQTCGQIDLLQHRLKEFPRS
VLKIYETNESELSVKSLEKQCMTQIIQHHAHIYNIAKTICTTFSDIIVCQFCISAMEICV
SVYQLSVRTTNAVELFTYILYLVCMLGQFFVYCYFGNEVTIQSKKLSTTIFNIDWTPLSL
RLKKDLTFMLLRSSKPIAMSCGTLVNLTLESFVIILKTSYSAFTVLKRTA
>AjapOR165
KYRGFPFQGTSPNIFFVTPWYEIIYGIEAIFTLCRLIGCLSTDMICVTLLCQLISELKYV
IHIIERTKQNDKKSLFYIIKRHIIVMDYGVKVCDILSTTFLFQHIMILMFLGFAGIIIIS
LKNVFILMRLLPMAFIAVTTMFIICLVGETLTDSSLEIAKAIECNVYRLFLGDNISLKLL
NFILLRAQRPLCLKVYNQGSMNLNFFTQSLNKLVSFFIFLKTIME
>AjapOR166
VDQDEYFYYIMIHCLLCGISCGMTNITHDFSFILTTQHACALFEIVKIRLKKTSNEIYKA
EDGLITFDAANNLAYNYILDAIDLHTEALQFIVVIEKAYNITWLVVLIQNMVAVGGGLVI
ISLKKDQLSEFVRYFVTFCLFLIHFYLIFLPGQKVIDSSKSIFDTCYNCEWHKLSKKSKT
LLFIMMIRSLKRTNLSGGNLFVLSLETYAKMLKTGLSFFSVFAV
>AjapOR167
FHIIIILGCAFILITAHDLAYIMMVQQVCSLFKIVTVRLRNASKLTSKIDHNLVPYTFDN
NAAYDSIIKAINLHKSAIENIKNIDNVYNFTWFFVLMLNMFGFGNGLALLFIKKDDIVEL
IRYAITFSVVIVHFYMIFLPGQKVIDSSSEVFDACYDSEWYEFPKNIKLLILVMMIRSAK
HCELTGANLFVLCMETYSEMVKTGFSLFTVFAI
>AjapOR168
ELWMSLNDFEIMTTRNLVKKSVYLCYWYLSVCFLLILFYLVSSQLLNNHSQFNETQRRHL
MFPFYTDVQSNPWYEIILTMQLITHLGLDLGVAGVDTAATFFIMIVCGNLRSLSYRVDNL
SKINDLSEQRSMEKEIVNCIIFHKRIIEYTDQIREIMSNVFLIQLVTTTYNISLDVLTIA
KGGTSQLKYIPLFTIQVTQLFLCQWAPDYLVSE
>AjapOR169
RLKTLTYVALYSLVVFSFVLCPFFPIILDYVKPLNESREKIVMIKSEFFLDKKENFYTIH
AYLSMIGFTALVVYCSIDGSYTTMVHQNLAVLSIIKHRLQLINSASVIEKNEDCLILIKA
ISLHKESLEFNKSFNAAYQFAFFLLNLLSVLTVSAGAAPALRIIEEFKEKGESLELLQII
TMLCGVLLHLFIFTWPGQMLVDHSQDLFTSA
>AjapOR170
TIIGIIVFFAVDTFYVSSMQHACGMLAILGCRLKNIRRKRQSNYKISDDDFHDKIIGCAS
QHNNIIQYCEIINEFYSDSFFFIMGANMILMSFTGVLTILKWGYFYDMIRSGMFAGAQMF
HLFFYSFQGQKLIDQSLLISDCLYETEWYTASINSKKIMKMIIMRSLKPLKITAKVFILS
LPNFAVVMKTSMSYFTVLKSSR
>AjapOR171
IIFMTVDTMYVVCIEHCIGLFAIIKFRLRKPNKLIYNENSYDKKADASYEWVIETVQLHN

KVLKFTDAIEISYSASFLVIMSTNILCFSIASVLLVLSMDNTLNLIRFSMLLVGTIIHLF
YLNWPGQKLIDCTSGLYTDAYCNEWYLGSFKSKKLLKFFILRCAKPCSLTAGGLFAMNFE
NFLKLMKRSISYITVFSSFK
>AjapOR172
VDTAVVVSTFISIDTTYTVIVHQNLGIISVIMHRLKVATLCRQPNDYSKVIEAIRLHKTV
LHFIELIESTYSLLFLIVMGATIICISISAIVYHSLNTFDILRLVPVAMGFMLHLLYLSW
PGQMVLDHSHDLFVAVYVNEWYNVSQKTKLLLQIMMLRCMKPSEFTAGGLYIMNFENFAS
IVKTTISYMAVALSFQN
>AjapOR173
LKIVAKNYLILKAIPEENEILKKYGELGKSKAFAYFMYMVATAIVFINAPMIPMYMDWIM
PLNESRPIMLPCKTEYFVDTHKYYWEIYGFFLGLAFISVFILTGVDGAYTQMMHQNLAMI
SIVKYRLHKATQPLIIDLNQDYEILIDAINLHKDCIAFSDLLEKSYRTFFFIAIILTIIF
TSLGFGVVSNKFHFLII
>AjapOR174
LLFVFGAFESCYTIAVHQNLAILYTVKYRLQIATEASIIEKNLDCLMINKAIILHKKCVE
LNETINDWYSLSFLLINIMSVLVLSTGAVAILSRVDDFNLEREFVDLIRMLSLVIGVLFH
LFFFIWPGQLLLNASDDLFLSAFMIGWYNISPQGKKLLKMFMLRLRKPNTSTAYGLFIMN
FENYAAVRVLYP
>AjapOR175
CEILGIKMSRLLRKDRNHREKLFFRLTLKRLVDTHVHLIKFVNGVERSFNILILAEVLCV
SLVICCAGFGVITKIGKEGEHPPLVQILALTGTLMDLMIHMFAYCMASEILIKHSSSIFH
AIYCSNWLSLPGKFSRDITLIMVRSKYPLRITAGKFISLSLHSYLSILKSSAGYISMLLA
VNN
>AjapOR176
KDLQFNLKSINTTNDSERKKQNFVQSLKLVVERHLYLCRYAYCIEDNFNKVFLIQMICYS
VTICLQVYQLVTIFGSQTKNFGNLAFIFMYTCINLSSLFMYCFIAEQLRTESENIYFAAC
EVHWYELKPNESKMLTYIMQSSKSPLVITSGKFAELSLEYFAAILKTAGGYLSMLLAVQE
KYN
>AjapOR177
TCGMIGILKSRTQNALKGANFKNNTDNIKYRNLCNCINFHQSIYEFCDDINSTFTTSLFI
LVLVSMTLLSFTGVAAVIKLDDSSKLNDVLRFSIFASAQVFHLFFYSFLGQKIVDSMEKL
RDMIYDIKWYDAPIKTQGIVRLMISRNSKSISMQAVLFQLYLPTFTSVMKTSMSYFTVIK
STR
>AjapOR178
FRIMKIVTISESIENNSIDFFKGNNAAYHSTIAAINLHQRSIGYVNHIEDCYSIVFLFMF
THNVAMLGVAIVVILTKFDEPPEETVRYLLVFTSGLMHYYFLMLPGQKIINISEEVFNTC
YACKWYNLFGKSKILISIMMIRSLRHCQLTGGKMFPICMDTYCNMMKTGFSIFTVFK
>AjapOR179
HRLEKISGEQNDSYGLIISAIRMHQRGLEFLEFIESTYSFAFIAFIGVTVTFLSCGSIML
VKSLDNPFELFRMTAFASGAMMHIFFISCPGQLVIDSFKDLYLATYTNQWYHQSKKSKTL
IKIIMMRCYKTNFLTAGGFYIINFENFGAIVKTTLSYMTVALSTI
>AjapOR180
ANYTRALPLNLWYGLDIQQTPLFEMLFCVQPLAMLLGATGIIGFDSTMMTLILHICGQFK
LVHSRFRSIGDKLRVSDHSKLHYLKPILEIGNCIAHHRRAISIAKEMNNLLSPVIFVQLM
TSGLAICLGGYALLMGTESGNSTQTTQFVIYIITIFLQLVIW
>AjapOR181
LERDRIAVRNAVEAINLHQLVIRYVEQVESCYSICWLLILISNMILFGSGFAIVFMKDSI
EEQLRYTAGMISCSVHFYYVFLPGQKVIDISLEILDSCYACKWYNLSGKSKSLLKIMMIR
SLRPCQFTGGNMFLMCMNTYSKMVKTGFSIFTLFR
>AjapOR182
LHNTVLHFLELIESIYSVLFLLVMGATIIGISISAIIFSSLSTFDIMRLVPVAIGFMLHL
LYLSWPGQMVLNHSEDLFVSVYANEWYNVSQRTKLLLEIMMLRCMKRSEFTAGGLYVMNF

QNFGSIVRTILSYMTVALSFQN
>AjapOR183
IQMCTIGYVLMSGFENNSMSVQFVLNIDFLLDDIFGAGIYCAAGEYFLNQSEHIYQDLVD
CAWYEFERKNAKEMLILFIKTQKPVVITSGKFNNLCLVTFTSLMKMSFSYLSMIRATKAK
ELSNE
>AjapIR64a.1
MRKAKKIMAFLALTILSLKAASEKLDPSFTAEYFASKNVKQIVTFACWNEYDIFHYSRHI
MNANTKISYNTIQDDLNMKNILKVNYYRLGVVLDLDCPQSQTIFTQFSGFQLPFNDSYIW
LTVTRASEVPTTILNELPLTVESELTLALIHKSRYTMYDVYNHSYRHGGKLNVTLMGYWD
KSNGLNNQLTQYKYTRRQNFHGLTLNFSTVVINEPKPDLETYLTSTVDAHIDSMSRYHYA
LAVQLRDIYNFKMNLIRASTWGYKDANGTFKGILGDMINGIVDVSAVPFQYLEERMDVCE
FTVETWVVKLYFMFRHPSKNNVRNPFLKPFTQNVWWLVLNVSIICWILLLMIVKIENYFK
SKQGQTLYDNPSSETALITIAAVSQQGLSDGPHMYSGRIVFITLFVWALLMYQFYSASIV
GSLLAAPQKFINDVYDLANSDLRVVFEDTPYTRHYFGVTKDPASLYIYEKKMKSLQRKSL
WATPGDGFHEMSKGGYAYFTERATGYKIIEDTFTQAQICEFTELLLVPPQAVTIVTSKHS
PFKKMVIYGLQKIVEAGLMRRLLTIWQHRRPGCPESFRSQPVPVELHEFSPAMFMILGGL
ILGTIVMIGEYISWLTSHSRIDKVESEGTNSQGQSVWSVANDENSQILYPVMKQCFLHEL
PTINKILYDL
>AjapCG5621
DSRRLETPITKPYGMLKKTIFKMSGNDQYEGFAIDIIYEISKMLGFNYTFSVQTDNVYGS
FSQQTGQWNGMLKKIMDDEADLAITDLTITAERETAVDFTMPFMNLGISILYKKPKAAPP
SLMSFLLPFSANVWLYLIGVYIIVSVLFFIIGRICPAEWNNVYPCIEDPEELENQFTLKN
SLWFCLGAIMQQGSEIAPIGNSTRMLAGCWWLFCLIMVSSYTANLAAFLTVETVERPIKS
AEDLANQNVIKYGAKKGGSTLGFFKDSNSSTYAKMYHYMIANAKDVLTSDNDEGKRKVLN
DNYAFLMESSSIEYIQERECDLSQIGGLLDQKGYGIAMKKNASYRNELSGAVLKLQEMGI
LTALKNKWWKEKGGGGRCQEDSGGGQAEELGLANVGGVFLVLVVGITFSFLGTIMEFTYN
ILFCQKNNKFSWREKLTRELQFIIRFNDSTKPVKNQEL
>AjapNmdar2
MSSSVTWHFAASLIILYSILSLTLSEDDAGLSSRGSSVQRQASTIKIGKSDRSTSSNVAS
GGRGSSAIKIGEGFKFLNTTRVVRPVITTSMTSTSIPLAYASPQPESSSNGEFSNATKLL
KVGLAVPYKSFGYREYTRAVTKVLSALQKSTKRPNLGLFQRYDIYVKLAMQELTPSPMNI
LNSLCKEFLSLNVSAILYLMNYEQYGRSTASAQYFLQLAGYLGIPVIAWNADNSGLERSS
LHLQLAPSIEHQTAAMLSILERYKWHQFSVVTSQIAGHDDFVQAVRERISETQERFKFTL
LNAIIWTKPKDLMDLINSESRVMLLYSTREEAANILRDAHELKITGENYVWVVTQSVIEN
LQPSHHFPVGMIGVHFDTSSSSLVNEIATAIKVFAYGVEDFVNDPRNYGYSLNTQLSCED
LTADSRWNTGEYFFKYLKNVSVEADSGKPHVEFTQDGVLKSAELKIMNLRPGVNMQLAWE
VIGTWKSWEKDGLDIKDIVWPGNMHTPPQGVPEKFHVKITFLEEPPYINLAPPDPVTGKC
LMERGVHCRVAKESNVVVVQDGVDVQASTPRNGSTYQCCSGFCIDLLQRFSEDMGFTYEL
VRVEDSKWGTLENGKWNGLIAELVNRKTDMVMTSLKINSEREAVVDFTVPFMETGTAIVV
AKRTGIISPTAFLEPFDTASWMLVGFVAIHSATFMIFLFEWLSPSGFGINDYSSGSNRKP
VVPKRHRFSLCRVYWLVWAVLFQAAVHVDSPRGFTARFMTNVWAMFAVVFLAIYTANLAA
FMITREEFFDFSGVDDHRLARPTSHKPMIKFGTVPWTHTDSTLSKYFKEMYAYMKNYNKN
NVAEGIEAVINGDLDAFIYDGTVLDYLVSQDEDCRLLTVGSWYAMTGYGLAFPRNSRFLK
MFNQKLLEYRDNGDLERLRRFWMTGTCRPDKEVQKSSDPLALEQFLSAFLMLMVGILIAA
ILLLLEYIYSKYIRQHLAKDSRASKCCALLSVRDMLNPEMDVKT
>AjapGlu-R1
VGNHNQNTTNRKLELEVLVDIINTADAFKLSRLICKRFSTGVYSIIGAVNPDSFDTLHSY
SNTFQMPFVTPWFPEKVLMPSSGSVDFAISMRPDYHRAIIDTVRHYGWKKIIYLYDSHDG
LLRLQQIYQGLKPGNDTFQVETVKRIQNVTEALDFLRSLEELSRWSYKYVVLDCPTEMAK
DIIVSHVRDVTLGRRTYHYLLSGLIMDDRWESEVIEFGAINITGFRIVDGIRPHVKEFLQ
GWTRLDTALFPGAARNSISAQAALMYDAVFVLVEGLSKFMRKKTDKNNPRRTSNTSGVNQ
QPNATLNCYTNTSWVTHWEYGEKIARALRKVEIDGLTGQI

>AjapNmdar1
GNLQQCEQFEKTPNTLGLKNMAGVFILVGAGIIGGIMLIIIEMAYKKHQIRKQKKLELVR
HAADKWRGTVEMRKTKRSTDSRCINDNGVNDTATVSLAVDNVQNFMKHNRSPGKAWPGDN
ELRQIQDYPAQQLQSSIEYTYHSEATNLFA
>AjapIR64a.2
MLKKMLESLLPFLLYSLIVVGAYEINIKFIRNYFEERNIQQITVFGCWDEHENLDLFRKL
TELDKKLTLMPISDGLNMKEIMKVNYYKLGIFLDLECSGSTIILDQFVASRLPFNESYFW
LLTDSHLPLDLLNSLPLSINAELTLAVRDSQHGNGNYILYDVYNPSYRHGGRLNVTKMGY
WDVDNGITNELTQYKYKRRGDLRGIYLNFSIVVDFLPEDLDFSTYLTKPINRHLDTMHRY
NYALMLQLRDYYNFTMNLSRGETWGYLINGTFNGIVGDMIKGIVDVGATPFQYKSERMDV
MDYTVQAYMARAAIIFRHPKRQELNNSFLEPFTKQVWLLTVLIGLLNWILLYITIKIEIK
YFTSEDVSPLYTQTESETLLITSAAICQQGLSDTPRIYSGRIVYMSLFVWAMLLYQFYSA
SIVGSLLAEKARFIKSIKDLTDSNLEVGIENMQYNHDFFRTTIDKNVIELFDRKIALNKK
RKKFPYFKAEEGLEKVKKGDFAFQVDVATAYKIIADTFNENEICDLEEIELFRPQHTATC
TSKHSPFKKMITYGLRQIVEHGMTQRLGRVWKPTRPKCPESHNSQPTPVSLDEFSPVLVL
IAAALSLSIFIMLIENLVKKYVVHRRSSLASISSFDQ
>AjapIR8a
MSKGFRALLAIFVLRLTIAASQAPVTLLLVIEQPDAEILSSINNIVSDAENEFGANLIKM
DVQTVQVDREYVDENYEKVCAQLYKGITIILDMTWTGWDKLRDLANDFNIIYKRADTTIS
SFVQALDDIMMYKNTTDAALVFENEKELNQTLYYLIGNSIIRLVVIDYLSPQTVERIGSM
RPLPSYYAIYARTKQMEELFKTAVDGGLVRRDGVWYLIFTDYNYNEFSYFKGPSMVNVTI
NVLTMKDEVCCYLMYTSPPCTCPSDFKIFDHYFHRLINLIVEILSELQSANLLQEPQSGQ
CNMKNSSNPPMNATLSDFDKKLMAKIENNSTFEYVKKRTLIAYRAAVDLKVLSNGELTQN
ATWTRETEIVALPNRTIQAARRYFRIGTTEAVPWSIKKRHPITNEYMKEPDGSYIWEGYC
IDLIKKLATMMDFDYDLVIPDDGEFGQKVNGVWNGLVGDLAKGQTDIAVAALTMTSEREE
VIDFVAPYFEQSGILIVMRKPVRKASLFKFMTVLRLEVWLSIVGALTLTGIMIWVLDKYS
PYSARNNKHMYPYPCREFTLKESFWFALTSFTPQGGGEAPKALSSRTLVAAYWLFVVLML
ATFTANLAAFLTVERMQSAVQSLEQLARQSRINYTVLDNSTIHQYFKNMRSAEEKLYQVW
KEITLNSTSDQVEYRVWDYPIKEQYGHILQAITQVGPVKTVDEGFQKVIDSENAEFAFIH
DSSEIKYEVTRNCNLTEVGEVFAEQPYAIAVQQGSHLQEEISRRILDLQKDRYFESLTSK
YWNQSLKGSCPNADDNEGITLESLGGVFIATLFGLALAMITLAGEVIYYRRRNNQQDNAS
KSSIDKNNDMDNEQMMIKKLASKLQLKPAPVSFDVKPTLGVNKPQVSKISVYPRPFPFKE

>AjapIR25a.2
MSRAKETYRVVTVPKAPFVIYDAEKNFYSGMMIDLLNELARRLNFNYDIRIEKDLKYGYM
NDNGTWDGMIDELIEDKADIALGAVSLMSERLRVVDFTESIFKPTGISVLMRKPAVKTSL
FRFLTVLESDVWLCIIGAYLFTSLLLWIFDTWSPYSYRNRKEKYKDDPEKRIFSLKESLW
FCLTSLTPQGGGEAPKNLSGRLVAATWWLFGFIIIASYTANLAAFLTISKFEKNIESFDD
LISQYRYAYTVIEDSSTYRYFKRMNDIEYKCYEIWKDMTLNDSLTPYERAQLAVWEYPLS
DKYIKIYSAITHHSMLKNLSQGISKLEPEDSKFALITEAIDVQYQVLTDCKYKEIGPEFS
KKPLAIALQKNSPLTKEFNKIILKLIEEKWLTAIESKWWENNPDRQKCYDLDDTNNGIIL
ENIGGVFLLIAFGVQLAILTLVYEFFYYKFLRHRFEKLYHQNLMKFKFIASRKLKVKRIS
IKP
>AjapIR93a
SDNVVRAKFATRCQRNMMSLIVAVLLVVRFKFIIGYNDFPSLMTANATMAVIVEKSFFKD
VEDYQRTLMHISEIITSVIKKHMQISGIDVFVFEDVNTNLGRDYTVLLSVASCPSTWHLF
KRAQKEKLVHLAITDPDCPRLTEDNGISVPLIDPGEELPQIFLDLRMSSTISWPKINFIY
DDTFARDTISRVVKALSMELPNKRLTLSTSALFSTKFEKEESVMRQRVHKILGDFHVDHL
GTCFMVIVTIDMVPVIMDVAKSLKMVHPSSQWLYIISNGTGREMKVASFAQLLGEGENVA
FIHNSTNLDSGCNMGLMCHAKELVRALAISLENSLLNELELYDRVTEEEFEVVRLSKAER
KNEIIKNMNRELKNARSTNAASRCGECVNWKLTASLTWGLSFTNNSNDTNQQSYNKNEAD
SNIKGVLIDAGHWSPGFGVNMSEPLFPHISHGFRGRNLPVSTYHNPPWQIIKYSNTGTIE

YSGLTFDILNYLSLKLNFTYTIRLPSSFRLAIESDRNSYTKSSKSKHLDIATMSVAKKMP
SEILDLVRTKKVFMIAGAATVIENKRGVNFTASIATQTYTLLSARPKTLSRALLFTAPFT
SETWACLSSMLILVGPILYLMIKLSPRPIDAHDPIGLDSTWQCTWYIYGALLQQGGMSLP
KADSARLVVGTWWLVVMVLVATYSGNLIAFLTFPRTDATIDSVDDLLGRSQEFTWGFPNG
TALEKYLAVTNEDSYKELLSGATRVDPMKPYQALERIKNEKYVLIDWRISLSFLMRTDLN
KTGKCSFHIASDDFMHENMAMMMNGDSPYLPLINKAIKRMHESGLIAKWTADLTPMKDMC
SEGMSQEATNHKVDMGDMQGIFFVLAIGFTIAVIFIGSEFFWHKRKLAAEKKFIRPFVS
>AjapIR75d.1
MANKQVTLLVLLGILTHLPYQLNASSLTSEDDELLANVLNNIRGESSAFFSGAFAMVTAF
TCNHDATSKVQLLRRLSGNFVSSNVFDITEIDVILGELAWRTGYKGQELADDAHQQLFTL
DLDCLGVEEFLLKANEEMMFAAPFKWLMYKRTDDTADSLHSTFAQLSIYPDSAVNFWQLN
TNLIYSIYKINSKEIYTVEDRGYWIPENNEIQIKDRYTASKRRWNLRGTLLKSCLVITDP
DTINHLTDYQDRHIDSITKCNYPWALHLVNMLNATITFEYANTWGYQSPNGSWGGMIGML
QRGEIDFGGTGTFLLKERIGVVQYISLSTPTGSRFIFRRPPLSSVSNLFKLPFRNSVWFA
ICVLVVILTIVLYPAMRYEWSQSSPWEIFNEPEPPNITDDLLVVVGAVSQQGSWYEARSL
PTRIIVLMGLLAALNLYAAYTANIVALLQSTTTSINSLKDLLDSPLTLAAHDNVFNRYYF
KSYKDPIRRTIYEERIEPRGVKKNNWLTIEDGVDRIRRGYFAFHVEPGCAYKIIQETYEE
DEKCGFHEIDYLNVFDPHFIVKERSPYLELLRVGSLRLHESGVRSRDIDRLYTKKPVCGS
ARRFLSVGLSECYGAFMTLGFGIALAFGFFIAEIFNVKVKTLRNKSA
>AjapIR25a.1
MAEKTYRVVTIIKPPFVYYDSITNTWSGYCFDMLNEIANIVKFNYTVREVDHYGNMDDYG
NFNGMVKELQDNQADIALGALNIEAELQKVIDFSSPFYESIGMTVIRKRAVQPLTLFKFI
TIFDIEIWLSIIGSVLFTATLMWIFDAFSPYSYRNNKDKYNNDDDKRIFTLKESIWFCLT
SLTPQGGGAAPKNLSGKLICATWWLFGFIILASYTANLAASLTIARFQKTIESLEDLIHQ
YKVQYSPIMNSTEHIYFQRRSHIEKVYYEVWKDMVLNDSLSDYERAQFAVWEYPVGDKYT
KLITLMEDVGMPHNLSAAIERVRTQPPPYEYFLIANAADVRYLLMTECDLVQVGGEFSPR
PLGIGLRKNTGLKEKFDEAMSLMQDRRSFYNLKSKWWDHNPYKKWCGKPEEVSDGLNLRN
IGGIFIMIIIGILIASLTIIFEYECYVHQEKIDEVFLKITNWLKGLFQFRKKAVESFRPM
KEKKIKK
>AjapCG3822
QSICDTMEIPHLETRWDFRLKREGCLVNLYPHPTTLSKAYVDLVKALGWKSFTIIYENNE
GLVRLQELLKAHGPADFPITIRQLGEDSKVGHGYRPLLKQIKNSAESHIVLDCSTEKIYT
ILKQAQEIGMMTDYHSYFITSLDLHAVDLSEFKHGGTNITAFRIVNLEKTQLVIQDWIFG
EQRYSRKLRVEHNENNYTFIKTETALMYDAVHLFARALHVLDTSQQIDIKALSCDLTDTW
DHGYSLINYMKNVEMTGLTGIIKFDNQGFRSDFMLDVIELNTREGLKKIGKWNSTNGVNF
TRSYGEVYTQIVDNLHNKTFIVTTILSAPYCMWKESSKKLSGNAQFEGYSVDLIHEISRI
LKFNYTIQLVPDGRYGSYNRETKEWDGMIKELLDQKVDLAIADLTITYDREQAVDFTMPF
MNLGISILYRKPVKQLPNLFSFLSPLSLDVWIYMATAYLGVSVLLFILARFSPYEWESSH
TIGNPTTALENEYTLLNSLWFTIGSLMQQGSDIAPRAISTRMVAGMWWFFTLIMISSYTA
NLAAFLTVERMDSPIESAEDLAKQTKIKYGALKGGSTAAFFRDSNFSTYQRMWHFMETSK
PPNEVFTKSNVEGVERVVKEKGSYAFLMESTSIEYVIERNCELTQIGGLLDSKGYGIAMP
PNSPYRTAISGAILKLQEEGKLHILKTKWWKEKHGGGSCRDDTSKSSSAASELGLANVGG
VFVVLMGGMGVACVIAVCEFLWKSRKIAIEERRQRSSEKPICIGFTDFQ
>AjapIR75f.1
IIDDTAFGVSTDFVVAESSSTDSFNLYDIYNVWKGGGGSLNITLMGYWSKLEKVNIILNK
AKCLRRSNLNGISLRTSFYASKYKPPDMNITDYFQDYSTTSRDGLSKFGYIIVNHLSELY
NFSVQYGIVAVWGKGDTIGPVIKALQNNNIDLTGSLVTMSTQRTYMVKYVHQSWPFRTCF
IFRSPPTKDIKIGEVLKSFDVEVWYLIIIFSLITMWVTAVVLGFENSDNAIIRFSNSFLI
FLAAICQQGTDIKMNRISMRIVFLSILIFSLLIYTYYSSGIVYIRLNDPIFKINDSLNEF
AKIDIKMSSEYMVYFDLFMRKKDWETQIFYKKRWSRIPDSEQFMDPEIAIRLVQKGGFAY
HMHPDVGYPIINRLYENREICELNEVHVAQPTLTAFAVSLNNSFAELMRIGLIKVSEVGL
RHRQVLRWTSEKPKCLKNVLSASSVNIYEFSPHLLVLMIGMIFASLVYMIEIIIAKHTKV

ESLLPW
>AjapIR75f.2
MDLTDYLQDYSSTPRDGLSKFGYMMVTHLSELYNFSLQYGIVAVWGKGDTIGPVIKALQN
NKMDLTGSPVTMNTQRTYMVKYIYQSWPFRTCFIYRTPPTKDIKIAEILKSFDIVVWYLI
IIFSLITMWVTAVVQGFDNSDNAIIRFSNSFLIFLAAVCQQGTDIKMNRISMRIVFLSVL
IFSLLMYNYYSSSIVSVRLNDPILKINDSLSELSKIDLKMSSEYMVYFELFIKKKDWETQ
TFYKKRWSQIPDSEQFMDPEIAMRLVQKGGFAYHMHPDIGYPIVNRLYENREICELNEVH
LAQPTYAAFAVAINNSFIELMRTGLTKLSEVGLRHRQLLRWTSIKPKCLKNVLSASSVNI
YEFAPHLLLLMIGMIFASLVYMIETIIAKHTKVESLLPW
>AjapIR75d.2
KKKKLKIMWKFFVISLISQAFLYFIPVDSHEIDELIFNLIADASVALFLPAASISALLCA
KTDNVIELSRILSENQILFNQLNFENFEKDMKIPFHQTIILLDFDCPQAIKVLEKANNLE
LFSAPLKWLILQDLRKVSNNCTDDCLLTDFKSLAMYPDSEVIILRRISEDYVKILSLYHP
SPVRDMMIEDRGFWKSNEGIAMRDDYVASRRRRDLQETPLKSSIVVTNPDTLNHLTDYQD
KHVDTITKCNYIWLHHLVYAMNATVTYNIVNSWGYRDKNGSWNGMMGYLSRKEIDIGGTS
MFLVGERWDDAHYIPLSTPTRAAFIFRQPPLSFVSNLFTLPFRPSVWTALGILLSLIFFM
LVISTKWEWTVTDNDPLQSPTLSDNFLVIIGTVAQQGFGLKPRTVPSRIVVLMLLLAALN
LYASYSANIVALLQSTTTSIQTLRDLLNSPIKCGAQDIVYNRYYFKLEKDPVRRGIIDQK
IEPKNSKSNWMTADEGIRHVREGFFAFFMETGPGYKIIQETFEEDEKCGFREMYFIEHFD
PTFTIVKQSPYVEIIRVK
>Ajap84a
MGINNDDVGHLLAITIVSLMLFTSLTKTQLVTNAITYKHMVEAVHTIFNNTCVIFMYTVE
DPMQVLDPYDAEQLMSLPLAIARLHVRTTAYAIKTFKKQIGEEYFNLKRPLFILINDSEE
LRQQFSKEIAPWISMAYINWLIFFKDNTSIEYFFSEIYVPLDCIFLIAHKAKDSDTYELT
EVYHIAKGRELMIDHFGTWNEEDGLSITRLGLYQRRSDLRGQLIRVTSIQDPPVSLIEHD
EFGKMTGIKGFFGTIIQILQENINCSVIYMECDSWGSRSQNGSWSGTIGMLTRNESDLAA
AELLMTGDRLEVIEYTTPVFSTKCRTFIKRPYFTAIKWTAYSDPFYLGIWVSILAVMILS
TIFILIGFQLSPSRKFCRKEDLEQAFAYTFFDIFGALCAQGLDTVPIDPIRIIHLVVYMT
GVIILAAYSAALISFLAVKVFVMPFTSMEGLLEDGSYKFGVVQDSADYTFFERTTDKILE
RMYLEIMEDKSELPKNYYEGLNKICMEEKYAFMTTDNMFSILAHQVPCILEPLQVIMQTT
MAMAVPKRSPFRGIINSNILLMRDSGVLQRVIATELLLQPSTNKDGWSSVEIVDILPLLL
LILGGSFFGLIFLLFEKLKYVKFHKKLKIMLSSKLNEQKSFTQ
>AjapGlu-R1B
MTVNSAPVKVAEWKDLSGYHVVPTKRDRHPKSEILRNRTYIVTTIVEEPYIMEKKFENGK
PPEKMKDRYEGYCRDLADLITKKLNISWDLQIVKDGKYGSENSAVPGGWDGMVGELIRKE
ADIAIAPMTITSERERVIDFSKPFMSLGISIMIKKPIKQNPGVFSFLKPLSTEIWVCVIF
SYIGVSIVLFIVSRFSPYEWRVLTFGTSRDPSLVSRDGTMQHSHGPQGSPHGQHTSMAND
FSILNSLWFALGAIMQQGCDISPRSISGRIVGSVWWFFTLILISSYTANLAAFLTVERMV
TPINSPEDLAAQTEVQYGTLAHGSTWDFFRRSQIELYKQMWRFMNENKEDVFVNSYDEGI
ARVRNKKGKYALLIESPKNDYINEREPCDTMKVGRNLDAKGFGIATPLGSPLRDSINLAV
LSLKESGELAKLMNKWWYERTECRHSDKQDASGNELSLSNVAGIFYVLIGGLLLALAVAL
VEFCYKSHTEATRAKIPLSDAMKAKARLTIGGGRDFDNGRWYGLQS
