# Supplementary material for: Identification and Comparative Expression Profiles of Candidate Olfactory Receptors in the Transcriptomes of the Important Egg Parasitoid Wasp Anastatus japonicus Ashmead (Hymenoptera: Eupelmidae)
Source: Plants (Basel). 2023 Feb 17;12(4):915. doi: 10.3390/plants12040915 (PMC9962093; doi:10.3390/plants12040915)
Supplement: Supplementary file 1 [file plants-12-00915-s001.zip › plants-2102705-Supplementary Tables.pdf]

Table S1 Summary of functional annotations of *Anastatus japonicus* unigenes.

**Databases Annotation**	**Number of Coding Genes**	**Percentage (%)**
Annotated in NR	15495	93.00
Annotated in Swiss-Prot	11141	66.87
Annotated in KEGG	7892	47.37
Annotated in GO	11023	66.16
Annotated in eggNOG	11016	66.12
Annotated in Pfam	14901	89.44
Annotated in CAZy	940	5.64
Annotated in Signal	2184	13.11
Annotated in Tmhmm	3265	19.60
Annotated in all Databases	56	0.34
Annotated in at least one Databases	16661	95.35
Total **Coding Genes**	17474	100.00

Table S2 Sequence information of putative odorant receptors in *Anastatus japonicus*.

Name	Unigene reference	ORF (aa)	Status	TMD (No.)	BLASTx annotation	E_value	Identity (%)
AjapORco	g660	473	Full	7	NP_001164465.1 odorant receptor 1 [Nasonia vitripennis]	0	89.26
AjapOR1	g10601	600	Full	7	NP_001177477.1 odorant receptor 22 [Nasonia vitripennis]	2.00E-139	55.39
AjapOR2	g8192	450	Full	8	NP_001177501.1 odorant receptor 60 [Nasonia vitripennis]	8.00E-162	61.15
AjapOR3	g8571	439	3' lost	6	NP_001177548.1 odorant receptor 147 [Nasonia vitripennis]	4.00E-91	40.71
AjapOR4	g4600	429	Full	7	XP_014238328.2 odorant receptor 22c-like [Trichogramma pretiosum]	1.00E-142	53.49
AjapOR5	g10548	423	3' lost	6	XP_016836814.1 odorant receptor 49b isoform X3 [Nasonia vitripennis]	2.00E-126	50.13
AjapOR6	g9520	423	3' lost	6	NP_001177552.1 odorant receptor 161 [Nasonia vitripennis]	4.00E-157	52.26
AjapOR7	g10075	422	Full	6	XP_014238328.2 odorant receptor 22c-like [Trichogramma pretiosum]	2.00E-132	52.63
AjapOR8	g10985	422	3' lost	5	XP_031783507.1 odorant receptor 43a isoform X8 [Nasonia vitripennis]	5.00E-111	52.91
AjapOR9	g6493	421	Full	6	XP_014238328.2 odorant receptor 22c-like [Trichogramma pretiosum]	2.00E-122	50.48
AjapOR10	g11019	421	3' lost	6	NP_001177480.1 odorant receptor 25 [Nasonia vitripennis]	0	69.25
AjapOR11	g10738	420	Full	6	NP_001177429.1 odorant receptor 2 [Nasonia vitripennis]	0	76.72
AjapOR12	g12166	420	3' lost	7	NP_001177548.1 odorant receptor 147 [Nasonia vitripennis]	9.00E-121	46.74
AjapOR13	g3500	419	Full	7	NP_001177431.1 odorant receptor 5 [Nasonia vitripennis]	0	67.59
AjapOR14	g6167	419	3' lost	8	NP_001177543.1 odorant receptor 140 [Nasonia vitripennis]	1.00E-136	51.27
AjapOR15	g12002	418	3' lost	6	NP_001177547.1 odorant receptor 146 [Nasonia vitripennis]	1.00E-92	40.15
AjapOR16	g10901	416	3' lost	6	NP_001177548.1 odorant receptor 147 [Nasonia vitripennis]	3.00E-96	37.62
AjapOR17	g11127	416	3' lost	6	QGW50380.1 odorant receptor 53 [Chouioia cunea]	2.00E-165	58.63
AjapOR18	g10039	414	Full	6	XP_014238328.2 odorant receptor 22c-like [Trichogramma pretiosum]	1.00E-101	44.02
AjapOR19	g9254	413	Full	7	XP_014238328.2 odorant receptor 22c-like [Trichogramma pretiosum]	1.00E-131	54.33
AjapOR20	g6147	413	Full	6	XP_014238328.2 odorant receptor 22c-like [Trichogramma pretiosum]	2.00E-113	46.88
AjapOR21	g5495	411	Full	7	NP_001177489.1 odorant receptor 38 [Nasonia vitripennis]	1.00E-165	57.25
AjapOR22	g1743	410	Full	5	NP_001177572.1 odorant receptor 196 [Nasonia vitripennis]	9.00E-129	51.01
AjapOR23	g11721	409	3' lost	6	NP_001177548.1 odorant receptor 147 [Nasonia vitripennis]	6.00E-116	43.5

AjapOR24	g10917	408	3' lost	6	XP_016836814.1 odorant receptor 49b isoform X3 [Nasonia vitripennis]	5.00E-120	53.66
AjapOR25	g12437	408	3' lost	6	NP_001177548.1 odorant receptor 147 [Nasonia vitripennis]	1.00E-104	44.13
AjapOR26	g7009	408	Full	5	NP_001177511.1 odorant receptor 79 [Nasonia vitripennis]	2.00E-111	43.88
AjapOR27	g7240	407	Full	6	NP_001177435.1 odorant receptor 9 [Nasonia vitripennis]	2.00E-179	62.97
AjapOR28	g6548	407	3' lost	6	NP_001177548.1 odorant receptor 147 [Nasonia vitripennis]	1.00E-104	44.7
AjapOR29	g10295	406	Full	6	XP_031784325.1 odorant receptor 166 isoform X1 [Nasonia vitripennis]	3.00E-106	41.94
AjapOR30	g11575	406	3' lost	6	NP_001177548.1 odorant receptor 147 [Nasonia vitripennis]	7.00E-133	47.09
AjapOR31	g8361	405	Full	6	NP_001177548.1 odorant receptor 147 [Nasonia vitripennis]	6.00E-72	36.34
AjapOR32	g8618	405	Full	6	NP_001177467.1 odorant receptor 10 [Nasonia vitripennis]	0	75.37
AjapOR33	g9567	405	Full	5	NP_001177467.1 odorant receptor 10 [Nasonia vitripennis]	0	67
AjapOR34	g9436	404	Full	7	NP_001177510.1 odorant receptor 78 [Nasonia vitripennis]	0	73.9
AjapOR35	g11112	404	Full	6	NP_001164670.2 odorant receptor 76 [Nasonia vitripennis]	8.00E-129	47.42
AjapOR36	g11285	404	3' lost	6	NP_001177701.1 odorant receptor 153 [Nasonia vitripennis]	3.00E-130	50.39
AjapOR37	g12644	404	3' lost	6	NP_001177546.1 odorant receptor 145 [Nasonia vitripennis]	1.00E-116	45.54
AjapOR38	g10377	403	Full	6	NP_001177477.1 odorant receptor 22 [Nasonia vitripennis]	4.00E-99	41.94
AjapOR39	g11011	403	Full	6	NP_001177522.1 odorant receptor 103 [Nasonia vitripennis]	0	68.11
AjapOR40	g11246	403	Full	6	NP_001177548.1 odorant receptor 147 [Nasonia vitripennis]	7.00E-103	44.11
AjapOR41	g9730	403	3' lost	7	NP_001177548.1 odorant receptor 147 [Nasonia vitripennis]	2.00E-107	41.81
AjapOR42	g10420	402	Full	6	NP_001177473.1 odorant receptor 17 [Nasonia vitripennis]	7.00E-165	63.57
AjapOR43	g10903	402	Full	6	NP_001177547.1 odorant receptor 146 [Nasonia vitripennis]	5.00E-88	39.8
AjapOR44	g11057	402	Full	6	NP_001177546.1 odorant receptor 145 [Nasonia vitripennis]	3.00E-88	37.47
AjapOR45	g11625	402	Full	6	NP_001177548.1 odorant receptor 147 [Nasonia vitripennis]	6.00E-87	38.04
AjapOR46	g10014	401	3' lost	6	NP_001164404.1 odorant receptor 141 [Nasonia vitripennis]	1.00E-140	50
AjapOR47	g11338	401	Full	5	NP_001177547.1 odorant receptor 146 [Nasonia vitripennis]	8.00E-176	60.61
AjapOR48	g6919	400	Full	6	NP_001177549.1 odorant receptor 151 [Nasonia vitripennis]	3.00E-107	41.48
AjapOR49	g9561	400	Full	6	NP_001177477.1 odorant receptor 22 [Nasonia vitripennis]	5.00E-128	56.12

AjapOR50	g9377	400	3' lost	6	NP_001177548.1 odorant receptor 147 [Nasonia vitripennis]	5.00E-118	48.06
AjapOR51	g12018	400	3' lost	4	NP_001177548.1 odorant receptor 147 [Nasonia vitripennis]	5.00E-148	51.15
AjapOR52	g3648	400	3' lost	4	NP_001177621.1 odorant receptor 292 [Nasonia vitripennis]	3.00E-103	48.56
AjapOR53	g11038	400	Full	5	QHN69155.1 odorant receptor 24 [Sirex nitobei]	8.00E-69	36.44
AjapOR54	g10685	399	Full	6	NP_001177549.1 odorant receptor 151 [Nasonia vitripennis]	6.00E-99	40.97
AjapOR55	g11934	399	Full	6	NP_001177605.1 odorant receptor 264 [Nasonia vitripennis]	2.00E-114	41.16
AjapOR56	g7118	399	Full	6	NP_001164405.1 odorant receptor 154 [Nasonia vitripennis]	0	72.66
AjapOR57	g10927	398	Full	7	NP_001177567.1 odorant receptor 191 [Nasonia vitripennis]	1.00E-174	61.81
AjapOR58	g10754	398	Full	6	NP_001177548.1 odorant receptor 147 [Nasonia vitripennis]	5.00E-90	38.6
AjapOR59	g11139	397	Full	6	QGW50380.1 odorant receptor 53 [Chouioia cunea]	2.00E-165	58.63
AjapOR60	g10662	397	3' lost	6	XP_011501530.1 PREDICTED: odorant receptor 13a-like [Ceratosolen solmsi marchali]	5.00E-144	53.99
AjapOR61	g12212	396	Full	8	NP_001177556.1 odorant receptor 170 [Nasonia vitripennis]	8.00E-79	35.82
AjapOR62	g10791	396	Full	6	NP_001177548.1 odorant receptor 147 [Nasonia vitripennis]	4.00E-104	48.04
AjapOR63	g12087	396	Full	6	NP_001177548.1 odorant receptor 147 [Nasonia vitripennis]	2.00E-145	49.87
AjapOR64	g4795	396	Full	6	NP_001177549.1 odorant receptor 151 [Nasonia vitripennis]	4.00E-145	56.63
AjapOR65	g9668	396	Full	6	XP_031784326.1 odorant receptor 175 isoform X1 [Nasonia vitripennis]	1.00E-115	48.1
AjapOR66	g10607	396	Full	5	ANG59311.1 odorant receptor Or1 [Chouioia cunea]	3.00E-55	30.84
AjapOR67	g10138	395	Full	6	XP_031784326.1 odorant receptor 175 isoform X1 [Nasonia vitripennis]	1.00E-108	46.97
AjapOR68	g10918	395	Full	6	NP_001177567.1 odorant receptor 191 [Nasonia vitripennis]	1.00E-174	61.81
AjapOR69	g9132	395	Full	6	NP_001177552.1 odorant receptor 161 [Nasonia vitripennis]	1.00E-168	57.61
AjapOR70	g9525	395	Full	6	XP_014235093.1 odorant receptor Or2-like [Trichogramma pretiosum]	1.00E-44	27.25
AjapOR71	g9944	395	Full	6	XP_016845831.1 odorant receptor 207 isoform X2 [Nasonia vitripennis]	6.00E-79	35.79
AjapOR72	g10132	395	3' lost	7	NP_001177708.1 odorant receptor 241 [Nasonia vitripennis]	3.00E-163	60.84
AjapOR73	g12298	395	3' lost	6	XP_016836814.1 odorant receptor 49b isoform X3 [Nasonia vitripennis]	2.00E-120	50.64
AjapOR74	g12460	395	3' lost	5	NP_001177547.1 odorant receptor 146 [Nasonia vitripennis]	9.00E-157	57.87
AjapOR75	g10949	394	Full	6	NP_001177548.1 odorant receptor 147 [Nasonia vitripennis]	9.00E-123	47.34

AjapOR76	g12772	394	3' lost	4	NP_001177548.1 odorant receptor 147 [Nasonia vitripennis]	1.00E-104	40.05
AjapOR77	g2780	393	Full	7	NP_001177543.1 odorant receptor 140 [Nasonia vitripennis]	1.00E-172	62.94
AjapOR78	g13010	393	3' lost	7	NP_001177703.1 odorant receptor 160 [Nasonia vitripennis]	9.00E-160	60.05
AjapOR79	g5369	393	3' lost	7	NP_001177548.1 odorant receptor 147 [Nasonia vitripennis]	7.00E-126	45.67
AjapOR80	g10529	392	Full	7	NP_001177549.1 odorant receptor 151 [Nasonia vitripennis]	2.00E-155	54.59
AjapOR81	g10171	392	Full	6	NP_001177552.1 odorant receptor 161 [Nasonia vitripennis]	4.00E-138	48.85
AjapOR82	g10391	391	Full	6	NP_001177711.1 odorant receptor 296 [Nasonia vitripennis]	4.00E-133	53.51
AjapOR83	g10525	391	Full	6	NP_001177711.1 odorant receptor 296 [Nasonia vitripennis]	1.00E-147	53.03
AjapOR84	g8436	391	Full	6	NP_001177711.1 odorant receptor 296 [Nasonia vitripennis]	4.00E-149	52.62
AjapOR85	g12584	391	3' lost	4	NP_001164418.1 odorant receptor 246 [Nasonia vitripennis]	0	64.57
AjapOR86	g9827	390	Full	6	NP_001177703.1 odorant receptor 160 [Nasonia vitripennis]	3.00E-154	55.53
AjapOR87	g7685	390	3' lost	5	NP_001177708.1 odorant receptor 241 [Nasonia vitripennis]	2.00E-129	47.66
AjapOR88	g10367	389	Full	6	NP_001177703.1 odorant receptor 160 [Nasonia vitripennis]	2.00E-148	52.13
AjapOR89	g10741	389	Full	6	XP_023246772.1 odorant receptor 30a-like [Copidosoma floridanum]	5.00E-81	38.46
AjapOR90	g8778	389	Full	6	XP_023247720.1 odorant receptor Or1-like [Copidosoma floridanum]	4.00E-163	62.13
AjapOR91	g8456	389	3' lost	7	NP_001177601.1 odorant receptor 256 [Nasonia vitripennis]	3.00E-112	45.43
AjapOR92	g6970	388	Full	7	NP_001164399.1 odorant receptor 92 [Nasonia vitripennis]	2.00E-158	63.4
AjapOR93	g9272	388	Full	6	XP_023247720.1 odorant receptor Or1-like [Copidosoma floridanum]	8.00E-149	55.86
AjapOR94	g13245	388	3' lost	5	NP_001177548.1 odorant receptor 147 [Nasonia vitripennis]	6.00E-92	38.08
AjapOR95	g9201	386	Full	6	XP_015593532.1 odorant receptor 13a isoform X2 [Cephus cinctus]	2.00E-100	42.93
AjapOR96	g11068	385	3' lost	6	XP_031784325.1 odorant receptor 166 isoform X1 [Nasonia vitripennis]	1.00E-127	48.31
AjapOR97	g10882	384	Full	5	NP_001164396.1 odorant receptor 80 [Nasonia vitripennis]	2.00E-164	63.71
AjapOR98	g9800	384	Full	5	NP_001164396.1 odorant receptor 80 [Nasonia vitripennis]	5.00E-168	63.71
AjapOR99	g9991	383	Full	8	NP_001177600.1 odorant receptor 255 [Nasonia vitripennis]	3.00E-89	37.37
AjapOR100	g7297	383	Full	7	NP_001177708.1 odorant receptor 241 [Nasonia vitripennis]	8.00E-154	55.38
AjapOR101	g7949	381	Full	5	NP_001177509.1 odorant receptor 69 [Nasonia vitripennis]	3.00E-136	51.05

AjapOR102	g9866	380	Full	6	NP_001177503.1 odorant receptor 62 [Nasonia vitripennis]	3.00E-169	69.11
AjapOR103	g13027	380	3' lost	6	NP_001177546.1 odorant receptor 145 [Nasonia vitripennis]	2.00E-86	39.6
AjapOR104	g14390	380	5',3' lost	6	XP_016836814.1 odorant receptor 49b isoform X3 [Nasonia vitripennis]	3.00E-143	54.45
AjapOR105	g10625	378	Full	6	XP_016836814.1 odorant receptor 49b isoform X3 [Nasonia vitripennis]	9.00E-123	51.45
AjapOR106	g9922	378	Full	6	NP_001177507.1 odorant receptor 67 [Nasonia vitripennis]	2.00E-143	58.36
AjapOR107	g7488	377	Full	7	NP_001177708.1 odorant receptor 241 [Nasonia vitripennis]	2.00E-129	47.66
AjapOR108	g9447	377	Full	6	NP_001177476.1 odorant receptor 21 [Nasonia vitripennis]	2.00E-140	57.06
AjapOR109	g11490	377	Full	5	NP_001177600.1 odorant receptor 255 [Nasonia vitripennis]	3.00E-62	36.34
AjapOR110	g9621	376	Full	5	NP_001164670.2 odorant receptor 76 [Nasonia vitripennis]	5.00E-107	45.21
AjapOR111	g11753	373	Full	6	NP_001177534.1 odorant receptor 125 [Nasonia vitripennis]	8.00E-169	65.85
AjapOR112	g13299	373	Full	6	QGW50402.1 odorant receptor 77 [Chouioia cunea]	7.00E-79	66.16
AjapOR113	g10213	371	3' lost	6	NP_001177547.1 odorant receptor 146 [Nasonia vitripennis]	1.00E-93	39.57
AjapOR114	g14311	370	3' lost	7	NP_001177556.1 odorant receptor 170 [Nasonia vitripennis]	3.00E-65	34.45
AjapOR115	g13298	370	Full	5	XP_011501530.1 PREDICTED: odorant receptor 13a-like [Ceratosolen solmsi marchali]	8.00E-139	55.41
AjapOR116	g9639	366	Full	7	XP_031784326.1 odorant receptor 175 isoform X1 [Nasonia vitripennis]	2.00E-104	43.29
AjapOR117	g14812	366	3' lost	5	XP_011501530.1 PREDICTED: odorant receptor 13a-like [Ceratosolen solmsi marchali]	5.00E-166	63.49
AjapOR118	g10666	365	Full	6	XP_011496971.1 PREDICTED: odorant receptor 46a, isoform A-like [Ceratosolen solmsi marchali]	2.00E-138	54.67
AjapOR119	g12923	364	3' lost	7	NP_001177548.1 odorant receptor 147 [Nasonia vitripennis]	1.00E-152	55.77
AjapOR120	g13507	361	3' lost	5	NP_001177700.1 odorant receptor 149 [Nasonia vitripennis]	1.00E-107	49.61
AjapOR121	g13714	359	3' lost	5	NP_001177492.1 odorant receptor 45 [Nasonia vitripennis]	3.00E-160	66.77
AjapOR122	g12890	354	3' lost	6	XP_031784326.1 odorant receptor 175 isoform X1 [Nasonia vitripennis]	1.00E-98	41.93
AjapOR123	g10195	349	Full	6	NP_001164670.2 odorant receptor 76 [Nasonia vitripennis]	5.00E-107	44.13
AjapOR124	g7066	348	3' lost	5	NP_001177568.1 odorant receptor 192 [Nasonia vitripennis]	1.00E-171	67.24
AjapOR125	g13604	347	3' lost	5	NP_001177552.1 odorant receptor 161 [Nasonia vitripennis]	7.00E-141	55.33
AjapOR126	g14142	347	3' lost	5	XP_011500486.1 PREDICTED: odorant receptor 13a-like [Ceratosolen solmsi marchali]	6.00E-71	39.18
AjapOR127	g14613	347	3' lost	4	NP_001177552.1 odorant receptor 161 [Nasonia vitripennis]	1.00E-117	49.71

AjapOR128	g15060	345	3' lost	5	NP_001177556.1 odorant receptor 170 [Nasonia vitripennis]	8.00E-67	35.88
AjapOR129	g13812	342	3' lost	4	NP_001164418.1 odorant receptor 246 [Nasonia vitripennis]	4.00E-139	61.95
AjapOR130	g14603	341	3' lost	4	NP_001177548.1 odorant receptor 147 [Nasonia vitripennis]	8.00E-69	37.24
AjapOR131	g8497	340	3' lost	5	NP_001177711.1 odorant receptor 296 [Nasonia vitripennis]	2.00E-105	53.38
AjapOR132	g13342	339	3' lost	5	NP_001177505.1 odorant receptor 65 [Nasonia vitripennis]	2.00E-62	36.28
AjapOR133	g9941	337	Full	6	NP_001177555.1 odorant receptor 167 [Nasonia vitripennis]	3.00E-60	36.68
AjapOR134	g14704	337	Full	5	XP_011496971.1 PREDICTED: odorant receptor 46a, isoform A-like [Ceratosolen solmsi marchali]	6.00E-103	43.53
AjapOR135	g14503	332	3' lost	5	XP_031784326.1 odorant receptor 175 isoform X1 [Nasonia vitripennis]	3.00E-83	40.92
AjapOR136	g12026	331	Full	7	XP_016837030.1 odorant receptor 146 isoform X1 [Nasonia vitripennis]	3.00E-117	52.29
AjapOR137	g10442	331	3' lost	5	NP_001177700.1 odorant receptor 149 [Nasonia vitripennis]	3.00E-91	43.33
AjapOR138	g7822	327	Full	5	XP_016845869.1 odorant receptor 232 isoform X1 [Nasonia vitripennis]	6.00E-70	36.96
AjapOR139	g14841	323	Full	5	NP_001177700.1 odorant receptor 149 [Nasonia vitripennis]	4.00E-67	40.68
AjapOR140	g7141	318	Full	5	NP_001177703.1 odorant receptor 160 [Nasonia vitripennis]	5.00E-167	67.78
AjapOR141	g11255	317	Full	5	XP_014238328.2 odorant receptor 22c-like [Trichogramma pretiosum]	2.00E-82	48.7
AjapOR142	g11533	315	3' lost	5	NP_001177700.1 odorant receptor 149 [Nasonia vitripennis]	3.00E-142	66.56
AjapOR143	g8421	314	3' lost	4	NP_001177548.1 odorant receptor 147 [Nasonia vitripennis]	4.00E-100	48.9
AjapOR144	g6346	311	3' lost	6	XP_032452007.1 odorant receptor 49b isoform X4 [Nasonia vitripennis]	2.00E-95	49.83
AjapOR145	g11223	311	3' lost	4	NP_001177619.1 odorant receptor 286 [Nasonia vitripennis]	3.00E-136	59.68
AjapOR146	g7327	306	Full	5	XP_014238328.2 odorant receptor 22c-like [Trichogramma pretiosum]	1.00E-80	44.19
AjapOR147	g13814	304	3' lost	5	NP_001177558.1 odorant receptor 175 [Nasonia vitripennis]	5.00E-72	39.46
AjapOR148	g5384	301	3' lost	5	NP_001177521.1 odorant receptor 102 [Nasonia vitripennis]	2.00E-128	59.09
AjapOR149	g11705	298	3' lost	5	NP_001177703.1 odorant receptor 160 [Nasonia vitripennis]	1.00E-37	30.31
AjapOR150	g8852	289	Full	5	XP_031784326.1 odorant receptor 175 isoform X1 [Nasonia vitripennis]	2.00E-56	35.44
AjapOR151	g8060	287	Full	5	NP_001177486.1 odorant receptor 35 [Nasonia vitripennis]	1.00E-145	71.97
AjapOR152	g7924	283	Full	5	XP_032453632.1 odorant receptor 65 isoform X1 [Nasonia vitripennis]	3.00E-116	60.81
AjapOR153	g15345	276	3' lost	4	NP_001177548.1 odorant receptor 147 [Nasonia vitripennis]	1.00E-54	34.24

AjapOR154	g16334	276	3' lost	4	NP_001177548.1 odorant receptor 147 [Nasonia vitripennis]	7.00E-56	35.66
AjapOR155	g14305	270	3' lost	5	NP_001177643.1 odorant receptor 288 [Nasonia vitripennis]	2.00E-147	75.46
AjapOR156	g15814	269	3' lost	5	NP_001177556.1 odorant receptor 170 [Nasonia vitripennis]	7.00E-42	36.72
AjapOR157	g10360	265	3' lost	4	NP_001177548.1 odorant receptor 147 [Nasonia vitripennis]	1.00E-81	47.55
AjapOR158	g17055	257	3' lost	3	NP_001177557.1 odorant receptor 173 [Nasonia vitripennis]	2.00E-64	44.67
AjapOR159	g5912	256	3' lost	3	NP_001177548.1 odorant receptor 147 [Nasonia vitripennis]	8.00E-83	48.24
AjapOR160	g16038	251	3' lost	4	NP_001177703.1 odorant receptor 160 [Nasonia vitripennis]	4.00E-119	75.57
AjapOR161	g14003	247	3' lost	0	NP_001177511.1 odorant receptor 79 [Nasonia vitripennis]	5.00E-81	52.89
AjapOR162	g18874	241	3' lost	0	XP_014602640.1 PREDICTED: odorant receptor 22c-like [Polistes canadensis]	1.00E-49	38.96
AjapOR163	g14329	235	3' lost	5	NP_001177548.1 odorant receptor 147 [Nasonia vitripennis]	2.00E-72	52.94
AjapOR164	g16065	230	3' lost	3	XP_011504814.1 PREDICTED: odorant receptor 2a-like [Ceratosolen solmsi marchali]	2.00E-90	57.73
AjapOR165	g18298	225	3' lost	3	NP_001177622.1 odorant receptor 294 [Nasonia vitripennis]	1.00E-60	45.5
AjapOR166	g22905	224	3' lost	3	XP_031778255.1 odorant receptor 161 isoform X2 [Nasonia vitripennis]	4.00E-66	47.3
AjapOR167	g13926	213	3' lost	3	XP_031778255.1 odorant receptor 161 isoform X2 [Nasonia vitripennis]	6.00E-55	45.88
AjapOR168	g17251	213	3' lost	2	NP_001177589.1 odorant receptor 230 [Nasonia vitripennis]	1.00E-31	33.33
AjapOR169	g13497	211	3' lost	4	NP_001177548.1 odorant receptor 147 [Nasonia vitripennis]	4.00E-37	35.24
AjapOR170	g18656	202	3' lost	3	NP_001177576.1 odorant receptor 204 [Nasonia vitripennis]	1.00E-79	60.89
AjapOR171	g22400	200	3' lost	2	NP_001164404.1 odorant receptor 141 [Nasonia vitripennis]	7.00E-65	54.55
AjapOR172	g19049	197	3' lost	3	NP_001177701.1 odorant receptor 153 [Nasonia vitripennis]	2.00E-61	51.89
AjapOR173	g28222	197	3' lost	3	NP_001177548.1 odorant receptor 147 [Nasonia vitripennis]	2.00E-36	42.11
AjapOR174	g26395	192	3' lost	2	NP_001177547.1 odorant receptor 146 [Nasonia vitripennis]	2.00E-28	41.85
AjapOR175	g18727	183	3' lost	2	XP_023318597.1 odorant receptor 22c-like [Trichogramma pretiosum]	2.00E-34	48.39
AjapOR176	g21451	183	3' lost	2	XP_023313559.1 odorant receptor 82a-like [Trichogramma pretiosum]	5.00E-57	56.02
AjapOR177	g21586	183	3' lost	2	NP_001177575.1 odorant receptor 203 [Nasonia vitripennis]	3.00E-50	46.99
AjapOR178	g8935	177	3' lost	2	XP_031778255.1 odorant receptor 161 isoform X2 [Nasonia vitripennis]	1.00E-61	53.67
AjapOR179	g35089	165	3' lost	3	NP_001177700.1 odorant receptor 149 [Nasonia vitripennis]	6.00E-58	59.39

AjapOR180	g35969	162	3' lost	3	NP_001164670.2 odorant receptor 76 [Nasonia vitripennis]	6.00E-40	42.59
AjapOR181	g36879	155	3' lost	1	XP_031778255.1 odorant receptor 161 isoform X2 [Nasonia vitripennis]	1.00E-56	57.05
AjapOR182	g26739	142	3' lost	3	NP_001177701.1 odorant receptor 153 [Nasonia vitripennis]	2.00E-37	49.65
AjapOR183	g12347	125	3' lost	0	XP_023319411.1 putative odorant receptor 83c [Trichogramma pretiosum]	2.00E-22	45.08

Table S3 Sequence information of putative ionotropic receptors in *Anastatus japonicus*.

Name	Unigene reference	ORF (aa)	Status	TMD (No.)	BLASTx annotation	E_value	Identity (%)
AjapNmdar2	g1243	1004	Full	5	XP_031783802.1 glutamate receptor ionotropic, NMDA 2B isoform X3 [Nasonia vitripennis]	0	89.79
AjapIR8a	g3530	900	Full	3	XP_008206325.2 glutamate receptor ionotropic, kainate 1 [Nasonia vitripennis]	0	84.64
AjapIR93a	g4446	899	3' lost	3	XP_016841100.1 ionotropic receptor 93a isoform X3 [Nasonia vitripennis]	0	67.69
AjapCG3822	g5547	769	3' lost	3	XP_035728796.1 glutamate receptor ionotropic, kainate 2-like isoform X3 [Vespa mandarinia]	0	84.86
AjapIR64a.1	g1010	670	Full	3	XP_011502679.1 PREDICTED: glutamate receptor ionotropic, kainate 5-like [Ceratosolen solmsi marchali]	0	64.06
AjapIR75d.1	g4668	647	Full	3	XP_011502004.1 PREDICTED: glutamate receptor ionotropic, kainate 2-like [Ceratosolen solmsi marchali]	0	67.59
Ajap84a	g6475	643	Full	3	XP_031787913.1 glutamate receptor [Nasonia vitripennis]	0	70.54
AjapIR64a.2	g335	637	Full	3	XP_016843433.1 ionotropic receptor 75a-like isoform X1 [Nasonia vitripennis]	0	75
AjapIR75d.2	g6244	558	3' lost	4	XP_031783663.1 glutamate receptor ionotropic, delta-2 isoform X1 [Nasonia vitripennis]	0	75.32
AjapGlu-R1B	g6866	526	Full	3	XP_008215073.2 glutamate receptor 1 isoform X3 [Nasonia vitripennis]	0	94.68
AjapIR25a.1	g5170	487	Full	3	XP_031786896.1 ionotropic receptor 25a isoform X2 [Nasonia vitripennis]	1.00E-165	55.74
AjapIR75f.1	g5829	486	3' lost	4	XP_016843278.1 ionotropic receptor 75a isoform X1 [Nasonia vitripennis]	3.00E-166	52.92
AjapIR25a.2	g430	483	Full	3	XP_001603675.1 ionotropic receptor 25a-like [Nasonia vitripennis]	0	69.77
AjapCG5621	g10720	458	Full	3	XP_031781823.1 glutamate receptor ionotropic, kainate 2 [Nasonia vitripennis]	0	80.04
AjapIR75f.2	g5934	399	Full	4	XP_031777892.1 ionotropic receptor 75a-like [Nasonia vitripennis]	2.00E-152	51.92
AjapGlu-R1	g16318	340	5',3' lost	0	XP_001600998.4 glutamate receptor 1 isoform X2 [Nasonia vitripennis]	0	83.53
AjapNmdar1	g19697	150	3' lost	1	XP_011496545.1 PREDICTED: glutamate [NMDA] receptor subunit 1 [Ceratosolen solmsi marchali]	1.00E-45	80.31
